# Exosomal circSIPA1L3-mediated intercellular communication contributes to glucose metabolic reprogramming and progression of triple negative breast cancer

**DOI:** 10.1186/s12943-024-02037-4

**Published:** 2024-06-08

**Authors:** Yiran Liang, Fangzhou Ye, Dan Luo, Li Long, Yajie Wang, Yuhan Jin, Lei Wang, Yaming Li, Dianwen Han, Bing Chen, Wenjing Zhao, Lijuan Wang, Qifeng Yang

**Affiliations:** 1 Department of Breast Surgery, General Surgery, Qilu Hospital of Shandong University, Wenhua Xi Road No. 107, Jinan, Shandong 250012 P.R. China; 2 Department of Breast Surgery, Mianyang Central Hospital, Mianyang, Sichuan 621000 P.R. China; 3 Biological Resource Center, Qilu Hospital of Shandong University, Jinan, Shandong 250012 P.R. China; 4 Research Institute of Breast Cancer, Shandong University, Jinan, Shandong 250012 P.R. China

**Keywords:** Breast cancer, Glycolysis, Stemness, Exosome, circSIPA1L3, IGF2BP3

## Abstract

**Background:**

Breast cancer is the most common malignant tumor, and metastasis remains the major cause of poor prognosis. Glucose metabolic reprogramming is one of the prominent hallmarks in cancer, providing nutrients and energy to support dramatically elevated tumor growth and metastasis. Nevertheless, the potential mechanistic links between glycolysis and breast cancer progression have not been thoroughly elucidated.

**Methods:**

RNA-seq analysis was used to identify glucose metabolism-related circRNAs. The expression of circSIPA1L3 in breast cancer tissues and serum was examined by qRT-PCR, and further assessed its diagnostic value. We also evaluated the prognostic potential of circSIPA1L3 by analyzing a cohort of 238 breast cancer patients. Gain- and loss-of-function experiments, transcriptomic analysis, and molecular biology experiments were conducted to explore the biological function and regulatory mechanism of circSIPA1L3.

**Results:**

Using RNA-seq analysis, circSIPA1L3 was identified as the critical mediator responsible for metabolic adaption upon energy stress. Gain- and loss-of-function experiments revealed that circSIPA1L3 exerted a stimulative effect on breast cancer progression and glycolysis, which could also be transported by exosomes and facilitated malignant behaviors among breast cancer cells. Significantly, the elevated lactate secretion caused by circSIPA1L3-mediated glycolysis enhancement promoted the recruitment of tumor associated macrophage and their tumor-promoting roles. Mechanistically, EIF4A3 induced the cyclization and cytoplasmic export of circSIPA1L3, which inhibited ubiquitin-mediated IGF2BP3 degradation through enhancing the UPS7-IGF2BP3 interaction. Furthermore, circSIPA1L3 increased mRNA stability of the lactate export carrier SLC16A1 and the glucose intake enhancer RAB11A through either strengthening their interaction with IGF2BP3 or sponging miR-665, leading to enhanced glycolytic metabolism. Clinically, elevated circSIPA1L3 expression indicated unfavorable prognosis base on the cohort of 238 breast cancer patients. Moreover, circSIPA1L3 was highly expressed in the serum of breast cancer patients and exhibited high diagnostic value for breast cancer patients.

**Conclusions:**

Our study highlights the oncogenic role of circSIPA1L3 through mediating glucose metabolism, which might serve as a promising diagnostic and prognostic biomarker and potential therapeutic target for breast cancer.

**Supplementary Information:**

The online version contains supplementary material available at 10.1186/s12943-024-02037-4.

## Background

Breast cancer ranks as the most common malignancy and second leading cause of cancer-related deaths among women worldwide [1]. Despite the improvements in early detection and multimodal therapy, the prognosis of breast cancer patients is still unsatisfactory. Metastasis is the crucial cause of poor prognosis, and the average 5-year survival rate of patients with metastatic breast cancer is only 25% [2, 3]. Triple negative breast cancer (TNBC) is one of the histological subtypes of breast cancer, characterized by rapid progression, strong invasiveness, high potential of distant metastasis, and poor prognosis [4]. Although the proportion of TNBC in breast cancer is only 15–20%, it accounts for more than 50% of metastatic breast cancers. The metastasis of breast cancer is a complex process characterized by genetic and epigenetic abnormalities, therefore, a better comprehension of the molecular mechanisms underlying breast cancer progression would represent an essential step in expanding the treatment options and optimizing patient outcomes.

Previous studies reported that the glucose concentration in tumor tissues is lower compared to normal tissues, especially in rapidly growing tumors, confronting with severe nutrient deficient environment [5, 6]. In order to meet the requirements of cell proliferation and metastasis, tumor cells developed obviously different metabolism pattern from that of normal cells, which has been recognized as a hallmark of cancers [7]. Aerobic glycolysis, also termed Warburg effect, is one prominent phenomenon in cancers, wherein cancer cells tend to obtain energy through glycolysis rather than oxidative phosphorylation even in sufficient oxygen environment [8]. Increasing evidence supported that aerobic glycolysis is closely related to the high risk of metastasis and poor prognosis of various tumors, such as liver cancer [9], colon cancer [10] and gastric cancer [11]. As a highly malignant subtype, TNBC might have an enhanced demand for energy and nutrients, indicating the critical effect of aerobic glycolysis on the progression of TNBC. Nevertheless, the specific role and precise mechanisms related to glycolysis in breast cancer remain mostly unknown.

It has been broadly reported that non-coding RNAs were involved in various tumor progression [12, 13]. Among them, circular RNA (circRNA) is generated from the back-splicing of pre-mRNA to form covalently closed transcripts [14], featured by high stability, abundance, and conservation. Mounting evidence highlights the significant roles of circRNAs in cancer-related biological processes [15–17]. Exosomes, identified as membrane-bound extracellular vesicles with a size range between 40 and 120 nm [18], could modulate tumor microenvironment [19] and achieve intracellular communication by transmitting various biologically active molecules, exerting a significant influence on tumor growth, immune response, metabolic reprogramming and drug resistance in cancer development [20]. During this course, exosome-capsuled circRNAs have attracted great attention of researchers, which could shape the behaviors of surrounded cells followed up with an altered tumor niche [21, 22]. However, whether exosomal circRNAs could mediate breast cancer progression through modulating aerobic glycolysis and the underlying mechanism require to be further determined. Under these circumstances, we performed RNA-seq analysis to identify glucose metabolism-related circRNAs, and found that circSIPA1L3 was significantly induced by energy stress and correlated with poor prognosis of breast cancer patients. Additionally, circSIPA1L3 could be packaged into exosomes and transmitted to surrounding tumor cells, further facilitating breast cancer progression. Mechanistically, EIF4A3 was involved in the upregulation of circSIPA1L3, leading to increased expression of IGF2BP3 and its target genes, including the lactate export carrier SLC16A1 and the glucose intake enhancer RAB11A, which synergetically further exerted stimulative effects on breast cancer stemness and progression by enhancing glycolysis. Taken together, our data delineated the novel roles of exosomal circSIPA1L3 in supporting glycolysis for cancer progression, with promise for use in clinical intervention of breast cancer as a potential therapeutic target.

## Methods

### Patients and samples

The breast cancer tissues and normal tissues were obtained from breast cancer patients undergoing surgery in Qilu Hospital of Shandong University. Survival time refers to the time from operation date to the date of last follow-up or death. This research was approved by the Ethical Committee of Qilu Hospital of Shandong University, and informed consents from all of patients were obtained.

### RNA-seq

Total RNA was isolated using TRIzol reagent (Invitrogen, USA) following the manufacturer’s protocol. The sample sequencing and analysis were performed by Cloud-Seq Biotech (Shanghai, China). The differentially expressed genes were assessed by the Bioconductor package edgeR.

### RNase R and actinomycin D treatment

2 µg of RNA was incubated with or without 3 U of RNase R for 20 min at 37 °C, and the enzyme was inactivated at 70 °C for 10 min. To detect the RNA stability, cells were treated with 5 µg/ml Actinomycin D (ActD, Sigma, USA) for 0, 4, 8, 12, and 24 h before RNA isolation. The circSIPA1L3 and SIPA1L3 mRNA levels were detected by qRT-PCR assay.

### Cytosolic/nuclear fraction assay

The PARIS™ Kit (Invitrogen, USA) was used to isolated the cytosolic and nuclear RNAs according to the manufacturer’s instructions.

### Fluorescence in situ hybridization (FISH) and immunofluorescent (IF)

The Cy3-labeled probe specific to circSIPA1L3 was designed and synthesized by GenePharma (Shanghai, China), and the FISH assay was performed using a fluorescence in situ hybridization kit (GenePharma, Shanghai, China). Briefly, breast cancer cells were fixed by 4% paraformaldehyde, permeabilized with Triton-100, and incubated with specific probes at 37℃ overnight in a dark condition. The next day, DAPI (Invitrogen, USA) was used to counterstain the nuclei. For IF assay, the transfected cells were washed with PBS for 3 times, fixed by 4% paraformaldehyde for 15 min, permeated in 0.2% Triton X-100 for 15 min, and blocked with 10% goat serum for 1 h at room temperature. Then the cells were incubated with primary antibodies at 4 °C overnight. The next day, the cells were washed by PBS, incubated with corresponding secondary fluorescent antibodies at room temperature for 2 h, and the nuclear were counterstained by DAPI. Images were obtained by a fluorescent microscope (Olympus, Japan).

### In situ hybridization (ISH)

The ISH assay was conducted using the Enhanced Sensitive ISH Detection kit I (BOSTER, China) following the manufacturer’s instruction. The specific digoxin-labeled circSIPA1L3 probe was designed and synthesized by GenePharma (Shanghai, China), and the sequence was as follows: GGGTCACAGCCATACTTCAGTCTCT. Briefly, the tissue sections were fixed with 4% paraformaldehyde for 15 min, blocked by 30% H2O2^+^ methanol (1:50) for 30 min, and digested by pepsin for 15–30 min in room temperature. Next, the sections were treated with prehybridization solution in 37 °C for 4 h and incubated with the specific digoxin-labeled circSIPA1L3 probe in 37 °C overnight. The next day, the sections were incubated with blocking reagent, biotinylation rat anti digoxin, SABC, and biotinylation peroxidase successively. The expression of circSIPA1L3 was visualized by DAB staining, and nucleus was stained by hematoxylin (Solarbio, China). Images were captured using a light microscope.

### Isolation and identification of exosomes

Exosomes were isolated through standard differential centrifugation steps. Briefly, the cellular supernatant was collected and centrifugated in 300×g, 2,000×g and 10,000×g for 70 min at 4℃ successively for cell debris removement, and in 100,000×g for 70 min at 4℃ for exosome collection. Then the sediment was washed by PBS one time to eliminate protein interference, and finally resuspended using PBS. For asepsis during in vitro and in vivo assay, the collected exosome suspension was filtered with 0.22 μm filters. The concentration of exosomes were determined using a BCA Protein Assay Kit (Beyotime, China). 10 µg exosomes were injected via tail veins after the subcutaneous injection of 5 × 10^6^ MDA-MB-231 cells into female BALB/c nude mice during in vivo assays, and 2 µg exosomes were added to 1 × 10^5^ recipient cells during in vitro assays. The morphology and size of exosomes were examined by transmission electron microscope (FEI Tecnai G2 Spirit, Thermo Scientific, USA) and nanoparticle tracking analysis (NTA) using ZetaView PMX 110 (Particle Metrix, Meerbusch, Germany). The expression of specific exosome markers was detected using western blot.

### Exosome uptake assay

Exosomes were extracted and fluorescently labelled using PKH26 red fluorescent labeling kit (Sigma). The labeled exosomes were added to breast cancer cells and incubated for 12 h. Images of the cells were captured by a fluorescence microscope (Olympus, Japan).

### Flow cytometry (FCM)

For apoptosis assay, transfected cells were collected and washed by PBS twice. According to the protocol of PE Annexin V Apoptosis Detection Kit I (BD Pharmingen), cells were resuspended by 1×binding buffer and stained with PE Annexin V and 7-AAD respectively for 1 h in the dark. For cell cycle assay, transfected cells were stained by cell cycle staining buffer (Multi Sciences, Hangzhou, China) for 30 min in the dark. For cell stemness assay, PE-CD24 (Invitrogen, USA) and FITC-CD44 (Invitrogen, USA) were used for detecting the ratio of CD44^+^CD24^−^ cells, and the ALDEFLUOR™ Kit (STEMCELL, Canada) was used to screen out the ratio of ALDH^+^ cells. Stained cells were washed to remove redundant dye or antibodies, and tested by a FACSCalibur flow cytometer (BD Biosciences, CA, USA).

### ATP levels and lactate production

1 × 10^6^ transfected or treated cells was used to detect the total intracellular ATP levels according to the protocol of ATP assay kit (Nanjing Jiancheng Bioengineering Institute). For the lactate production assay, lactic acid assay kit (Nanjing Jiancheng Bioengineering Institute) was used. The culture medium was changed to serum-free DMEM 24 h before detection, and the lactate production was measured in the culture supernatant.

### Glycolysis stress test

ECAR was measured using the Seahorse XF Glycolysis Stress Test kit (Agilent Technologies) according to the manufacturer’s protocol. Briefly, a total of 2 × 10^4^ transfected cells were seeded into an XF96 plate and incubated overnight. Then cells were washed using assay medium (non-buffered XF Base Medium Minimum DMEM), and cultured in stress test medium for 1 h before adding drug. Extracellular acidification rate (ECAR) was measured after the sequential addition of glucose (10 mM), oligomycin (2 μm) and 2-deoxyglucose (2-DG, 100 mM) under basal conditions with a Seahorse XFe96 detecting system.

### Sphere formation assay

The transfected or treated cells was seeded into Ultra-Low Attachment 96-well plate. The cells were cultured in DMEM/F12 with 20 ng/mL human EGF, 20 ng/mL fibroblast growth factor, B27 (1:50), 5 µg/mL insulin, 100 U/mL penicillin, and 100 mg/mL streptomycin. After 1 week, the formed spheres were captured using a light microscope, and the diameter was measured.

### Patient-derived organoid (PDO) culture and treatment

The minced tissues were digested for 2 h at 37 °C on an orbital shaker. The digestion medium was DMEM/F12 (Macgene, China) containing 1% BSA, ITS-G (BasalMedia Technologies Company, China), Y-27,632 (5 µM, MCE, USA), Primocin (Invitrogen, USA), HEPES (10 mM, Thermo, USA), hyaluronidase (1000 U/mL, Sigma-Aldrich, USA), collagenase I and III (Worthington, Italy). Subsequently, the digestion mixture was filtered with a 100 μm filter strainer, and centrifuged at 300 x g for 5 min at 4 °C. The cell pellets were resuspended with digestion termination solution (DMEM/F12 supplemented with 0.1% BSA and Primocin). After centrifugation, TAC buffer was applied to lyse the erythrocytes, and the sediment was washed twice using the digestion termination solution. The cell pellets were resuspended with growth factor-reduced Matrigel (R&D Systems, USA), diluted 1:3 in organoid culture medium. The organoid suspension was seeded into 48-well plate and transferred into the incubator for 60 min at 37 °C to allowed solidification. Then 300 µl organoid culture medium was added. The organoid culture medium was composed of DMEM/F12 medium supplemented with 0.5 µg/ml R-spondin-1 (BioLegend, USA), 0.5 µg/ml Neuregulin 1 (PeproTech, USA), 5 ng/ml human recombinant FGF7 (PeproTech, USA), 5 ng/ml human EGF (PeproTech, USA), 0.1 µg/mL human Noggin (PeproTech, USA), 20 ng/ml human FGF10 (PeproTech, USA), 500 nM A83-01 (Tocris Bioscience, USA), 5 µM Y-27,632 (Sigma-Aldrich, USA), 500 nM SB202190 (Sigma-Aldrich, USA), 1.25 mM N-acetyl-L-cysteine (Sigma-Aldrich, USA), 5 mM Nicotinamide (Sigma-Aldrich, USA), 1×Primocin (Invitrogen, USA), 1×GlutaMax (Invitrogen, USA), 1×HEPES (Thermo, USA), and 1×B27 (Gibco, USA). Fresh medium was replenished every three days, and TrypLE (Gibco, USA) was used for the passage of organoids. After 48 h post-seeding, circSIPA1L3-overexpressing or control lentiviral were used to infect these cells. The organoids were imaged and size was recorded using a light microscopy (20×, ZEISS).

### Animal experiments

For xenograft tumor assays, 5 × 10^6^ MDA-MB-231 stably overexpressing circSIPA1L3 or control vector pLCDH-ciR were subcutaneously injected into female BALB/c nude mice (4–6 weeks). After 1 month, the subcutaneous tumors were collected. The size of tumors was obtained by calculation: V = (length × width^2^)/2, and the weight of tumors was also measured. For the in vivo lung metastasis mode, cells were injected via the tail vein. After 6 weeks, the lungs were removed, and the metastatic nodes were counted. The tissues were sectioned for hematoxylin and eosin (HE), IHC, and ISH staining. All of the experimental procedures involving animals were approved by Shandong University Animal Care and Use Committee.

### Statistical analysis

Statistical analysis was performed by GraphPad Prism 8.0. All data were represented as mean ± standard deviation (SD). Data were represented as mean ± standard deviation (SD) from independent experiments with at least three biological replicates. Student’s t-test or one-way ANOVA was applied to evaluate the relationship between parametric variables. Chi-square test was used to analyze the relationships between nonparametric variables. Kaplan–Meier analysis was used to analyze the survival differences. *P* < 0.05 was regarded statistically significant.

The complete experimental protocols are described in the supplemental material. The corresponding sequences of siRNA and miRNA mimics were listed in Table S3. The sequences of primers are listed in Table S4. The primary and secondary antibodies are listed in Table S5.

## Results

### CircSIPA1L3 is a glucose metabolism-related circRNA in breast cancer

**Fig. 1:**
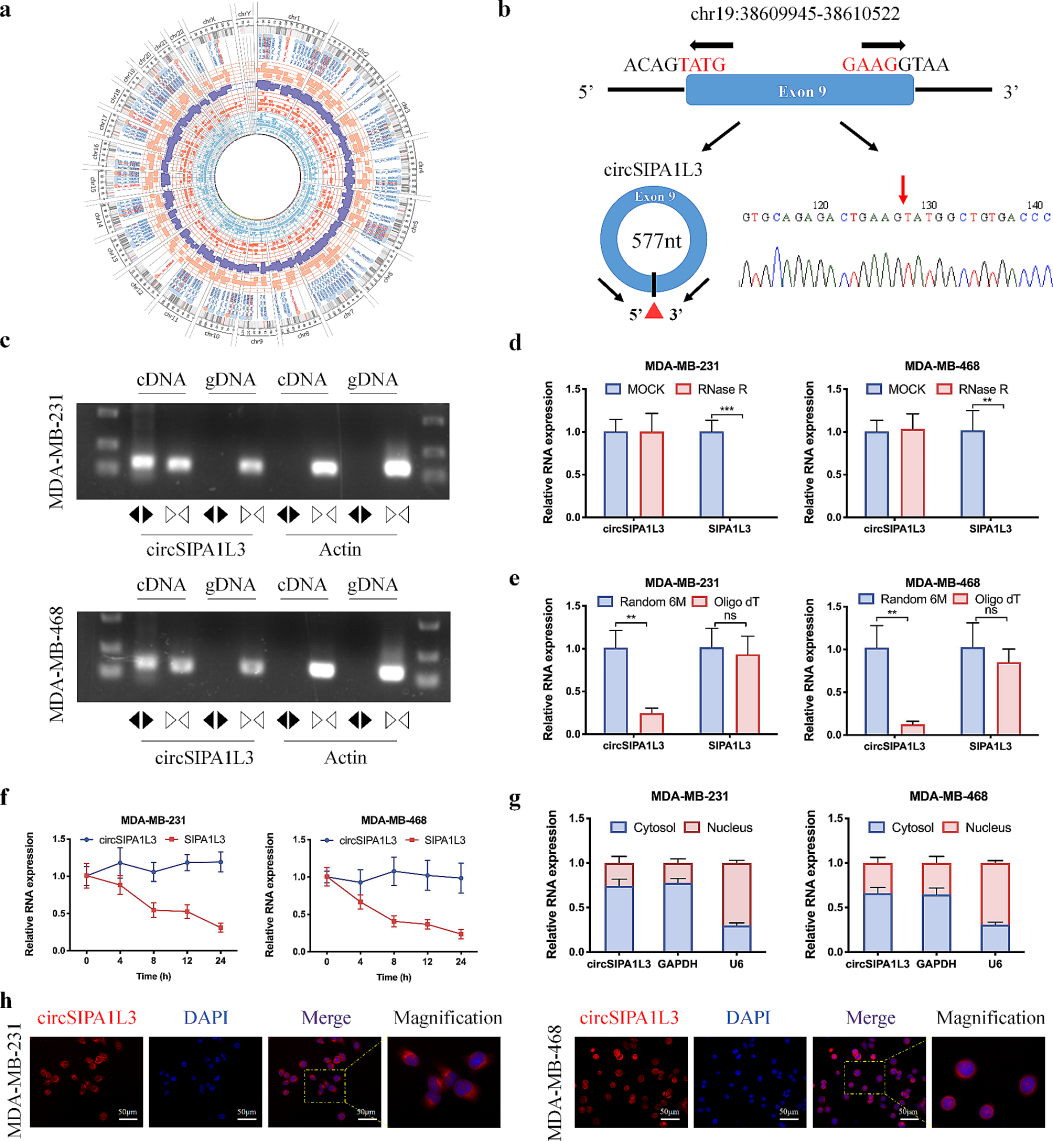
Expression profiles of circRNAs induced by glucose limitation and characterization of circSIPA1L3. (**a**) Circos plot represents the differentially expressed circRNAs between low glucose-treated breast cancer cells and control cells (|Log2 FC| ≥2). (**b**) The expression of circSIPA1L3 was detected by qRT-PCR in breast cancer cells cultured with normal glucose and limited glucose. (**c**) Schematic illustration of the genomic location and circularization of circSIPA1L3. The back-splice junction sequences of circSIPA1L3 were validated by Sanger sequencing. (**d**) The presence of circSIPA1L3 from cDNA and gDNA in breast cancer cells was detected by RT-PCR with divergent and convergent primers and agarose gel electrophoresis analysis. Actin was used as a linear control. (**e**) RNase treatment was used to evaluate the stability of circSIPA1L3 and SIPA1L3 mRNA in breast cancer cells. (**f**) The relative RNA levels of circSIPA1L3 and SIPA1L3 mRNA were measured by qRT-PCR after reverse transcription experiment using Random 6 M or oligo dT primers. (**g**) Actinomycin D treatment was used to assess the stability of circSIPA1L3 and SIPA1L3 mRNA in breast cancer cells. (**h**) Nuclear-cytoplasmic fractionation assay was performed to evaluate the intracellular localization of circSIPA1L3 in breast cancer cells. U6 was used as the nuclear internal reference and GAPDH was used as the cytoplasmic internal control. (**i**) The subcellular location of circSIPA1L3 was detected by FISH assay in breast cancer cells. The circSIPA1L3 probe was labelled with Cy3 (red), and nuclei were stained with DAPI (blue). (ns, *P* > 0.05, *P < 0.05, **P < 0.01)

To simulate the glucose deficient environment in rapidly growing tumors, breast cancer cells were cultured in low glucose condition to evaluate the effect on glycolysis. Interestingly, the lactate production and ATP levels in glucose-limited cells were upregulated compared to control cells (Figure S1A, B), indicating the enhanced glycolysis under energy stress. High-throughput sequencing was further performed in low glucose-cultured and normal cultured breast cancer cells to identify circRNAs potentially involved in glucose metabolism. After screening, 40 upregulated and 137 downregulated circRNAs were identified (Fig. 1a). Subsequently, qRT-PCR was used to verify the expression of top 6 upregulated circRNAs in low glucose-cultured breast cancer cells (Figure S1C). Hsa_circ_0005081 (termed circSIPA1L3), which was expressed at the highest level among these circRNAs, was selected for further study. Based on circBase and circBank databases, circSIPA1L3, a 577-nt circRNA, was located at chr19:38,609,945 − 38,610,522 and formed by the head-to-tail splicing of the exon 9 within SIPA1L3 gene locus (Fig. 1b). The back-spliced junction point of circSIPA1L3 was amplified with divergent primers and confirmed by Sanger sequencing (Fig. 1b). To further validate the circular characteristics of circSIPA1L3, PCR assay using convergent and divergent primers was performed. As expected, circSIPA1L3 could only be amplified by divergent primers from cDNA but not from genomic DNA in breast cancer cells (Fig. 1c). Moreover, circSIPA1L3 was more resistant to RNase R treatment compared to linear SIPA1L3 (Fig. 1d), further confirming its circular RNA structure. We also observed that almost no circSIPA1L3 was detected when oligo dT primers were applied compared to random primers, indicating the lack of 3′ polyadenylated tail (Fig. 1e). Furthermore, the ActD treatment assay validated the longer half-life of circSIPA1L than that of linear SIPA1L3 mRNA (Fig. 1f). In addition, nuclear/cytoplasmic fractionation assay and FISH assay revealed the predominantly cytoplasmic localization of circSIPA1L3 in breast cancer cells (Fig. 1g, h).

### CircSIPA1L3-induced aerobic glycolysis contributes to stemness and progression of breast cancer

We first explored the biological function of circSIPA1L3 in breast cancer. Two siRNAs were designed that specifically targeted the back-splicing site of circSIPA1L3 to knock down its expression without affecting the expression of linear SIPA1L3 mRNA (Figure S2A). The functional experiments revealed that circSIPA1L3 knockdown led to suppressed proliferation, migration, invasion, and angiogenesis-promoting abilities of breast cancer cells (Figure S2B-I), whereas circSIPA1L3 overexpression circSIPA1L3 overexpression in vitro and in vivo (Figure S3), indicating the tumor-promoting role of circSIPA1L3 in breast cancer.

**Fig. 2:**
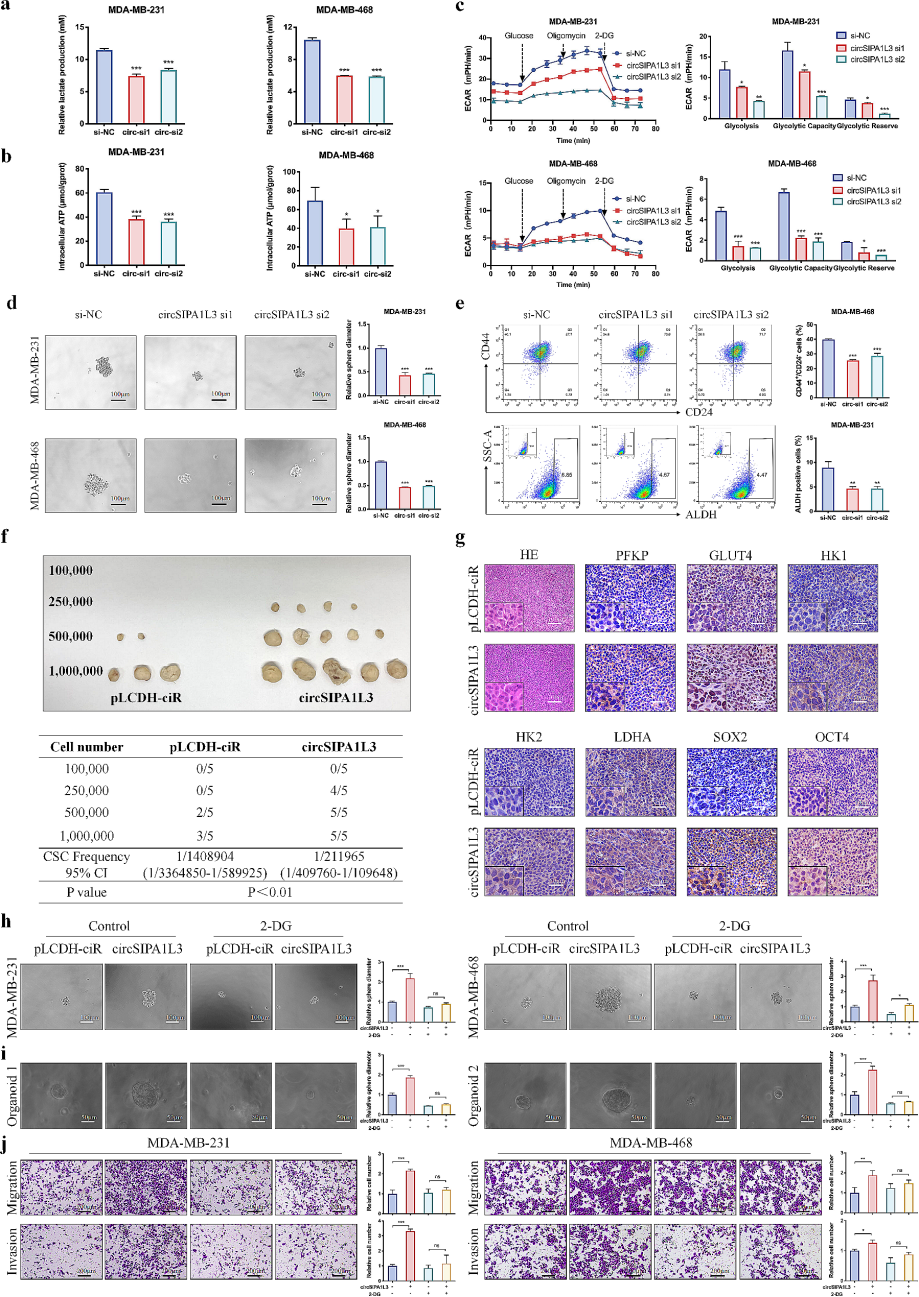
circSIPA1L3 promotes breast cancer progression through enhancing glycolysis. (**a**) The lactate production was evaluated in breast cancer cells transfected with NC or circSIPA1L3 siRNA. (**b**) The ATP levels were measured in breast cancer cells after circSIPA1L3 knockdown. (**c**) The extracellular acidification rate (ECAR) was detected using the “Seahorse analyzer” after circSIPA1L3 knockdown in breast cancer cells. The glycolysis, glycolytic capacity, and glycolytic reserve of indicated cells were calculated. (**d**) Tumor sphere formation capacity was measured to evaluate the effect of circSIPA1L3 knockdown on stemness of breast cancer cells. (**e**) Flow cytometric assays was performed to detect the percentage of CD44^+^CD24^−^ phenotype in MDA-MB-468 cells transfected with NC or circSIPA1L3 siRNA. ALDEFLUOR assay was used to evaluate the effect of circSIPA1L3 knockdown on the percentage of ALDH positive phenotype in MDA-MB-231 cells. (**f**) In vivo limited dilution xenograft assay was performed, and ELDA software was used to estimate the stem cell frequency. (**g**) HE and IHC assay was conducted to assess the expression of glycolysis- and stemness -related markers in the control or circSIPA1L3-overexpressing xenograft tissues. (**h**) Breast cancer cells were transfected with pLCDH-ciR or circSIPA1L3 overexpressing plasmids, and further treated with or without 2-DG. Tumor sphere formation assay was used to evaluate tumor stemness. (**i**) Patient-derived organoids infected using circSIPA1L3-overexpression or control retroviruses were treated with or without 2-DG, and the representative images were shown. (**j**) The migration and invasion abilities of indicated breast cancer cells were analyzed by transwell assay. (ns, *P* > 0.05, *P < 0.05, **P < 0.01, ***P < 0.001)

Subsequently, we examined the role of circSIPA1L3 in modulating glucose metabolism and underlying effect on breast cancer progression. CircSIPA1L3 knockdown inhibited the production of lactate and intracellular ATP, while circSIPA1L3 overexpression caused opposite results (Fig. 2a, b, Figure S4A, B). Furthermore, the extracellular acidification rate (ECAR) measurement indicated that circSIPA1L3 knockdown led to markedly lower level of glycolysis, glycolytic capacity, and glycolytic reserve compared with control cells, while upregulation of circSIPA1L3 caused opposite results (Fig. 2c, Figure S4C), further validating the enhanced glycolysis after circSIPA1L3 overexpression. The existence of stem cells is one of the major reasons for unlimited proliferative potential of breast cancer cells, and the relationship between tumor stemness and glycolysis has been well established [23, 24]. As expected, circSIPA1L3 knockdown led to reduced size of mammospheres derived from breast cancer cells, and decreased percentage of CD44^+^CD24^−^ or ALDH^+^ breast cancer cells (Fig. 2d, e, Figure S5A). Furthermore, IF assay indicated that the expression of stemness-related proteins, OCT4 and SOX2, was decreased in the spheroids derived from circSIPA1L3 knockdown breast cancer cells (Figure S5B). Western blot assay confirmed the lower protein levels of glucose metabolism, EMT, and stemness-related markers in circSIPA1L3 knockdown breast cancer cells than in control cell (Figure S5C). Consistently, circSIPA1L3 overexpression promoted the stemness property of breast cancer cells (Figure S6A-E). To gain insight into the promoting role of circSIPA1L3 in the stemness of breast cancer cells in vivo, xenograft experiments using a limiting dilution assay of MDA-MB-231 cells were conducted. The nude mice injected with 2.5 × 10^5^ circSIPA1L3 overexpressing cells had dramatically increased incidence of tumor initiation compared to control group. Moreover, tumor-forming frequency reached 100% when 5 × 10^5^ circSIPA1L3 overexpressing cells were injected, whereas injection of 5 × 10^5^ control cells only had 40% tumor-forming frequency (Fig. 2f). In addition, ELDA software [25] was used to calculate stem cell frequencies based on the number of mice with tumors, and showed that circSIPA1L3 overexpressing cells displayed a significant increase in stem cell frequency (1:211,965) compared with control cells (1:1,408,904). Finally, IHC assay confirmed the higher levels of glycolysis and stemness-related markers in circSIPA1L3 overexpressing tumors versus control tumors (Fig. 2g, Figure S6F).

We next investigated whether circSIPA1L3-induced glycolysis had effect on stemness and progression of breast cancer. The expression of circSIPA1L3 was inhibited when glycolysis was blocked using glycolysis inhibitor 2-DG (Figure S7A). Consistently, ectopic circSIPA1L3 expression promoted breast cancer cell viability and stemness; however, 2-DG treatment significantly weakened these effects (Fig. 2h; Figure S7B and S7C). We further examined the effect of circSIPA1L3-induced glycolysis on the growth of organoids derived from breast cancer patients, and obtained similar results (Fig. 2i). In addition, 2-DG treatment could significantly attenuate the enhanced migration and invasion abilities of breast cancer cells caused by circSIPA1L3 overexpression (Fig. 2j; Figure S7D). Given that lactate is associated with immune cell reeducation in the tumor microenvironment [26], we wondered whether cancer cell-produced lactate could modulate the function of tumor associated macrophages (TAMs). The lactate concentration was increased in conditioned medium (CM) from circSIPA1L3-overexpresing breast cancer cells, which could be significantly blocked by treatment with LDHA inhibitor sodium oxamate (OXM) (Figure S8A). Consistently, THP1 migration was enhanced in circSIPA1L3-overexpresing group; however, OXM treatment significantly weakened the effect (Figure S8B). To exclude the effect of cytokines/chemokines present in the media on THP1 migration, CM boiling method was performed to inactivate cytokines/chemokines without influence the character of lactate in the CM. As expected, the boiled CM could still promote migration ability of THP1 (Figure S8C). Furthermore, we treated THP1 with increasing concentration of OXM, and no change of THP1 migration was found, which eliminated the effect of OXM itself on THP1 migration (Figure S8D). Of note, TAMs treated with CM isolated from circSIPA1L3-overexpressing cells significantly promoted cell migration, while these effects could be abolished by OXM addition (Figure S8E), demonstrating that circSIPA1L3-induced glycolysis contributed to the tumor-promoting function of TAMs via lactate production. Collectively, these results providing compelling evidences that ectopic circSIPA1L3 expression-induced glycolysis is critical for breast cancer progression.

### Exosomal circSIPA1L3 facilitates glycolysis and progression of breast cancer in vitro and in vivo

**Fig. 3:**
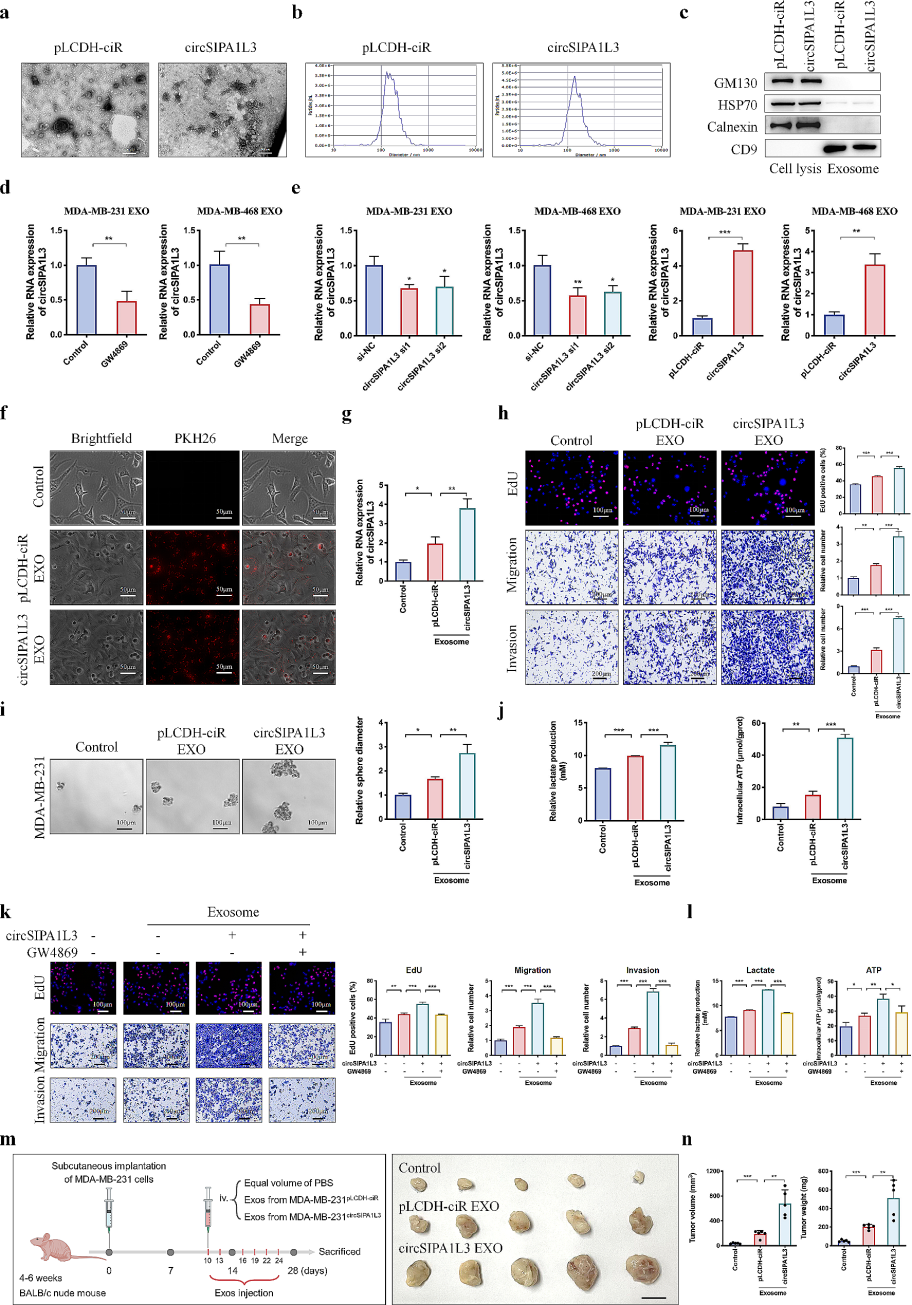
Exosome-transmitted circSIPA1L3 promotes progression and glycolysis of surrounding breast cancer cells. (**a**) Transmission electron microscopy detected the exosomes isolated from supernatants of MDA-MB-231 cells transfected with pLCDH-ciR or circSIPA1L3 plasmids via ultracentrifugation. Scale bars, 200 nm. (**b**) NTA analysis of the size distribution of isolated exosomes. (**c**) Western blot analysis showing the exosome markers. (**d**) The expression of circSIPA1L3 in exosomes isolated from supernatants of breast cancer cells treated with or without GW4869 was detected using qRT-PCR. (**e**) Breast cancer cells were transfected with circSIPA1L3 siRNAs or overexpressing plasmids, and the expression of circSIPA1L3 in isolated exosomes were measured. (**f**) Representative images showing the internalization of PKH26-labeled exosomes in breast cancer cells. (**g**) MDA-MB-231 cells were treated with pLCDH-ciR-EXOs or circSIPA1L3-EXOs, and the expression level of circSIPA1L3 was examined by qRT-PCR. (**h**) EdU and transwell assays were used to detect the effect of exosomal circSIPA1L3 on cell proliferation, migration and invasion abilities. (**i**) Tumor sphere formation assay was used to detect the effect of exosomal circSIPA1L3 on cell proliferation and tumor stemness. (**j**) The lactate production and ATP levels were evaluated in cells treated with pLCDH-ciR-EXOs or circSIPA1L3-EXOs. (**k**) EdU and transwell assays were used to detect the proliferation, migration and invasion abilities of MDA-MB-231 treated with exosomes derived from differently transfected cells upon treatment with or without GW4869. (**l**) The lactate production and ATP levels were evaluated in cells treated with exosomes derived from differently transfected cells upon treatment with or without GW4869. (**m**) Schematic diagram (left) showing that BALB/c nude mice were subcutaneously injected with MDA-MB-231 cells and subsequently treated with or without exosomes (10 µg, every three days). Images of xenograft tumors from different groups (right). Scale bar, 1 cm. (**n**) Tumor volumes (left) and tumor weights (right) of xenograft tumors from different groups. (*P < 0.05, **P < 0.01, ***P < 0.001)

Inspired by the above results, we wonder whether circSIPA1L3 could be loaded into exosomes and affect the function of surrounding recipient cells. Exosomes from the supernatants of MDA-MB-231 cells with or without circSIPA1L3 overexpression were isolated via ultracentrifugation, which exhibited similar typical membrane structure and size distribution as observed by transmission electron microscope (TEM) and nanoparticle tracker analysis (NTA) (Fig. 3a, b). The expression of exosome marker proteins was detected by western blot (Fig. 3c), validating the successful isolation of exosomes from breast cancer cells. Furthermore, we found that the expression of circSIPA1L3 was decreased in cells treated with GW4869 (Fig. 3d), a noncompetitive inhibitor of neutral sphingomyelinase (N-SMase) which inhibits exosome formation [27]. Moreover, the circSIPA1L3 expression in exosomes was increased in breast cancer cells with circSIPA1L3 overexpression, whereas circSIPA1L3 knockdown led to opposite results (Fig. 3e). Altogether, these results confirmed the existence of circSIPA1L3 in exosomes. To validate that exosomal circSIPA1L3 could be taken up by receptor cells, PKH26 staining was conducted and fluorescent-labeled exosomes were observed in the receptor cells (Fig. 3f). The circSIPA1L3 expression was increased in MDA-MB-231 cells that treated with exosomes highly expressing circSIPA1L3 than in control group (Fig. 3g). Moreover, we validated that circSIPA1L3-exosomes significantly enhanced the proliferation, migration, invasion, and stemness of MDA-MB-231 cells compared with the control group (Fig. 3h, i). Most noteworthy, the lactate secretion and ATP production of MDA-MB-231 were evidently elevated after treated with breast cancer cell-derived exosomes, especially in the circSIPA1L3 overexpression group (Fig. 3j), which indicated the role of exosomal circSIPA1L3 as an essential glycolysis regulator during breast cancer development. However, GW4869 treatment led to obviously attenuated malignant behaviors of MDA-MB-231 cells caused by circSIPA1L3-overexpressing exosomes (Fig. 3k, l). In xenograft tumors treated with circSIPA1L3-exosomes, obviously increased tumor volume and tumor weight were also observed (Fig. 3m, n). Overall, these results suggested that exosomal circSIPA1L3 could affect the progression of breast cancer through cell-cell communication.

### EIF4A3 induces the biogenesis and cytoplasmic export of circSIPA1L3

To explore the underlying mechanism of circSIPA1L3 upregulation, we predicted the potential RBP binding sites in the flanking regions of circSIPA1L3 by searching CircInteractome and circAltas databases. EIF4A3 was found to have the most possible binding sites in the upstream and downstream regions of circSIPA1L3, which could also be induced by low glucose (Figure S9A-D), indicating the regulatory potential of EIF4A3 on circSIPA1L3 upregulation under low glucose condition. RNA pulldown assay illustrated that EIF4A3 could bind to both the back-spliced junction site, upstream and downstream flanking sequence of circSIPA1L3 (Figure S9E, F). The RIP assay confirmed the binding between EIF4A3 and SIPA1L3 pre-mRNA (Figure S9G). In addition, EIF4A3 overexpression led to increased, while EIF4A3 knockdown caused decreased circSIPA1L3 expression in breast cancer cells (Figure S9H-J). Furthermore, the variation trend of circSIPA1L3 in exosomes after EIF4A3 overexpression or knockdown was consistent with the above results obtained from breast cancer cells (Figure S9K). Interestingly, the nuclear/cytoplasmic fractionation assay and FISH assay indicated that inhibition of EIF4A3 led to a clear reduction of cytoplasmic circSIPA1L3 levels in breast cancer cells (Figure S9L, M), indicating that EIF4A3 was responsible for the cytoplasmic export of circSIPA1L3. Furthermore, we identified a positive correlation between the expression of EIF4A3 and circSIPA1L3 based on our own cohort (Figure S9N).

The expression of EIF4A3 was upregulated in breast cancer tissues compared to normal tissues based on several public databases and specimens in our cohort, and EIF4A3 expression was increased with the grade and stage of breast cancer (Figure S10A, B). Moreover, the RNA and protein expression of EIF4A3 was also elevated in various kinds of cancers (Figure S10C). Significantly, higher EIF4A3 expression was associated with worse prognosis of breast cancer patients (Figure S10D), indicating the tumor-promoting role of EIF4A3. The functional experiments further validated that EIF4A3 knockdown could inhibit breast cancer cell proliferation, migration, and invasion (Figure S11). To validate that the observed EIF4A3-mediated phenotypes were facilitated by the dysregulated circSIPA1L3 expression, rescue experiments were performed. The results showed that EIF4A3 overexpression led to increased proliferation, migration, invasion, stemness, and glycolysis in breast cancer cell, which could be obviously reversed by circSIPA1L3 knockdown (Figure S12). These results collectively revealed that EIF4A3 could positively modulate the generation and cytoplasmic export of circSIPA1L3, which was responsible for the oncogenic role of circSIPA1L3 in breast cancer.

### circSIPA1L3 physically interacts with IGF2BP3 protein mainly through the RRM1-2 domain to regulate glycolysis of breast cancer cells

**Fig. 4:**
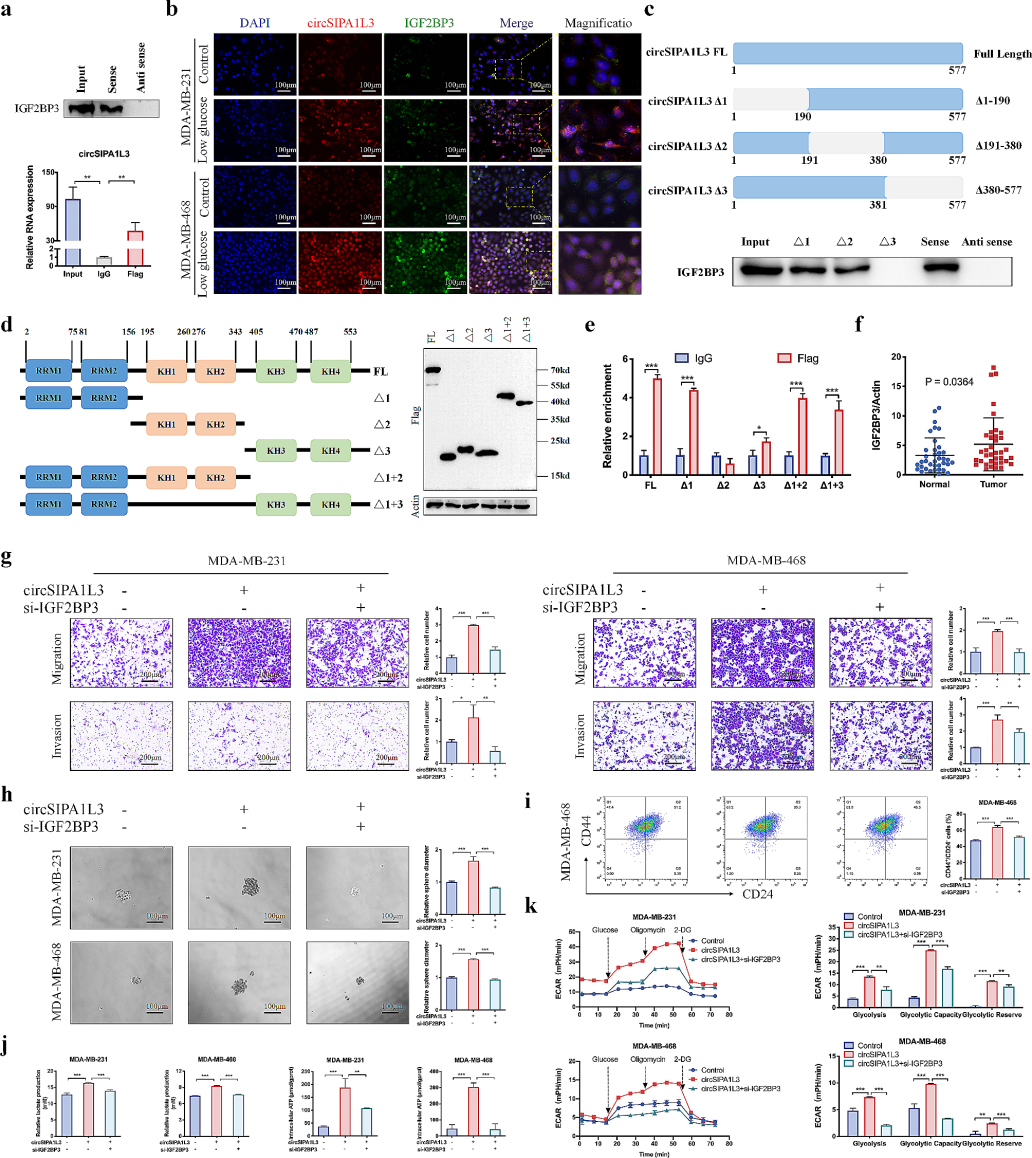
Emphasis Type=“Bold”>circSIPA1L3 interacts with IGF2BP3 protein through the KH domains, and IGF2BP3 participates in circSIPA1L3-mediated tumor-promoting functions. (**a**) RNA pulldown followed by western blot (top) and RIP assay (bottom) were used to verify the interaction between circSIPA1L3 and IGF2BP3. (**b**) FISH and IF assay was performed to evaluate the subcellular localization and expression of circSIPA1L3 and IGF2BP3 in breast cancer cells. Nuclei were stained with DAPI. (**c**) Western blot shows the expression of IGF2BP3 in samples pulled down by full length (FL) or truncated circSIPA1L3. (**d**) Schematic structures of IGF2BP3 proteins and five mutants (left). Western blot assay detects the expression of full length of IGF2BP3 and five mutants (right). (**e**) RIP and qRT-PCR assay was used to identify the binding region of IGF2BP3 for circSIPA1L3. (**f**) The expression of IGF2BP3 in normal tissues and breast cancer tissues were analyzed using qRT-PCR. (**g**) Transwell assay shows the migration and invasion abilities of breast cancer cells co-transfected with circSIPA1L3 overexpressing plasmids and IGF2BP3 siRNA as indicated. (**h**) Tumor sphere formation assay exhibits the stemness feature of breast cancer cells co-transfected with circSIPA1L3 overexpressing plasmids and IGF2BP3 siRNA as indicated. (**i**) Flow cytometric assays was performed to detect the percentage of CD44^+^CD24^−^ phenotype in MDA-MB-468 cells co-transfected with circSIPA1L3 overexpressing plasmids and IGF2BP3 siRNA as indicated. (**j**) The lactate production and ATP levels were detected in breast cancer cells co-transfected with circSIPA1L3 overexpressing plasmids and IGF2BP3 siRNA as indicated. (**k**) The extracellular acidification rate (ECAR) was measured in breast cancer cells co-transfected with circSIPA1L3 overexpressing plasmids and IGF2BP3 siRNA as indicated, and glycolysis, glycolytic capacity, and glycolytic reserve of indicated cells were calculated. (*P < 0.05, **P < 0.01, ***P < 0.001)

RNA pull-down assay and subsequent mass spectrometry assay were performed to explore the potential binding proteins of circSIPA1L3, and a total of 208 proteins only interacting with circSIPA1L3 sense probe were detected. After intersecting the results of mass spectrometry with the predicted RBPs based on several databases, the binding potential between circSIPA1L3 and IGF2BP3 was identified (Figure S13A-E), which was further validated by RNA pull-down and RIP assays (Fig. 4a, Figure S13F). In addition, FISH-IF analysis confirmed the cytoplasmic colocalization of endogenous circSIPA1L3 and IGF2BP3, and low glucose treatment could further enhance the interaction (Fig. 4b). Consistent with the elevated expression of circSIPA1L3, low glucose treatment led to increased expression of IGF2BP3 (Figure S13G, H). We further performed RNA pull-down assay to delineate the sites necessary for IGF2BP3 recruitment in circSIPA1L3 using wild-type or deletion mutant probes. The results indicated that 381–577 nt region of circSIPA1L3 was responsible for the association with IGF2BP3 (Fig. 4c). There are six major functional regions in IGF2BP3, including two RNA recognition motifs (RRMs) in the N-terminal and four K homology (KH) RNA binding domains in the C-terminal [28]. To elucidate the region of IGF2BP3 binding with circSIPA1L3, we established six FLAG-tagged expression plasmids for IGF2BP3, and the molecular weight and successful expression was confirmed by western blot (Fig. 4d). Then, flag antibody-mediated RIP experiment was conducted, and the results showed that circSIPA1L3 was able to bound with IGF2BP3, especially through RRM1 and RRM2 regions (Fig. 4e). Together, these results suggested that circSIPA1L3 physically interacted with IGF2BP3 in the cytoplasm. The expression of IGF2BP3 was increased in breast cancer tissues compared to normal tissues based on our cohort and The Cancer Genome Atlas (TCGA) dataset (Fig. 4f, Figure S13I), and high IGF2BP3 expression was associated with worse prognosis of breast cancer patients (Figure S13J). Functional experiments showed that IGF2BP3 overexpression promote malignant behaviors of breast cancer cells, and vice versa (Figure S14). Furthermore, rescue assays revealed that IGF2BP3 knockdown compensated for the enhanced malignant behaviors caused by circSIPA1L3 overexpression (Fig. 4g-k, Figure S15A-E). Consistently, circSIPA1L3 knockdown led to attenuated progression and glycolysis which could be rescued by IGF2BP3 overexpression (Figure S15F-I). To further elucidate the significance of circSIPA1L3-IGF2BP3 interaction, we transfected circSIPA1L3-overexpressing breast cancer cells with or without the truncated IGF2BP3 plasmids which including the binding regions between circSIPA1L3 and IGF2BP3 (termed IGF2BP3-△1). The truncated IGF2BP3 retained the combining ability with circSIPA1L3 but losing the ability to regulate RNA stability, which was speculated to inhibit malignant phenotypes of breast cancer cells through competitively binding circSIPA1L3 with wild-type IGF2BP3. As expected, the increased cell proliferation, migration, and invasion after circSIPA1L3 overexpression could be attenuated by transfection with IGF2BP3-△1 plasmids (Figure S16). These results further emphasized the significant role of the increased circSIPA1L3-IGF2BP3 interaction in breast cancer progression.

### CircSIPA1L3 inhibits the ubiquitin/proteasomemediated degradation of IGF2BP3 through serving as a scaffold to enhance IGF2BP3-USP7 interaction

**Fig. 5:**
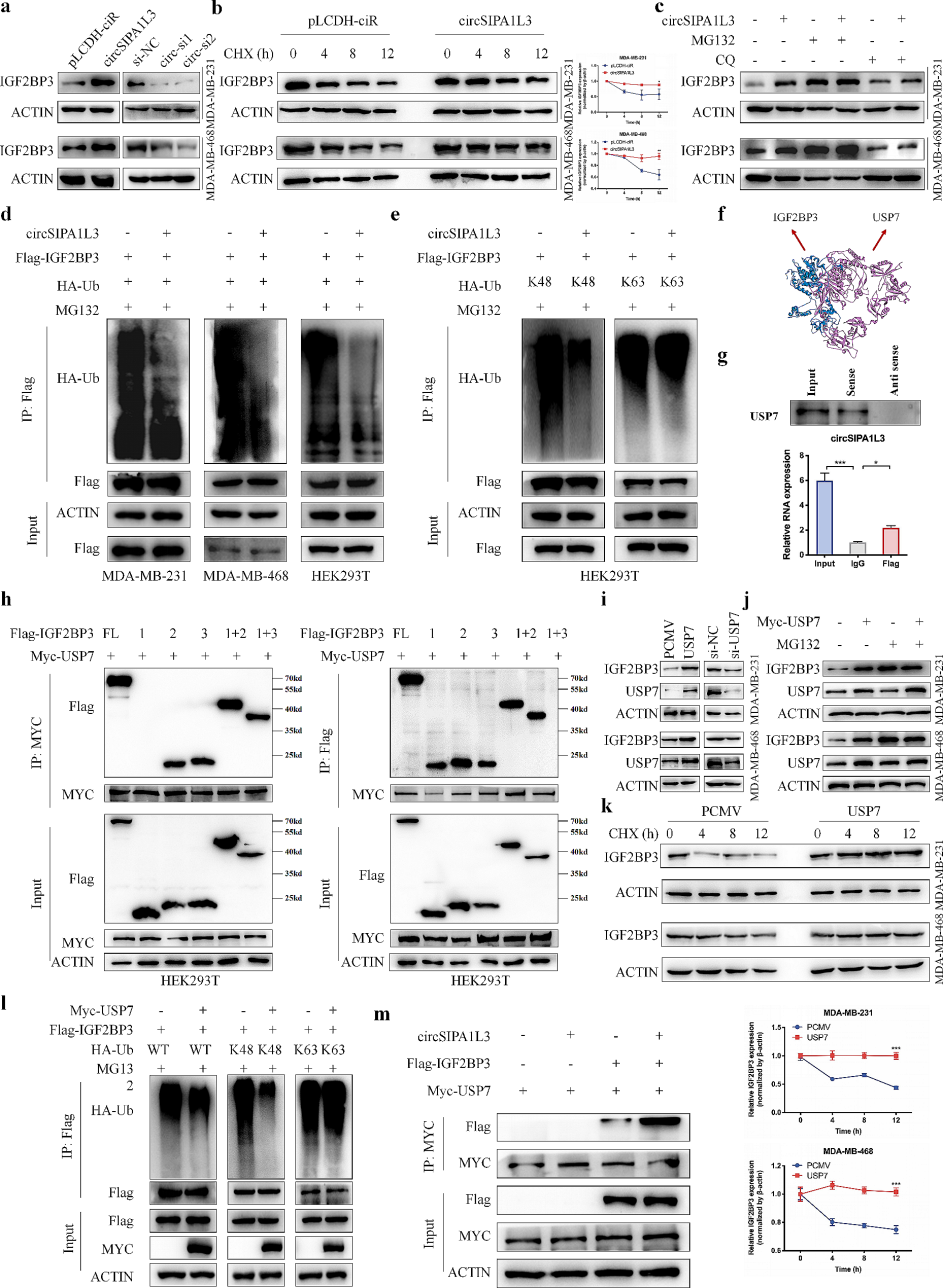
circSIPA1L3 inhibits IGF2BP3 degradation through enhancing the binding between IGF2BP3 and USP7. (**a**) The protein levels of IGF2BP3 were detected by western blot in breast cancer cells after circSIPA1L3 overexpression or knockdown. (**b**) Western blot was used to analyze the protein levels of IGF2BP3 in breast cancer cells transfected with pLCDH-ciR or circSIPA1L3 plasmids, followed by treatment with 100 µg/ml CHX for indicated time. ImageJ software was used to quantify IGF2BP3 expression and β-actin was severed for normalization. (**c**) Following transfection with pLCDH-ciR or circSIPA1L3 plasmids and treatment with MG132 or chloroquine (CQ) for 4 h, breast cancer cell lysates were subjected to western blot. (**d**) Co-IP and western blot assays were used to evaluate the effect of circSIPA1L3 overexpression on the ubiquitination of IGF2BP3 proteins in breast cancer cells and HEK293T cells. (**e**) Co-IP and western blot assays were performed for lysates from HEK293T cells. (**f**) The interaction between IGF2BP3 and USP7 was predicted by ZDOCK server, and the predicted complex structure was visualized using Discovery Studio software. (**g**) The binding between circSIPA1L3 and USP7 was confirmed by RNA pulldown assay and RIP assay. (**h**) HEK293T cells were transfected with Myc-USP7 and the indicated Flag-tagged plasmids, then co-IP was performed to assess the binding region between IGF2BP3 and USP7 using anti-Myc (left) or anti-Flag (right) antibodies. (**i**) Western blot shows the protein levels of IGF2BP3 and USP7 after USP7 overexpression or knockdown in breast cancer cells. (**j**) Breast cancer cells transfected PCMV or USP7 plasmids were treated with or without MG132, and western blot was used to detect the protein levels of IGF2BP3 and USP7. (**k**) Western blot was used to analyze the protein levels of IGF2BP3 in breast cancer cells transfected with PCMV or USP7 plasmids, followed by treatment with 100 µg/ml CHX for indicated time. ImageJ software was used to quantify IGF2BP3 expression and β‐actin was severed for normalization. (**l**) Co-IP and western blot assays were used to evaluate the effect of USP7 overexpression on the ubiquitination of IGF2BP3 proteins. (**m**) HEK-293T cells co-transfected with Flag-IGF2BP3, Myc-USP7, and circSIPA1L3 plasmids as indicated were lysed, immunoprecipitated with anti-MYC antibodies, and subjected to western blot analysis. (*P < 0.05, ***P < 0.001)

Although the mRNA level of IGF2BP3 was not significantly changed after circSIPA1L3 overexpression or knockdown, the IGF2BP3 protein levels were significantly regulated after the alteration of circSIPA1L3 expression (Fig. 5a, Figure S17A-C). Therefore, we speculated that circSIPA1L3 might be involved in post-translational modification of IGF2BP3. Cycloheximide (CHX) treatment revealed that circSIPA1L3 overexpression extended the half-life of IGF2BP3 protein (Fig. 5b), indicating the significant role of circSIPA1L3 in regulating the stability of IGF2BP3 protein. Moreover, the IGF2BP3 expression could be elevated by proteasome inhibitor MG132 (Figure S17D). MG132, but not chloroquine (CQ), was sufficient to eliminate the observed circSIPA1L3-induced IGF2BP3 upregulation (Fig. 5c), revealing that circSIPA1L3 regulated the stability of IGF2BP3 via proteasomal pathway. Given the significant role of ubiquitination modification in mediating protein degradation through proteasomal pathway, we evaluated the ubiquitination level of IGF2BP3 induced by circSIPA1L3. The co-IP assay observed that circSIPA1L3 overexpression significantly decreased the ubiquitination of IGF2BP3 (Fig. 5d, Figure S17E). The level of K48- and K63-linked polyubiquitin were further examined, two major types of polyubiquitin, which associated with degradation and stabilization of substrate proteins respectively [29, 30]. We found that circSIPA1L3 markedly inhibited the K48‐linked polyubiquitination of IGF2BP3, but showed no impact on K63‐linked polyubiquitination of IGF2BP3 (Fig. 5e). Together, these findings provided clear evidence that circSIPA1L3 mediated IGF2BP3 degradation through inhibiting K48-linked polyubiquitination.

Subsequently, we sought to identify the specific enzyme that mediated the regulatory effect of circSIPA1L3 on IGF2BP3 degradation in breast cancer. The co-IP experiments and mass spectrometry were performed in breast cancer cells to detect the potential deubiquitinase for IGF2BP3. After integration with the results of RNA pull-down using circSIPA1L3 probes, the ubiquitin specific peptidase 7 (USP7), a protein that deubiquitinates target proteins, was selected as the candidate (Figure S18A, B). The three-dimensional circSIPA1L3-IGF2BP3 complex was predicted by ZDOCK software and visualized by Discovery Studio (Fig. 5f). Furthermore, the binding between circSIPA1L3 and USP7 was validated by RNA pulldown and RIP assays (Fig. 5g, Figure S18C). To elucidate the role of USP7 in regulating IGF2BP3 stability, co-IP assay was first performed to determine their endogenous and exogenous interaction (Figure S18D, E). IF assay further confirmed that IGF2BP3 colocalized with USP7 in the cytoplasm (Figure S18F). Moreover, co-IP assay using a series of IGF2BP3 truncation mutants indicated that USP7 could bind to the KH domains of IGF2BP3 (Fig. 5h), which is different from the site where circSIPA1L3 interacts with IGF2BP3. We found that USP7 could positively regulated IGF2BP3 expression, and the elevated IGF2BP3 expression induced by USP7 overexpression could be abolished by MG132 treatment (Fig. 5i, j, Figure S18G). In addition, overexpression of USP7 efficiently prolonged the half-life of IGF2BP3 (Fig. 5k). As expected, the ubiquitination level of IGF2BP3, especially K48-linked ubiquitylation, was obviously decreased in cells with USP7 overexpression (Fig. 5l, Figure S18H). The above results indicated that USP7 prevented IGF2BP3 from ubiquitin–proteasome pathway mediated degradation through promoting deubiquitination in breast cancer. Interestingly, low glucose treatment or circSIPA1L3 had on obvious effect on the expression of UPS7 (Figure S19A-C). Therefore, we further investigated the potential role of circSIPA1L3 in mediating the binding ability of USP7 with IGF2BP3. Significantly, IF and co-IP assay determined that the interaction between IGF2BP3 and USP7 was remarkably enhanced by circSIPA1L3 overexpression (Fig. 5m, Figure S19D-F), whereas inhibited after circSIPA1L3 knockdown (Figure S19G). Collectively, these data showed that circSIPA1L3 stabilized IGF2BP3 protein by enhancing the binding between IGF2BP3 and USP7.

### circSIPA1L3-IGF2BP2 facilitates the stability of SLC16A1/RAB11A mRNA levels

**Fig. 6:**
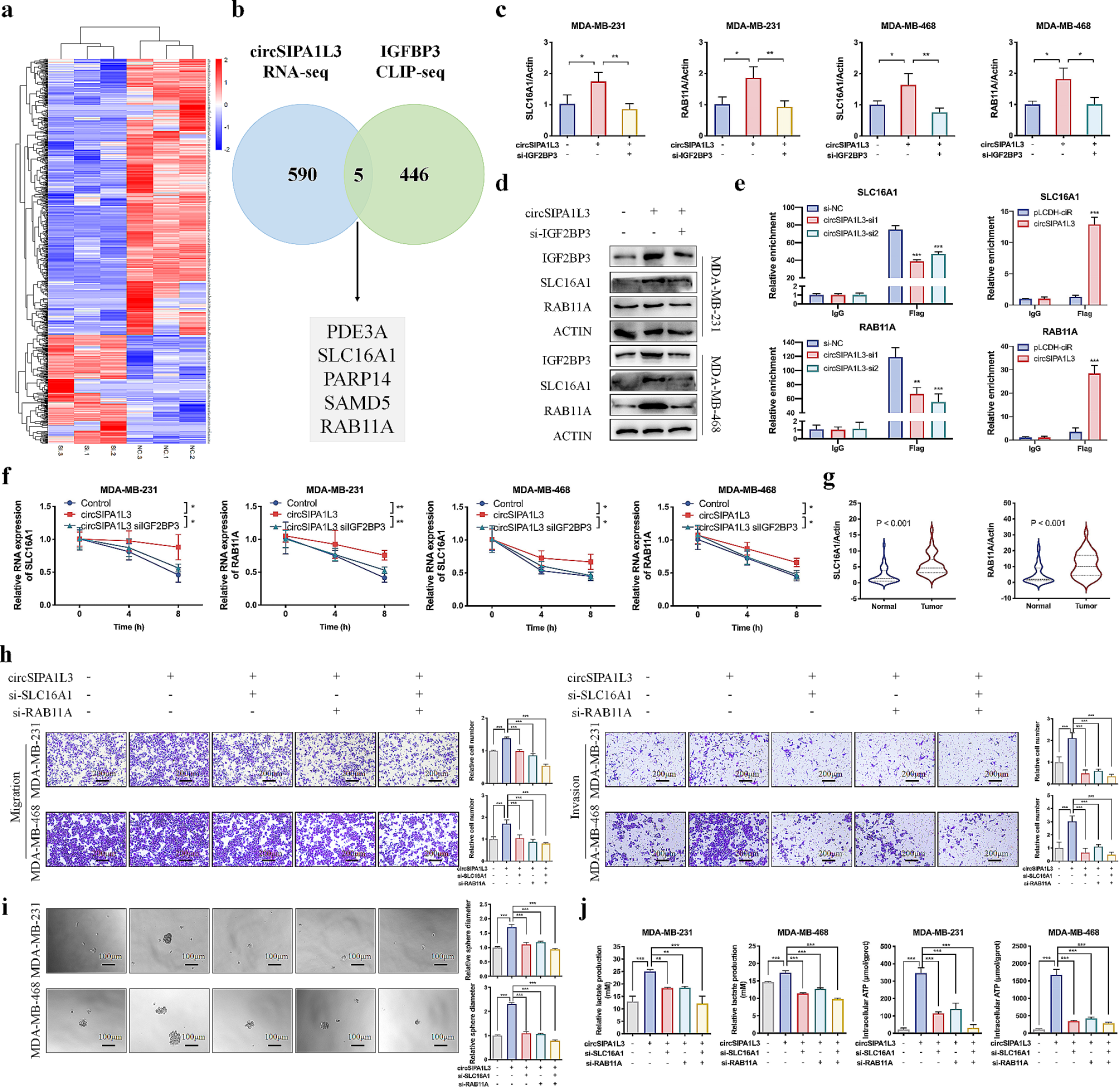
circSIPA1L3/IGF2BP3 enhances the stability of SLC16A1 and RAB11A mRNA levels. (**a**) Heat map shows the differentially expressed genes in MDA-MB-231 cells after circSIPA1L3 knockdown. (**b**) Venn diagram shows the potential target genes of both circSIPA1L3 and IGF2BP3 from RNA-seq data and CLIP-seq data. (**c**, **d**) The qRT-PCR (**c**) and western blot (**d**) shows the RNA and protein levels of SLC16A1 and RAB11A in breast cancer cells co-transfected with circSIPA1L3 overexpressing plasmids and IGF2BP3 siRNA as indicated. (**e**) The relative enrichment of IGF2BP3 in the 3’UTR of SLC16A1 and RAB11A mRNA was detected by RIP assay after circSIPA1L3 knockdown or overexpression. (**f**) The mRNA stability of SLC16A1 and RAB11A was assessed by qRT-PCR in breast cancer cells co-transfected with circSIPA1L3 overexpressing plasmids and IGF2BP3 siRNA as indicated upon ActD treatment. (**g**) The expression of SLC16A1 and RAB11A was detected in normal tissues and breast cancer tissues. (**h**, **i**) Breast cancer cells were transfected with pLCDH-ciR, circSIPA1L3, si-NC, or si-SLC16A1/RAB11A alone or simultaneously, and the abilities of cell migration, invasion, and stemness were assessed by transwell assay (**h**), and tumor sphere formation assay (**i**), respectively. (**j**) *P* < 0.05, **P < 0.01, ***P < 0.001)

Given the involvement of IGF2BP3 in governing mRNA stability through post-transcriptional regulation, we speculated that circSIPA1L3 might modulate mRNA stability through interacting with IGF2BP3 in breast cancer. RNA-seq analysis was performed in circSIPA1L3 knockdown and control cells, and the differentially expressed genes participated in several tumor-related pathways (Figure S20A). Among them, 595 genes were significantly decreased upon circSIPA1L3 downregulation (Fold Change≤-2) (Fig. 6a). Then, we identified 5 potential mRNAs bound by IGF2BP3 through combining the predicted IGF2BP3-binding mRNAs from published CLIP-Seq data (starBase) and the above sequencing results (Fig. 6b). The qRT-PCR and western blot assays further confirmed that SLC16A1 and RAB11A exhibited significant upregulation or downregulation upon alteration of circSIPA1L3/IGF2BP3 (Figure S20B, C), and low glucose treatment also led to elevated expression of SLC16A1 and RAB11A (Figure S20D). The expression of SLC16A1 and RAB11A was increased in MDA-MB-231 cells that treated with exosomes highly expressing circSIPA1L3 compared to the control group (Figure S20E). Consistently, circSIPA1L3-exosomes treated xenograft tumors exhibited significantly higher expression levels of circSIPA1L3, Ki67, IGF2BP3, SLC16A1, and RAB11A compared to control tumors (Figure S20F). In breast cancer tissues, the expression of SLC16A1 and RAB11A was positively correlated with the expression of circSIPA1L3 (Figure S20G). Moreover, the elevated SLC16A1 and RAB11A expression induced by circSIPA1L3 overexpression could be attenuated by IGF2BP3 knockdown (Fig. 6c, d, Figure S20H, I). In consistent with above results, co-transfection with the IGF2BP3-△1 plasmids to inhibit circSIPA1L3-IGF2BP3 interaction could impair the upregulated RNA and protein levels of SLC16A1/RAB11A induced by circSIPA1L3 overexpression (Figure S20J). The predicted results from starBase database also showed the interaction between IGF2BP2 protein and 3’UTRs of SLC16A1/RAB11A mRNAs (Figure S21A, B), indicating the regulatory potential on their mRNA stability. Therefore, SLC16A1 and RAB11A were selected as the potential targets of circSIPA1L3-IGF2BP3 for further investigation. Subsequently, RIP assay revealed significant enrichment of SLC16A1 and RAB11A mRNA in Flag-coprecipitated RNA fragments, which could be obviously diminished after circSIPA1L3 knockdown and enhanced upon circSIPA1L3 overexpression (Fig. 6e, Figure S21C). We further investigated the effect of circSIPA1L3-IGF2BP3 complex on the mRNA stability of SLC16A1/RAB11A. In accordance with our hypothesis, ectopic expression of circSIPA1L3 or IGF2BP3 greatly prolong the half-life of SLC16A1/RAB11A mRNA, whereas inhibition of circSIPA1L3 or IGF2BP3 decrease the mRNA stability of SLC16A1/RAB11A (Figure S21D, E). Significantly, IGF2BP3 knockdown could diminish the circSIPA1L3 overexpression-induced elevation of SLC16A1/RAB11A mRNA stability (Fig. 6f, Figure S21F). The above results together demonstrated that circSIPA1L3 is bound with IGF2BP3 and formed an RNA-protein ternary complex to enhance the mRNA stability of SLC16A1/RAB11A.

Accumulating evidence revealed that SLC16A1 and RAB11A participated in the progression of various cancers, however, their roles in breast cancer have not been elucidated. We found that the expression levels of SLC16A1 and RAB11A were increased in breast cancer tissues compared to adjacent normal breast tissues (Fig. 6g), indicating their involvement in breast cancer progression. Our results indicated that SLC16A1/RAB11A knockdown led to decreased proliferation, spheroid formation and mobility of breast cancer cells (Figure S22A-E). A prominently decrease in glycolysis was also observed in SLC16A1/RAB11A knockdown cells (Figure S22F). We further explore the role of SLC16A1/RAB11A in circSIPA1L3-mediated malignant behaviors. The rescue experiments indicated that SLC16A1 or RAB11A knockdown could attenuate the glycolysis and progression-promoting effect induced by circSIPA1L3 overexpression, and the combined SLC16A1 and RAB11A silencing could further reinforce these suppressive effects (Fig. 6h-j, Figure S22G). These results revealed that the oncogenic role of circSIPA1L3 in breast cancer depended on the interaction with IGF2BP3 and further stabilization of SLC16A1/RAB11A mRNA.

### CircSIPA1L3 acts as a ceRNA to coordinate the expression of SLC16A1/RAB11A

Various studies reported that cytoplasmic circRNAs might function as a miRNA sponge to regulate gene expression [31]. RIP assay using AGO2 antibody observed the binding between circSIPA1L3 and AGO2 (Figure S23A), proposing the possibility that circSIPA1L3 functions as a ceRNA. Thus, we further explored the potential miRNAs that participated in circSIPA1L3-mediated regulation on the RNA expression of SLC16A1 and RAB11A, and miR-665 was identified based on CircInteractome and starBase databases (Figure S23B). Luciferase assay was conducted to further validate this prediction. Transfection with miR-665 mimics dramatically decreased the luciferase activity of plasmids containing wild-type circSIPA1L3 sequence or 3’UTRs of SLC16A1 and RAB11A, but not the mutant plasmids with mutant corresponding binding sites (Figure S23C-E). In addition, there exist a negative regulatory effect between circSIPA1L3 and miR-665 expression (Figure S23F), and circSIPA1L3-overexpressing exosomes could also decrease the expression of miR-665 in breast cancer cells compared with the control group (Figure S23G). Moreover, the miR-665 expression was downregulated after low glucose treatment (Figure S23H). MiR-665 mimics decreased the expression of SLC16A1 and RAB11A, and the elevated expression of SLC16A1 and RAB11A caused by circSIPA1L3 overexpression could be diminished after transfection of miR-665 mimics (Figure S23I-K). These results indicated that circSIPA1L3 could act as a miRNA sponge for miR-665 to modulate the expression of SLC16A1 and RAB11A in breast cancer. Functional studies found that miR-665 overexpression could inhibit progression and glycolysis of breast cancer cells (Figure S24A-C), and the promoting effect of circSIPA1L3 was partially mediated by miR-665 (Figure S24D-F). These data provided novel insights into circSIPA1L3-mediated breast cancer progression.

#### The clinical relevance of circSIPA1L3p in breast cancer

**Fig. 7:**
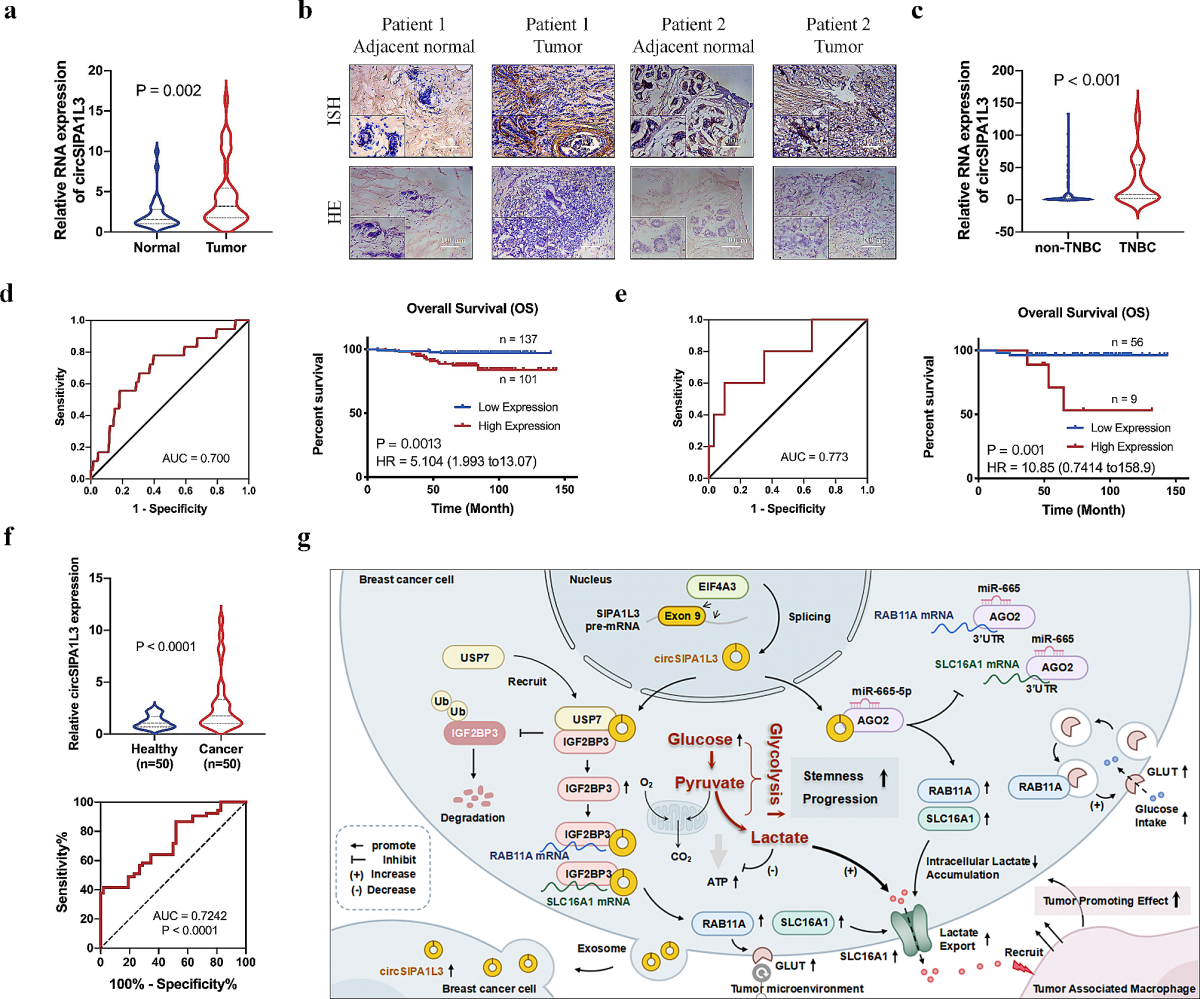
circSIPA1L3 is associated with prognosis of breast cancer patients, and exosome-transmitted circSIPA1L3 promotes progression and glycolysis of breast cancer. (**a**, **b**) The expression of circSIPA1L3 in breast cancer tissues and non-cancerous tissues was evaluated by qRT-PCR (a) and ISH (**b**) assays. (**c**) The circSIPA1L3 expression was evaluated by qRT-PCR in non-TNBC tissues and TNBC tissues. (d, e) ROC analysis was performed to divided breast cancer patients (**d**) or TNBC patients (**e**) into two groups with low and high circSIPA1L3 expression. Kaplan-Meier survival analysis was used to evaluate the correlation between circSIPA1L3 expression and overall survival of breast cancer patients (**d**) or TNBC patients (**e**). (**f**) Violin plot (upper) showing the expression of circSIPA1L3 in the plasma of female breast cancer patients and healthy donors. ROC curve (bottom) was used to assess the diagnostic value of serum circSIPA1L3 for breast cancer. (**g**) Schematic diagram of the mechanism that circSIPA1L3/IGF2BP3 axis promotes breast cancer progression through enhancing glycolysis

Given the oncogenic effect of circSIPA1L3 on cancer progression, we wonder whether circSIP1L3 could serve as a potential biomarker for breast cancer. We found that the circSIPA1L3 expression was significantly elevated in breast cancer tissue compared with normal samples (Fig. 7a, b), and higher circSIPA1L3 expression was associated with ER, PR, and distant metastasis status (Table S1). Consistently, the elevated expression of circSIPA1L3 was detected in TNBC tissues compared to non-TNBC tissues (Fig. 7c). Kaplan–Meier analysis revealed that the higher circSIPA1L3 expression was correlated with poor prognosis of breast cancer patients as well as TNBC patients (Fig. 7d, e). Significantly, univariate and multivariate Cox regression analyses showed that tumor size, distant metastasis status, and circSIPA1L3 expression were independent prognostic factors for the overall survival of breast cancer (Table S2). These above results revealed that circSIPA1L3 could serve as a potential prognostic factor for breast cancer patients. Notably, we also found elevated circSIPA1L3 expression in the serum of breast cancer patients compared with healthy donors, and the ROC (receiver operating characteristic) curve analysis showed an AUC value of 0.72 (Fig. 7f), indicating the high sensitivity and specificity of circSIPA1L3 as a diagnostic biomarker for breast cancer. Together, our results comprehensively demonstrated the essential role and regulatory mechanism of circSIPA1L in breast cancer glycolysis and progression (Fig. 7g).

## Discussion

In recent years, the research concerning metabolic reprogramming have gained widespread attention. Emerging evidence has demonstrated that aerobic glycolysis is the most common feature of cancers, supporting the rapid tumor growth through efficiently generating energy and macromolecules [32]. However, the contribution of glycolysis to breast cancer malignancy is poorly defined. In this study, using RNA-seq analysis, we identified a novel circRNA termed circSIPA1L3, which was induced by glucose limitation and upregulated in breast cancer tissues. Further detection revealed that circSIPA1L3 overexpression was associated with poor prognosis of breast cancer patients, and could promote the malignant behaviors of breast cancer cells through inducing elevated glycolysis.

Aerobic glycolysis is one of the major metabolic features of tumors [33, 34], which could not only produce ATP to supply energy, but also provide substrates or intermediates for the synthesis of biological macromolecules, thus promoting rapid proliferation and metastasis of tumor cells. In addition, increased lactic acid could be produced and released into tumor microenvironment (TME) by tumor cells during glycolysis, resulting in upregulated acidity of the TME and further promoting tumor progression through various mechanisms [35, 36]. Additionally, tumor-associated macrophages (TAMs) could also be recruited by acidic TME to further inhibit anti-tumor immune response and promote tumor progression [37]. Therefore, using aerobic glycolysis as the major energy supply pattern is of great benefit, which could help tumor cells thrive under cellular stress and enhance the metastatic potential. Our study confirmed that circSIPA1L3 showed significant effect on glycolysis of breast cancer cells, and the elevated lactate production induced by circSIPA1L3 overexpression led to enhanced migration ability and tumor-promoting function of TAMs, which could be attenuated by OXM treatment to inhibit lactate secretion. Moreover, the glycolysis inhibitor, 2-DG treatment, could weaken circSIPA1L3 overexpression-mediated enhancement of cancer stemness and progression, further indicating that targeting glycolysis might be a novel direction for cancer treatment.

Based on bioinformatic analysis and experiment verification, we found that EIF4A3, a core member of the exon junction complex [38], could combine with the back-spliced junction site, upstream and downstream flanking sequences of circSIPA1L3 pre-mRNA, promoting its expression and cytoplasmic export. Additionally, the binding of EIF4A3 was also found in circSIPA1L3 sequence, indicating the regulatory potential of circSIPA1L3 on EIF4A3. Nevertheless, the specific regulatory mechanism remains to be further investigated. Further mechanism exploration identified IGF2BP3 as a potential binding partner of circSIPA1L3, which showed significant roles in carcinogenesis, tumor progression, and chemoresistance through regulating the stability, translation, and localization of mRNAs [39]. Previous studies also reported the role of IGF2BP3 in mediating tumor glycolytic metabolism and stemness maintenance [40, 41]. In accordance with these findings, our study certified that IGF2BP3 functioned as an oncogene in breast cancer through promoting glycolysis, and was an indispensable mediator of circSIPA1L3-induced malignant effects. Recent researches reported the regulatory effect of E3 ubiquitin ligase or deubiquitinating enzymes on the stability of IGF2BP3 in various cancers [28, 42, 43], however, the mechanism of IGF2BP3 upregulation in breast cancer remains unclear. In this study, we firstly revealed that deubiquitinating enzymes USP7 could inhibit IGF2BP3 ubiquitination in breast cancer. We wonder whether the expression of USP7 could be regulated by circSIPA1L3, however, no obvious change of USP7 expression was detected, which was consistent with the results obtained under low glucose condition. Therefore, the potential of circSIPA1L3 serving as a scaffold to enhance IGF2BP3-USP7 interaction and further upregulate IGF2BP3 in breast cancer cells was explored. Using several Flag-tagged truncated vectors, we identified that the two RRMs motifs of IGF2BP3 are responsible for recruiting circSIPA1L3, while USP7 could bound to the four KH domains of IGF2BP3. Overall, these findings provided a novel mechanism for IGF2BP3 upregulation in breast cancer, and illustrated that circSIPA1L3 functioned as a scaffold to enhance IGF2BP3/USP7 interaction, ultimately protecting IGF2BP3 from ubiquitin-mediated degradation.

We subsequently explored whether IGF2BP3 could participate in the modulation of glycolysis and progression through enhancing mRNA stabilization. Our results demonstrated that circSIPA1L3 functioned as a scaffold for IGF2BP3 to promote the mRNA stability of downstream targets SLC16A1 and RAB11A. SLC16A1 is a member of monocarboxylate transporters (MCTs) that regulates lactate export and glycolytic flux of cancer cells [44]. Lactate is the major product of glycolysis, which could lead to abnormal expression of metabolic genes and attenuated mitochondria function, further regulating various cancer-related processes [45]. However, the accumulation of lactate could decrease intracellular pH value, leading to cell death. To maintain glycolysis efficiency and avoid cell death, the continuously produced and accumulated lactate need to be rapidly transported into the tumor microenvironment (TME). Previous studies reported that suppression of SLC16A1 was accompanied by a decrease in secreted levels of lactate and ATP production, increased intracellular lactate levels, inhibited cancer growth and metastasis, as well as induce apoptotic cell death [46–48], suggesting that blocking SLC16A1-mediated glycolysis might contribute to improvement of cancer treatment. In addition, RAB11A, the first identified member of small GTPase family, was overexpressed in various cancer tissues and associated with the clinical outcomes of cancer patients [49, 50]. RAB11A is localized in the endocytic recycling endosome, which regulates the recycling and re-usage of various membrane proteins, further promoting malignant progression of cancers [51]. Recently, RAB11A was reported to be correlated with glucose homeostasis [52], which is required for the recycling and trafficking of glucose transporters to the cell surface [53, 54]. Expectedly, our study confirmed the tumor-promoting roles of SLC16A1and RAB11A in breast cancer cells, and the promotion of glycolysis and progression caused by circSIPA1L3 overexpression could be eliminated by SLC16A1 or RAB11A knockdown. Our data also found that circSIPA1L3 has the ability to bind with AGO2, indicating its possibility to act as a miRNA sponge [55]. Given that no effect of circSIPA1L3 was observed on IGF2BP3 RNA expression, we wonder whether circSIPA1L3 could enhance SLC16A1/RAB11A expression through counteracting the inhibitory effect of miRNA. Based on database prediction and experiment verification, we confirmed that miR-665 participated in circSIPA1L3-mediated regulation on the SLC16A1 and RAB11A expression. The functional roles of miR-665 are not consistent in different cancers, which might be attributed to diverse upstream signaling [56, 57]. Our findings reported the tumor suppressive role of miR-665 in breast cancer, which is under rigorous regulation and participates in the induced malignant behaviors of circSIPA1L3. However, the regulatory mechanisms of circRNAs are complex, and one circRNA could participate in tumor progression through multiple mechanisms. There might be other regulatory axes involved in circSIPA1L3-mediated cancer progression, which are needed to be further elucidated in the future studies.

Several studies reported that exosomes, carrying proteins, lipids and nucleic acids, had multiple functions through intercellular communication [58]. We also detected the existence of circSIPA1L3 in isolated exosomes from breast cancer cells. In vitro and in vivo experiments determined that exosomal circSIPA1L3 could be taken up by surrounding cancer cells, thereby enabling them to acquire enhanced glycolysis and progression properties. Additionally, circSIPA1L3 manifested higher expression levels in the plasma of breast cancer patients compared to healthy people, indicating that circSIPA1L3 might also be a promising biomarker for breast cancer diagnosis.

## Conclusions

In summary, we identified a novel exosomal circular RNA, circSIPA1L3, participating in the regulation of glucose metabolism in breast cancer. CircSIPA1L3 could be packaged into exosomes and transmitted to surrounding cancer cells, further promoting malignant progression of breast cancer. The expression of circSIPA1L3, which could be regulated by EIF4A3, was upregulated in breast cancer tissues and associated with poor prognosis of breast cancer patients. Functional experiments confirmed that circSIPA1L3 could effectively promote breast cancer progression through enhancing glycolysis. Mechanistically, we first discovered that circSIPA1L3 stabilized IGF2BP3 through facilitating USP7-mediated deubiquitination, thereby contributing to stabilizing the mRNA levels of SLC16A1 and RAB11A, two significant participants during glucose metabolism. Moreover, circSIPA1L3 could also upregulate the expression of SLC16A1 and RAB11A by sponging miR-665. Our findings provided compelling evidence that circSIPA1L3 was a significant regulator in reprograming glucose metabolism, and could serve as a critical prognostic biomarker and promising therapeutic target for breast cancer.

### Electronic supplementary material

Below is the link to the electronic supplementary material.

Supplementary Material 1

Supplementary Material 2

## Data Availability

The data of this research are available from corresponding author upon reasonable request.

## Abbreviations

2-DG: 2-deoxyglucose
ActD: Actinomycin D
circRNA: Circular RNA
CHX: Cycloheximide
CM: Conditioned medium
CQ: Chloroquine
ECAR: Extracellular acidification rate
FCM: Flow cytometry
FISH: Fluorescence in situ hybridization
HE: Hematoxylin and eosin IF: immunofluorescent
ISH: In situ hybridization
KH: K homology
NTA: Nanoparticle tracker analysis
OS: Overall survivall
OXM: Sodium oxamate
RBPs: RNA-binding proteins
RRMs: RNA recognition motifs
TAM: Tumor associated macrophages
TCGA: The Cancer Genome Atlas
TME: Tumor microenvironment
TNBC: Triple negative breast cancer
USP7: Ubiquitin specific peptidase 7
