# Supplementary material for: Exosomal circSIPA1L3-mediated intercellular communication contributes to glucose metabolic reprogramming and progression of triple negative breast cancer
Source: Mol Cancer. 2024 Jun 8;23:125. doi: 10.1186/s12943-024-02037-4 (PMC11161950; doi:10.1186/s12943-024-02037-4)
Supplement: Supplementary file 1 — Supplementary Material 1 [file 12943_2024_2037_MOESM1_ESM.docx]

**Supplementary Materials**

**Supplementary Materials and Methods**

**Cell culture and transfection**

The breast cancer cells (MDA-MB-231 and MDA-MB-468), HEK293T cells, human umbilical vein endothelial cells (HUVECs), and THP-1 cells were originally obtained from the American Type Culture Collection (ATCC, USA). The cells had been authenticated by short tandem repeat DNA profiling, and tested for negative mycoplasma contamination before experiments. MDA-MB-231, MDA-MB-468, HEK293T, and HUVEC cells were cultured in DMEM (Macgene, China) medium, and THP1 cells were maintained in RPMI-1640 medium. The medium was routinely supplemented with 10% fetal bovine serum (FBS, Gibco, USA) and 1% penicillin/streptomycin, and incubated in a humidified incubator containing 5% CO_2_ atmosphere at 37 °C.

The sequence of exon 9 of SIPA1L3 was amplified by PCR and insert into the pLCDH-ciR vector (Invitrogen, Carlsbad, CA, USA) to generate the circSIPA1L3 overexpression plasmid. The full length of IGF2BP3 and USP7 was cloned into pENTER and pCMV vector respectively to conduct pENTER-IGF2BP3 and pCMV-3×myc-USP7 plasmid. The sequences of Flag label and different partial fragments of IGF2BP3 were cloned into pcDNA3.1 vector to conduct pcDNA3.1-3×Flag-IGF2BP3 fragment plasmids. The cells transiently transfected with control vectors or overexpression vectors were screened by puromycin (2 μg/ml) for 2 months to generate stably transfected cell lines. The siRNA and miRNA mimics were purchased from GenePharma (Shanghai, China), and the corresponding sequences were listed in Table S3. JetPRIME (Polyplus) was used for cell transfection according to the manufacturer’s instructions.

**RNA isolation and quantitative real-time PCR (qRT-PCR)**

Total RNA was isolated from cell lines or tissues using Trizol reagent (Invitrogen, USA). The corresponding complementary DNA (cDNA) was obtained using PrimeScript reverse transcriptase (RT) reagent kit (TaKaRa, Shiga, Japan), while miRNA was synthesized using Mir-X miRNA First-Strand Synthesis Kit (Takara) from total RNA. The circRNA or mRNA expression was quantified by qRT-PCR using Hieff qPCR SYBR Green master mix (Yeasen Biotechnology, China). The relative expression levels were calculated using the 2 ^-ΔΔCT^ method. β-actin was used as the reference for circRNA and mRNA, while U6 was used as the reference for miRNA. The sequences of primers are listed in Table S4.

**Western blot assay**

The proteins were extracted using cell lysis buffer for Western and IP (Beyotime, China) with PMSF and NaF, and quantified by BCA Protein Assay Kit (Millipore, USA). The proteins were isolated by 10% SDS-PAGE and transferred onto 0.22 μm PVDF membranes (Millipore, USA). After blocked by 5% non-fat milk for 1 h, the membranes were incubated with specific primary antibodies overnight at 4°C. After washed by 1×TBST, the membranes were incubated with diluted secondary antibodies for 1 h at room temperature and finally detected by chemiluminescence. The primary and secondary antibodies are listed in Table S5.

**3-(4, 5-Dimethylthiazol-2-yl)-2, 5-diphenyltetrazolium bromide (MTT) assay**

1,500 transfected or treated cells were seeded into 96-well plates. After indicated incubation time, 20 μL MTT (5 mg/ml) was added into each well and incubated for another 4 h. Then, the supernatant was removed carefully and 100 μl DMSO was added into each well for dissolution of crystals. The optical density was obtained using a microplate reader (Bio-Rad, USA) at 490 nm from day 0 to day 5 to produce the proliferation curve after calculation.

**Colony formation assay**

1,000 transfected or treated cells were planted into 6-cm culture dishes, and the culture medium was changed every 3 days. After cultured for appropriate time, the cells were washed by PBS, fixed by methyl alcohol, and stained by 0.5% crystal violet. The colonies were finally photographed and counted.

**EdU incorporation assay**

The EdU incorporation assay kit (RiboBio, China) was used for measurement of cell proliferation. Appropriate amount of transfected or treated cells was planted into 96-well plate to reach a density at 60-70%. Cells were cultured in 50 μM EdU solution for 2 h, and fixed in 4% paraformaldehyde. After treated with 0.5% Triton-X 100, cells were stained with Apollo dye and Hoechst dye successively to observe the proliferative cells and nucleus respectively. Images was captured by the fluorescence microscope (Olympus, Japan).

**Wound healing assay**

Transfected or treated cells was seeded into 24-well plate to reach 90% confluence. A 10 μL sterile tip was used to scratch the cells and produce a wound on the monolayer of cells. PBS was used to wash the remain cells and remove the detached cells. Cells were cultured in serum-free DMEM medium for 48 h. Olympus microscope was used for capturing images at indicated time.

**Transwell assay**

6-8×10^4^ transfected or treated cells resuspended in 200 μL DMEM without FBS were added into the upper chamber, while 700 μL DMEM containing 20% FBS was added into the lower chamber. For transwell invasion assay, matrigel (BD Biosciences, USA) was added to the upper insert of the transwell chamber in advance. After 24-48 h, the penetrated cells were washed with PBS, fixed with methyl alcohol, and stained by 0.5% crystal violet. A light microscope was used for image capture, and the cells adhering to the lower surface were counted.

**Tube formation assay**

The culture medium of transfected cells was changed into serum-free DMEM medium 24 h after transfection, and the supernatant was collected as conditional medium after another 24 h, and subsequently centrifuged at 3000×g for 15 min at 4℃. 5×10^4^ HUVECs were resuspended in indicated conditional medium and planted onto solidified 150 μL matrigel (BD Biosciences, USA) in 48-well plate. After 4-6 h, the formed tube structure was captured by a light microscopy, and the tube length was measured.

**RNA pulldown assay, silver staining, Coomassie Brilliant Blue staining and** **mass spectrometry analysis**

RNA pulldown assay was conducted using a Pierce Magnetic RNA-Protein Pull-down Kit (Thermo Fisher Scientific, USA) to obtain corresponding RBPs according to the manufacturer’s protocol, and the protein samples were further identified by western blot. The Fast Silver Staining Kit (Beyotime, China) and Coomassie Brilliant Blue Fast Staining solution (Solarbio, China) were used to visualize the proteins isolated in SDS-PAGE. The mass spectrometry analysis was performed by Beijing Qinglian Biotech, Co., Ltd.

**RNA immunoprecipitation (RIP) assay**

The interaction between circSIPA1L3 and specific proteins was detected by magnetic RIP RNA-binding protein immunoprecipitation kit (Millipore, USA) according to the manufacturer’s instructions. Briefly, lysed cells in RIP lysis buffer were incubated with magnetic beads conjugated with corresponding primary antibodies at 4 °C overnight. The coprecipitated RNA was washed, purified using RNA extraction reagent, and detected by qRT-PCR.

**Luciferase reporter assay**

The full length of circSIPA1L3 and the sequence of predicted miR-665 binding sites of SLC16A1 3’UTR and RAB11A 3’UTR were amplified by PCR, while their mutant fragments were obtained by overlap extension PCR. The PCR fragments were inserted into pmirGLO vectors (Invitrogen, USA) respectively to construct luciferase reporter plasmids. HEK293T cells were co-transfected with the wild type or mutant luciferase reporter plasmids and miR-665 mimics. The luciferase activity was measured by the Dual-Luciferase® Reporter Assay System (Promega, USA). Renilla luciferase activity was used as a reference to calculate the relative luciferase activity.

**Immunohistochemistry (IHC)**

The paraffin-embedded tumor samples obtained from patients or mice were sliced into 4 μm sections, deparaffinized in xylene, rehydrated using gradient alcohol. After antigen retrieval and endogenous peroxidase block the slices were incubated with specific primary antibodies overnight at 4 °C, followed by incubation with HRP-conjugated secondary antibodies. The sections were visualized by DAB solution, followed by counterstaining with hematoxylin. Images were captured by a light microscopy (Olympus). The antibodies were listed in Table S5.
