# Supplementary material for: Exosomal circSIPA1L3-mediated intercellular communication contributes to glucose metabolic reprogramming and progression of triple negative breast cancer
Source: Mol Cancer. 2024 Jun 8;23:125. doi: 10.1186/s12943-024-02037-4 (PMC11161950; doi:10.1186/s12943-024-02037-4)
Supplement: Supplementary file 2 — Supplementary Material 2 [file 12943_2024_2037_MOESM2_ESM.pdf]

Supplementary Figures

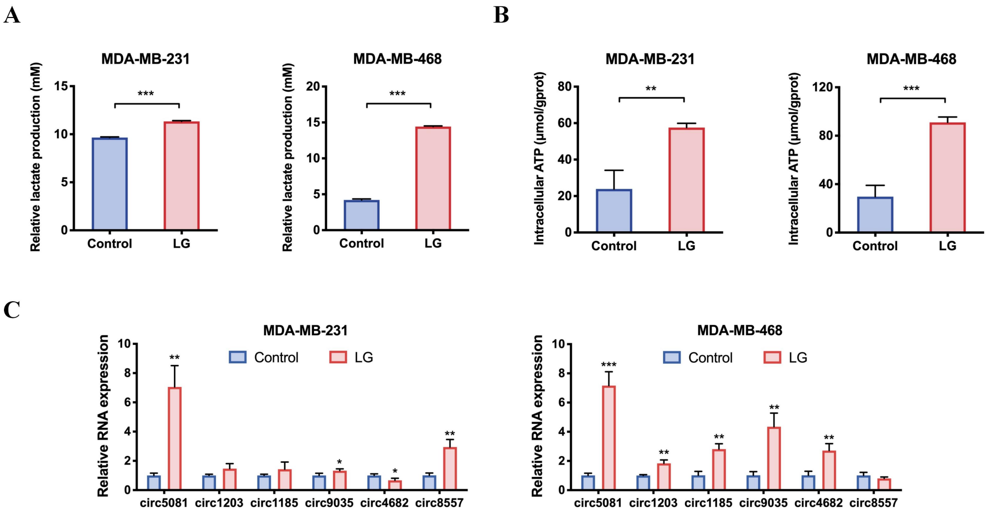

Supplementary Figure S1. Low glucose treatment induced upregulated glycolysis in breast cancer cells. A and B, The lactate production (A) and ATP levels (B) were evaluated in breast cancer cells after low glucose treatment. **C,** The expression of candidate circRNAs were analyzed in breast cancer cells after low glucose treatment. (*P<0.05, **P<0.01, ***P<0.001)

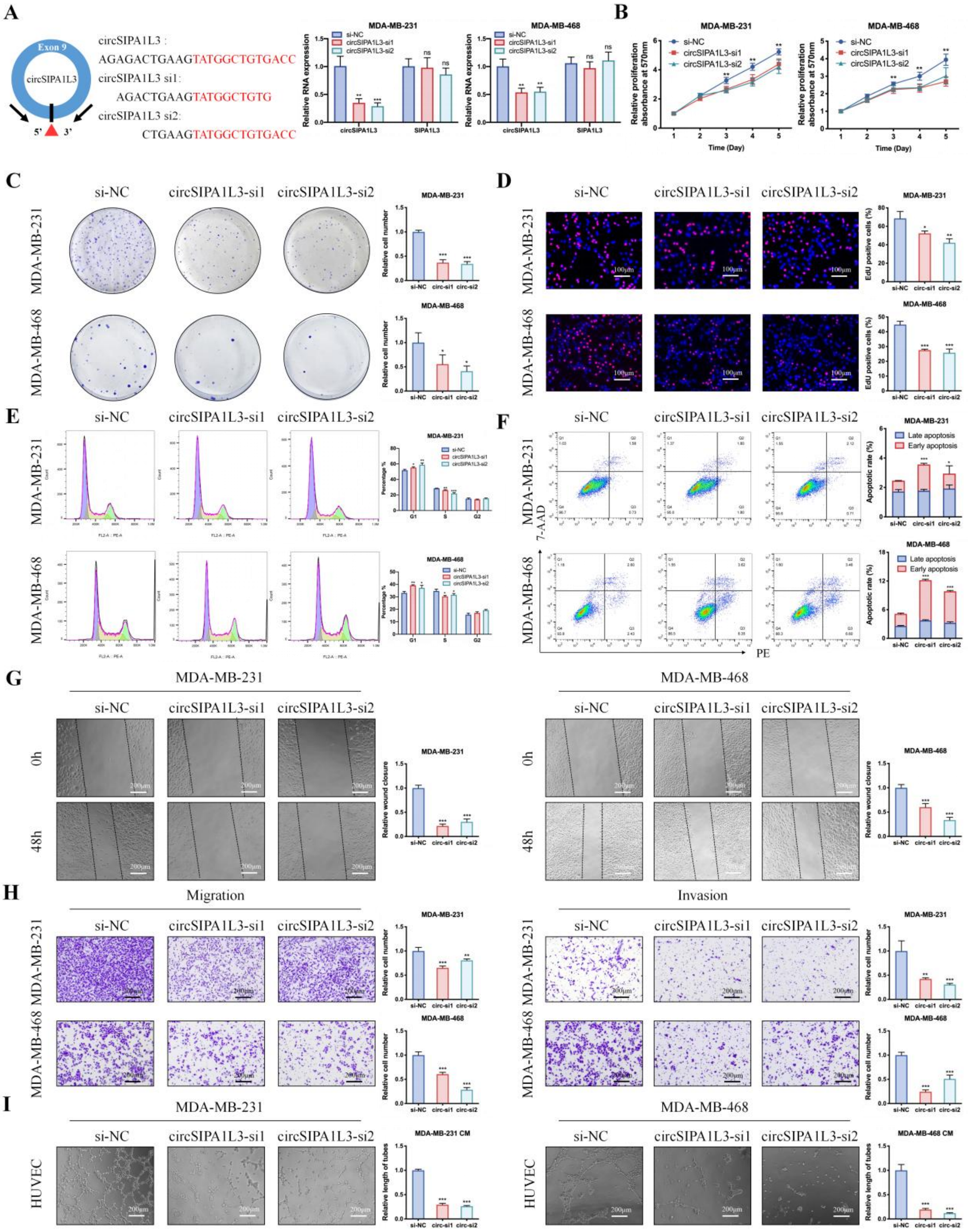
A
Exon 9
circSIPA1L3
5'
3'
circSIPA1L3 :
AGAGACTGAAGTATGGCTGTGACC
circSIPA1L3 si1:
AGACTGAAGTATGGCTGTG
circSIPA1L3 si2:
CTGAAGTATGGCTGTGACC
MDA-MB-231
MDA-MB-468
si-NC
circSIPA1L3-si1
circSIPA1L3-si2
Relative RNA expression
circSIPA1L3
SIPA1L3
ns
B
Relative proliferation absorbance at 570nm
Time (Day)
C
Relative cell number
circ-si1
circ-si2
D
EdU positive cells (%)
100μm
E
Percentage %
G1
S
G2
F
7-AAD
PE
Late apoptosis
Early apoptosis
Apoptotic rate (%)
G
0h
48h
200μm
Relative wound closure
H
Migration
Invasion
I
HUVEC
MDA-MB-231 CM
MDA-MB-468 CM
Relative length of tubes

Supplementary Figure S2. circSIPA1L3 knockdown inhibits proliferation, migration, and invasion of breast cancer cells. A, Schematic illustration shows the target sequences of the siRNAs specific to the back-splicing junction of circSIPA1L3 (left). The efficiency of circSIPA1L3 knockdown in breast cancer cells was verified by qRT-PCR (right). **B-D,** The MTT (B), colony formation (C), and EdU (D) assays were performed to detect the effect of circSIPA1L3 knockdown on proliferation of breast cancer cells. **E and F,** Flow cytometric assays were used to examine the cell cycle (E) and cell apoptosis (F) after circSIPA1L3 knockdown. **G,** Wound healing assay detected migration of circSIPA1L3-knockdown and control breast cancer cells. **H,** Transwell assay evaluated migration and invasion abilities of circSIPA1L3-knockdown and control breast cancer cells. **I** Tube formation assay was performed to evaluate the effect of circSIPA1L3 knockdown on angiogenesis ability of HUVECs. (ns, $P > 0.05$, *$P < 0.05$, **$P < 0.01$, ***$P < 0.001$)

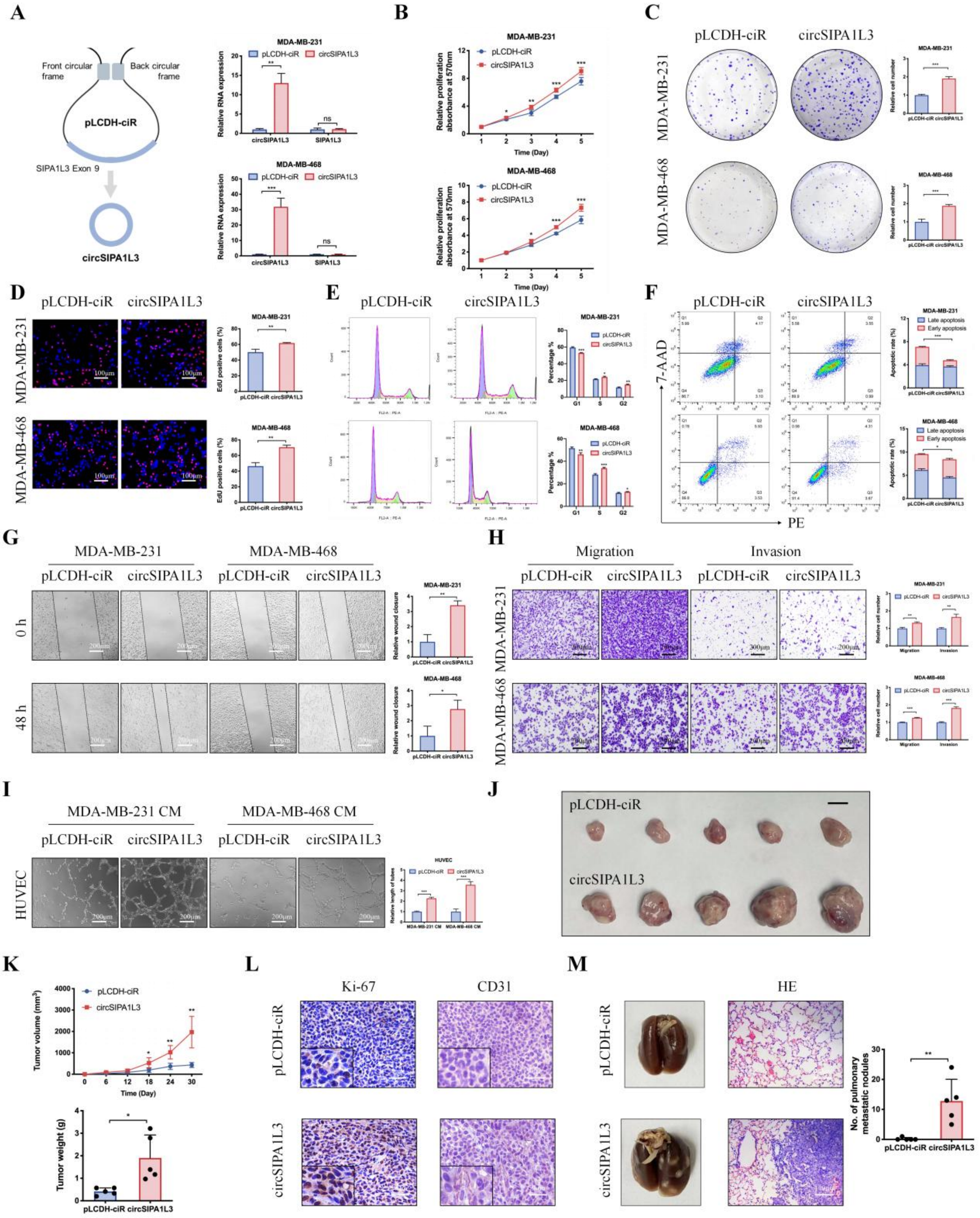

A
Front circular frame
Back circular frame
pLCDH-ciR
SIPA1L3 Exon 9
circSIPA1L3
MDA-MB-231
MDA-MB-468
B
C
pLCDH-ciR
circSIPA1L3
D
E
F
7-AAD
PE
G
0 h
48 h
H
Migration
Invasion
I
MDA-MB-231 CM
MDA-MB-468 CM
HUVEC
J
K
L
Ki-67
CD31
M
HE

Supplementary Figure S3. circSIPA1L3 overexpression promotes breast cancer cell proliferation, migration, and invasion both in vitro and in vivo. A, Schematic illustration shows the formation of circSIPA1L3 overexpression plasmid (left). The efficiency of circSIPA1L3 overexpression was verified by qRT-PCR (right). **B-D,** The MTT (B), colony formation (C), and EdU (D) assays were used to analyze cell viability after circSIPA1L3 overexpression. **E and F,** Flow cytometric assays were performed to detect the effect of circSIPA1L3 overexpression on cell cycle (E) and apoptosis (F). **G,** Wound healing assay was used to detect the effect of circSIPA1L3 overexpression on migration of indicated cells. **H,** Transwell assay measured migration and invasion abilities of circSIPA1L3-overexpressing and control breast cancer cells. **I,** Tube formation assay was performed to evaluate the angiogenesis-promoting effect of circSIPA1L3 overexpression on HUVECs. **J,** A subcutaneous xenograft tumor model was established by injection of MDA-MB-231 cells stably transfected with circSIPA1L3 overexpression or control plasmids into nude mice. The images of dissected xenograft tumors in each group. **K,** The tumor volume and tumor weight were recorded. **L,** Representative images of IHC staining of Ki-67 and CD31 in tumor tissues from different groups. **M,** The lung metastatic mouse models were constructed through tail vein injection of circSIPA1L3 or empty vectors stably transfected MDA-MB-231 cells. The representative images of pulmonary metastasis (left) and HE staining of metastatic nodules (middle). The number of pulmonary metastasis nodules in indicated groups (right). (ns, $P > 0.05$, $^{*}P < 0.05$, $^{**}P < 0.01$, $^{***}P < 0.001$)

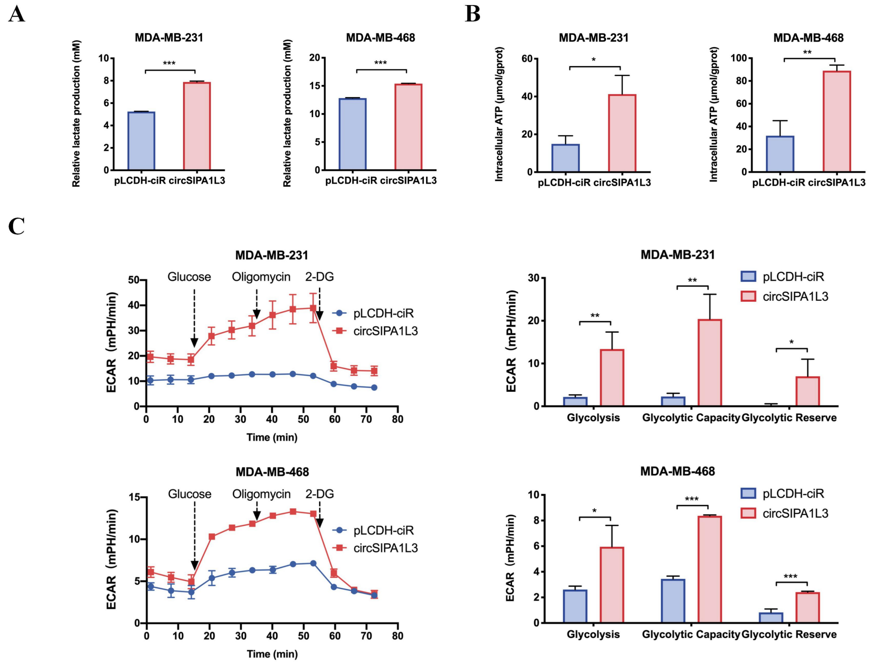

Supplementary Figure S4. circSIPA1L3 overexpression promotes glycolysis of breast cancer cells. A and B, The lactate production (A) and ATP levels (B) were evaluated in breast cancer cells after circSIPA1L3 overexpression. **C,** The extracellular acidification rate (ECAR) was detected using the "Seahorse analyzer" after circSIPA1L3 overexpression in breast cancer cells. The glycolysis, glycolytic capacity, and glycolytic reserve of indicated cells were calculated. (*P<0.05, **P<0.01, ***P<0.001)

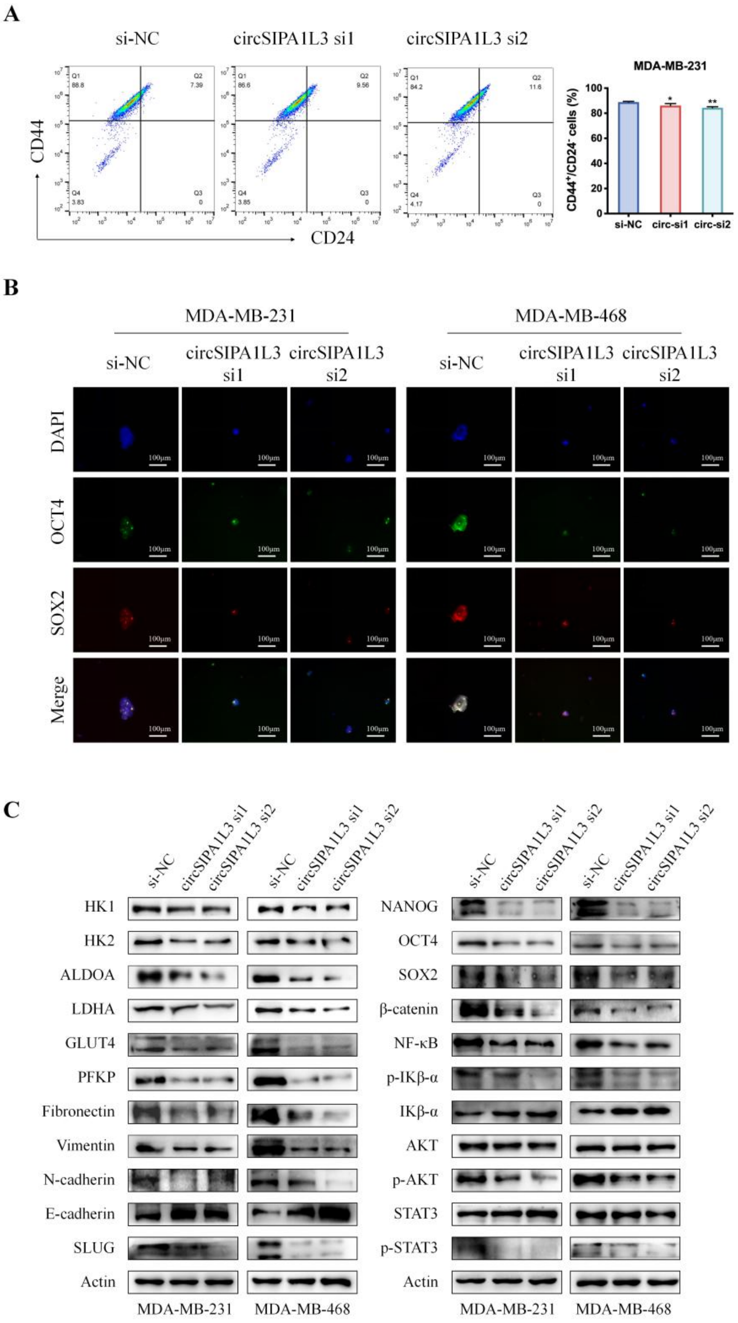

Supplementary Figure S5. circSIPA1L3 knockdown inhibits stemness and glycolysis of breast cancer cells. A, Flow cytometric assays was performed to detect the percentage of $CD44^{+}CD24^{-}$ phenotype in MDA-MB-231 cells transfected with NC or circSIPA1L3 siRNAs. **B,** IF staining assay was used to assess the expression of stemness markers. **C,** The expression of glycolysis-, stemness-, and metastasis-related markers was detected by western blot. (*P<0.05, **P<0.01)

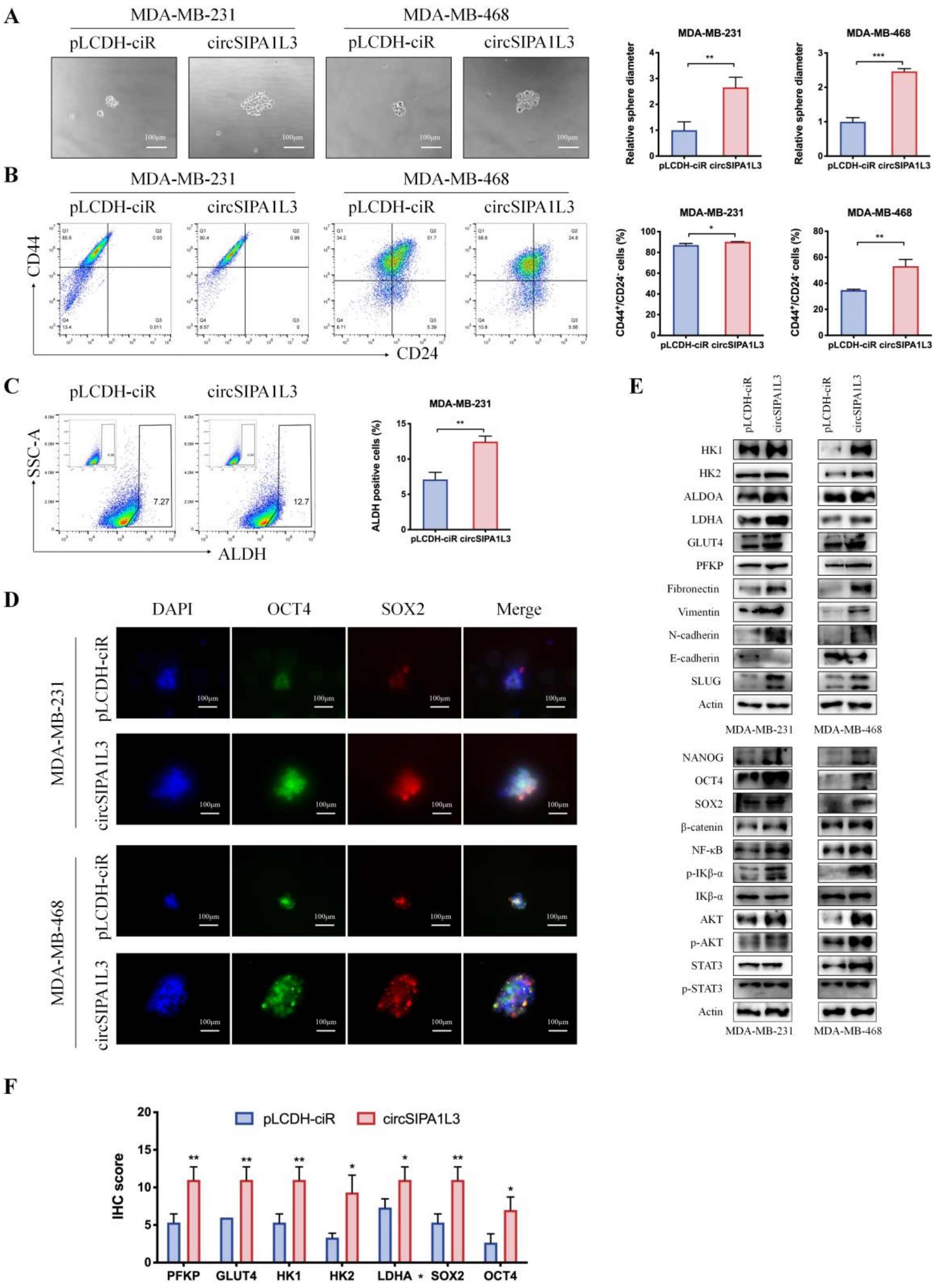

A
MDA-MB-231
MDA-MB-468
pLCDH-ciR
circSIPA1L3
Relative sphere diameter
B
CD44
CD24
CD44+/CD24- cells (%)
C
SSC-A
ALDH
ALDH positive cells (%)
D
DAPI
OCT4
SOX2
Merge
E
HK1
HK2
ALDOA
LDHA
GLUT4
PFKP
Fibronectin
Vimentin
N-cadherin
E-cadherin
SLUG
Actin
NANOG
β-catenin
NF-κB
p-IKβ-α
IKβ-α
AKT
p-AKT
STAT3
p-STAT3
F
IHC score

Supplementary Figure S6. circSIPA1L3 overexpression promotes stemness and glycolysis of breast cancer cells. A, Tumor sphere formation capacity was measured to evaluate the effect of circSIPA1L3 overexpression on stemness of breast cancer cells. **B and C,** Flow cytometric assays was used to detect the percentage of $CD44^{+}CD24^{-}$ phenotype (B) or ALDH positive phenotype (C) in breast cancer cells after circSIPA1L3 overexpression. **D,** IF staining assay was used to assess the expression of stemness markers in control or circSIPA1L3-overexpressing breast cancer cells. **E,** The expression of glycolysis-, stemness-, and metastasis-related markers was detected by western blot. **F**, Quantitative analysis on the indicated factors in IHC by H-score. (*P<0.05, **P<0.01, ***P<0.001)

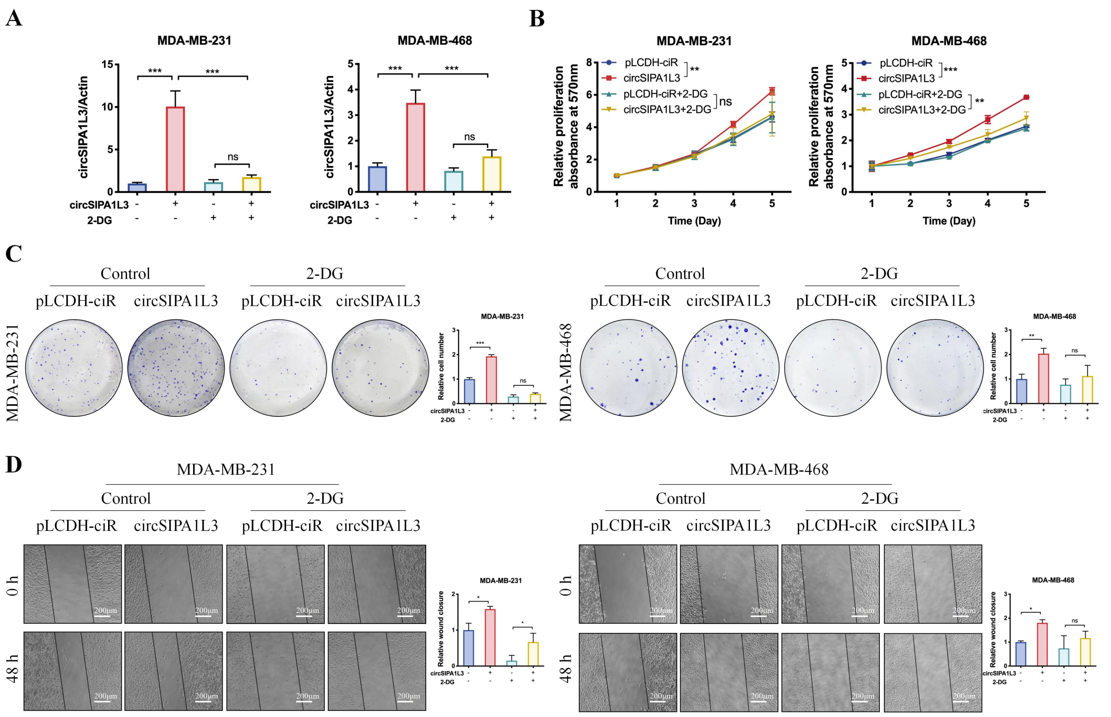

Supplementary Figure S7. circSIPA1L3 promotes breast cancer progression through enhancing glycolysis. A-D, Breast cancer cells were transfected with pLCDH-ciR or circSIPA1L3 overexpressing plasmids, and further treated with or without 2-DG. The expression of circSIPA1L3 in indicated breast cancer cells was detected by qRT-PCR. The cell proliferation was assessed by MTT (B) and colony formation (C) assays. The migration and invasion abilities of indicated breast cancer cells were analyzed by wound healing assay (D). (ns, P > 0.05, *P < 0.05, **P < 0.01, ***P<0.001)

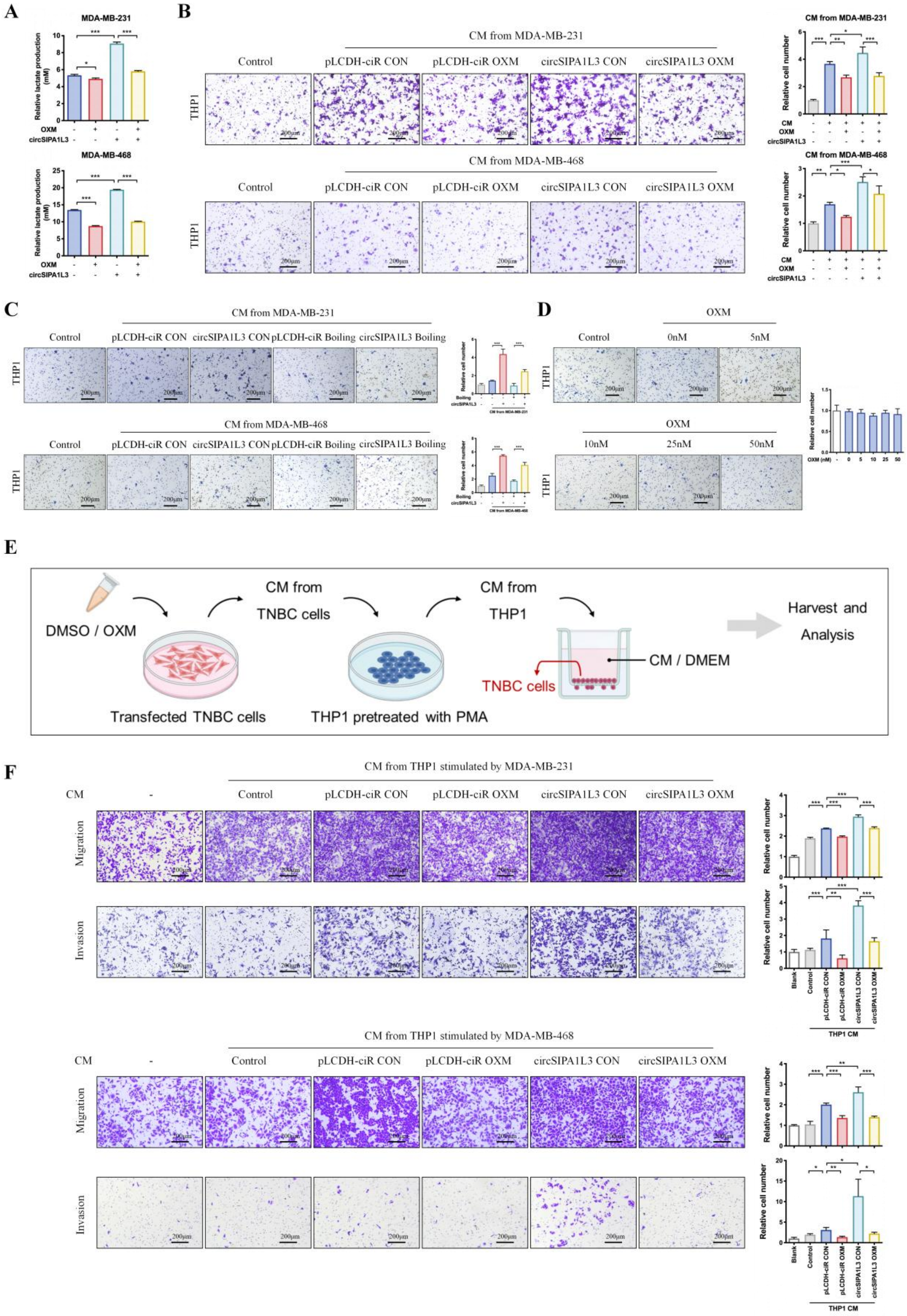

A
MDA-MB-231
Relative lactate production (mM)
OXM
circSIPA1L3
MDA-MB-468
B
CM from MDA-MB-231
Control
pLCDH-ciR CON
pLCDH-ciR OXM
circSIPA1L3 CON
circSIPA1L3 OXM
THP1
200μm
CM from MDA-MB-468
Relative cell number
CM
C
pLCDH-ciR Boiling
circSIPA1L3 Boiling
Boiling
D
OXM
0nM
5nM
10nM
25nM
50nM
OXM (nM)
E
DMSO / OXM
CM from TNBC cells
CM from THP1
Harvest and Analysis
CM / DMEM
TNBC cells
Transfected TNBC cells
THP1 pretreated with PMA
F
CM from THP1 stimulated by MDA-MB-231
Migration
Invasion
Blank
THP1 CM
CM from THP1 stimulated by MDA-MB-468

Supplementary Figure S8. circSIPA1L3 overexpression-induced lactate production contributes to migration and tumor-promoting function of TAMs. A, The lactate concentration in CM from pLCDH-ciR or circSIPA1L3-overexpressing vectors transfected breast cancer cells treated with or without OXM. **B,** The migration ability of TAMs treated with CM from pLCDH-ciR or circSIPA1L3-overexpressing vectors transfected breast cancer cells in the presence of OXM. **C**, The migration ability of TAMs treated with breast cancer cells derived CM with or without boiling. **D**, The migration ability of TAMs treated with OXM in gradient concentration. **E,** Schematic diagram showing the experiment protocol of evaluating biological function of TAMs. **F,** Transwell assay was used to evaluate the migration and invasion promoting abilities of TAMs after treated with indicated CMs from breast cancer cells. (*P<0.05, **P<0.01, ***P<0.001)

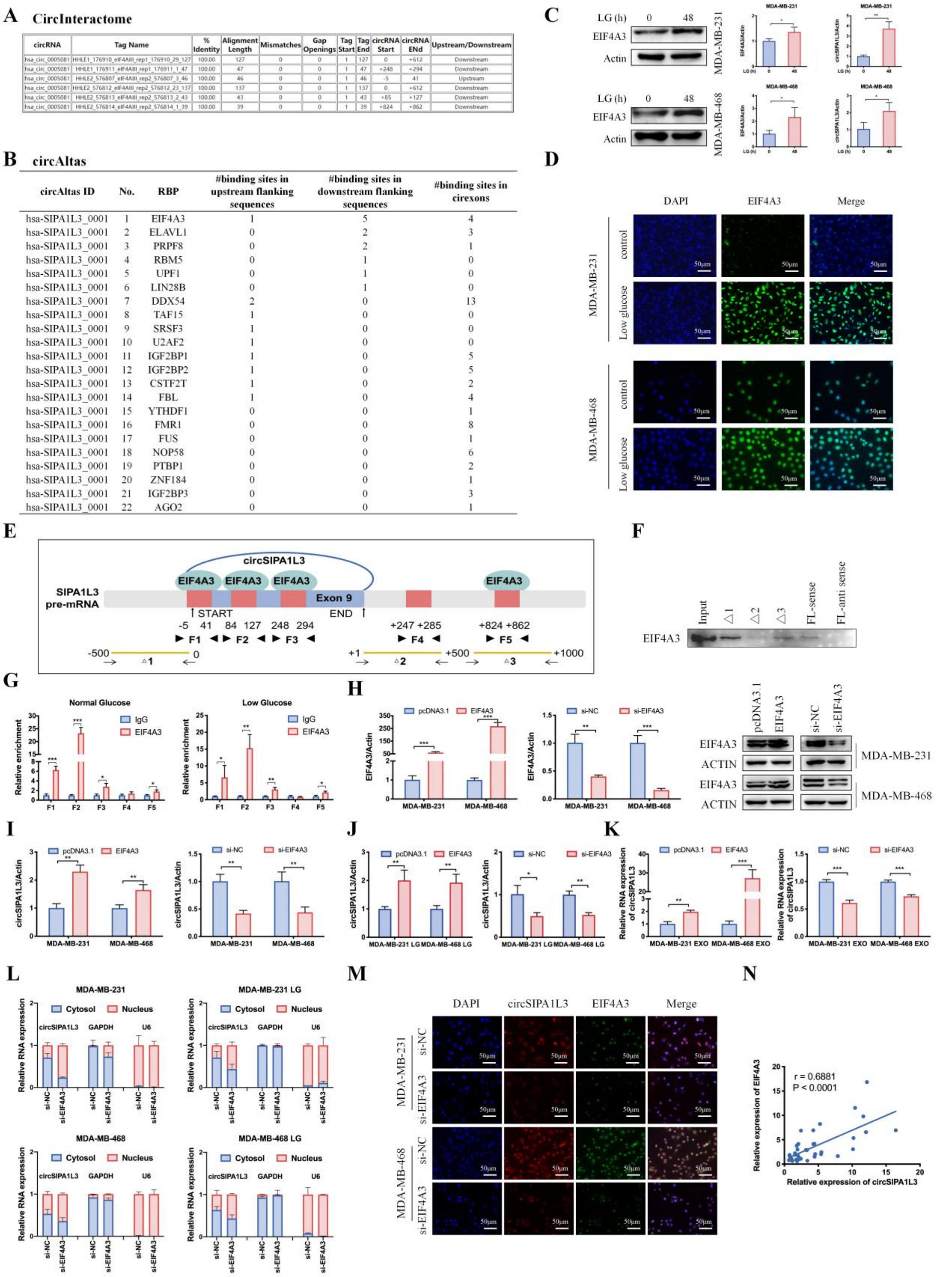

A CircInteractome

circRNA	Tag Name	% Identity	Alignment Length	Mismatches	Gap Openings	Tag Start	Tag End	circRNA Start	circRNA ENd	Upstream/Downstream
hsa_circ_0005081	HHLE1_176910_eIF4AIII_rep1_176910_29_127	100.00	127	0	0	1	127	0	+612	Downstream
hsa_circ_0005081	HHLE1_176911_eIF4AIII_rep1_176911_1_47	100.00	47	0	0	1	47	+248	+294	Downstream
hsa_circ_0005081	HHLE2_576807_eIF4AIII_rep2_576807_3_46	100.00	46	0	0	1	46	-5	41	Upstream
hsa_circ_0005081	HHLE2_576812_eIF4AIII_rep2_576812_23_137	100.00	137	0	0	1	137	0	+612	Downstream
hsa_circ_0005081	HHLE2_576813_eIF4AIII_rep2_576813_2_43	100.00	43	0	0	1	43	+85	+127	Downstream
hsa_circ_0005081	HHLE2_576814_eIF4AIII_rep2_576814_1_39	100.00	39	0	0	1	39	+824	+862	Downstream

B circAltas

circAltas ID	No.	RBP	#binding sites in upstream flanking sequences	#binding sites in downstream flanking sequences	#binding sites in cirexons
hsa-SIPA1L3_0001	1	EIF4A3	1	5	4
hsa-SIPA1L3_0001	2	ELAVL1	0	2	3
hsa-SIPA1L3_0001	3	PRPF8	0	2	1
hsa-SIPA1L3_0001	4	RBM5	0	1	0
hsa-SIPA1L3_0001	5	UPF1	0	1	0
hsa-SIPA1L3_0001	6	LIN28B	0	1	0
hsa-SIPA1L3_0001	7	DDX54	2	0	13
hsa-SIPA1L3_0001	8	TAF15	1	0	0
hsa-SIPA1L3_0001	9	SRSF3	1	0	0
hsa-SIPA1L3_0001	10	U2AF2	1	0	0
hsa-SIPA1L3_0001	11	IGF2BP1	1	0	5
hsa-SIPA1L3_0001	12	IGF2BP2	1	0	5
hsa-SIPA1L3_0001	13	CSTF2T	1	0	2
hsa-SIPA1L3_0001	14	FBL	1	0	4
hsa-SIPA1L3_0001	15	YTHDF1	0	0	1
hsa-SIPA1L3_0001	16	FMR1	0	0	8
hsa-SIPA1L3_0001	17	FUS	0	0	1
hsa-SIPA1L3_0001	18	NOP58	0	0	6
hsa-SIPA1L3_0001	19	PTBP1	0	0	2
hsa-SIPA1L3_0001	20	ZNF184	0	0	1
hsa-SIPA1L3_0001	21	IGF2BP3	0	0	3
hsa-SIPA1L3_0001	22	AGO2	0	0	1

Supplementary Figure S9. The RNA binding protein EIF4A3 regulates the biogenesis and cytoplasmic export of circSIPA1L3. A and B, The potential RNA-binding proteins of circSIPA1L3 flanking sequences were predicted by

CircInteractome and circAltas databases. **C,** Breast cancer cells were treated with or without low glucose for 48 h. Western blot was used to detect the protein levels of EIF4A3, and the mRNA levels of EIF4A3 and circSIPA1L3 were assessed by qRT-PCR. **D,** IF assay was performed to evaluate the expression of EIF4A3 in breast cancer cells under low glucose treatment for 0 and 48 h. **E,** Schematic diagram shows the putative binding sites of EIF4A3in the upstream and downstream region of SIPA1L3 pre-mRNA transcript, which were predicted using CircInteractome database. **F,** RNA pulldown and western blot assays were conducted to illustrate the possible binding proteins in the circSIPA1L3 flanking sequences. **G,** RIP and qRT-PCR assays were performed using EIF4A3 antibodies or IgG to verify the putative binding site of SIPA1L3 pre-mRNA with EIF4A3 in normal (left) or low glucose condition (right). **H,** The qRT-PCR and western blot assays show the expression of EIF4A3 in breast cancer cells after EIF4A3 overexpression or knockdown. **I-J,** The RNA levels of circSIPA1L3 was detected after EIF4A3 overexpression or knockdown in normal (I) or low glucose condition (**J**). **K,** The effect of overexpression or knockdown of EIF4A3 on the level of circSIPA1L3 in exosomes from MDA-MB-231 and MDA-MB-468. **L**, The Nuclear-cytoplasmic fractionation assay was used to evaluate the cytoplasmic export of circSIPA1L3 in breast cancer cells upon EIF4A3 knockdown. **M**, The expression and subcellular localization of EIF4A3 and circSIPA1L3 in breast cancer cells were detected by FISH and IF assays. Nuclei were stained with DAPI. **N,** The correlation between circSIPA1L3 and EIF4A3 expression in breast cancer tissues. (*P<0.05, **P<0.01, ***P<0.001)

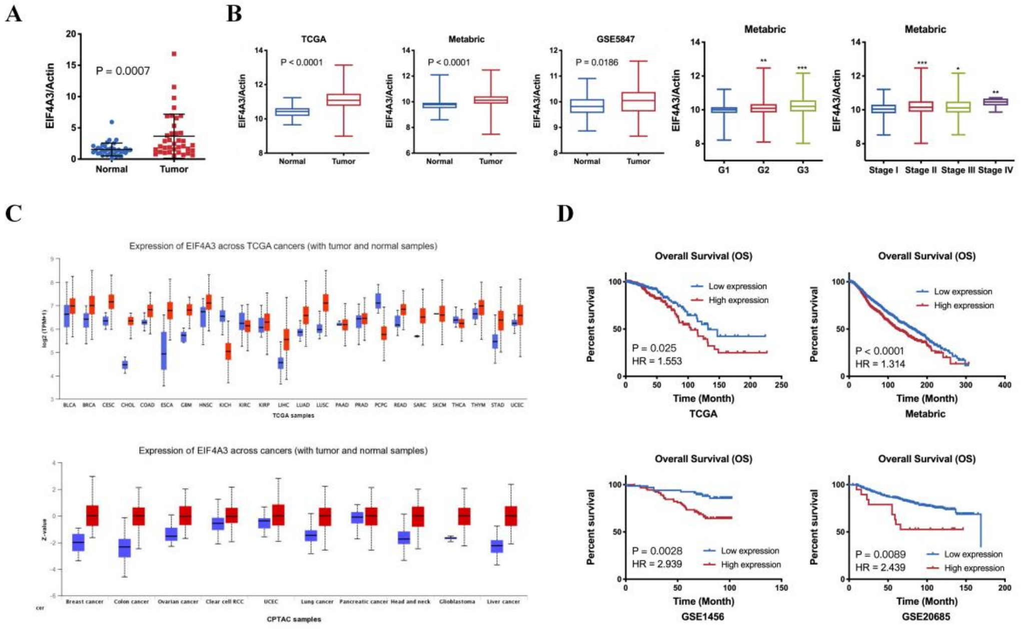

Supplementary Figure S10. The cancer-associated clinical correlation of EIF4A3.

A, The relative mRNA level of EIF4A3 in breast cancer tissues and normal tissues was analyzed by qRT-PCR. **B,** The mRNA expression of EIF4A3 in normal tissues and breast cancer tissues were analyzed using different databases. **C,** The mRNA (upper) and protein (bottom) expression levels of EIF4A3 in various cancers were evaluated using UALCAN database (https://ualcan.path.uab.edu/index.html). **D,** Survival analysis showed the association between EIF4A3 expression and overall survival of breast cancer patients in different databases. (*P<0.05, **P<0.01, ***P<0.001)

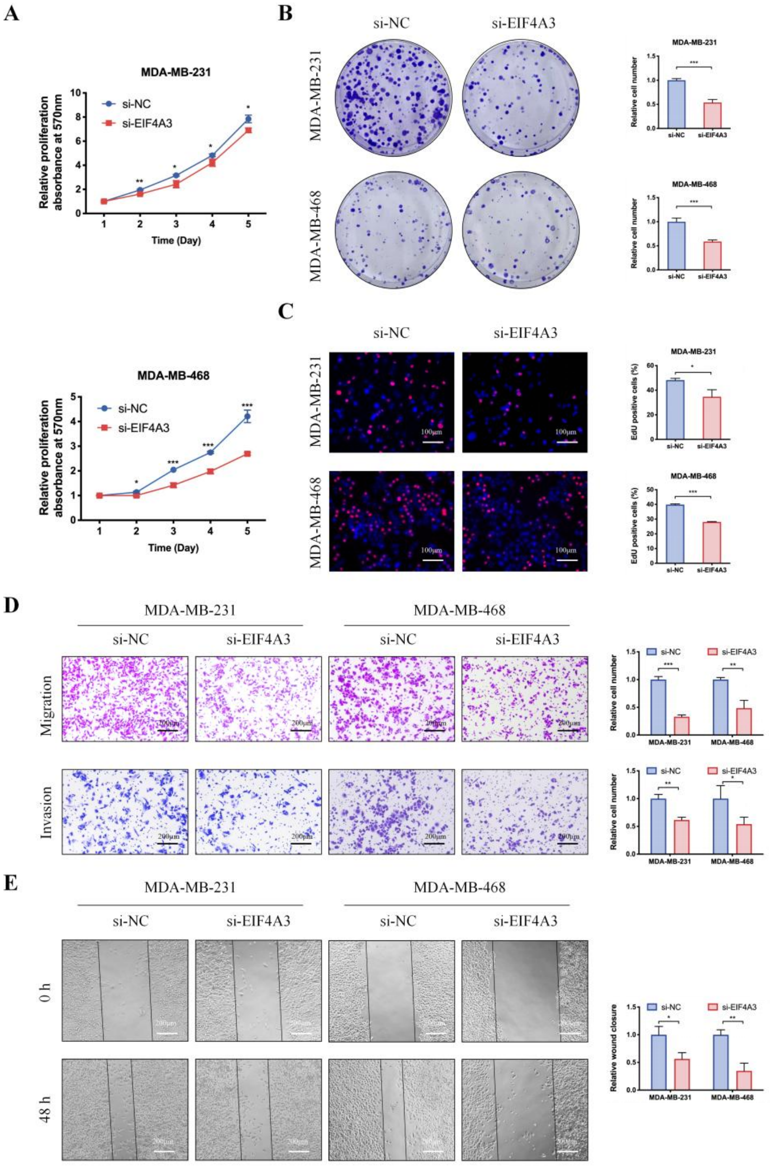

Supplementary Figure S11. EIF4A3 promotes proliferation, migration, and invasion of breast cancer cells. A-C, The MTT (A), colony formation (B), and EdU (C) assays shows the proliferative ability of breast cancer cells transfected with si-NC or si-EIF4A3. **D,** Transwell assay evaluates the migration and invasion ability of breast cancer cells transfected with si-NC or si-EIF4A3. **E,** Wound healing assay shows the effect of EIF4A3 knockdown on migration ability of breast cancer cells. (*P<0.05, **P<0.01, ***P<0.001)

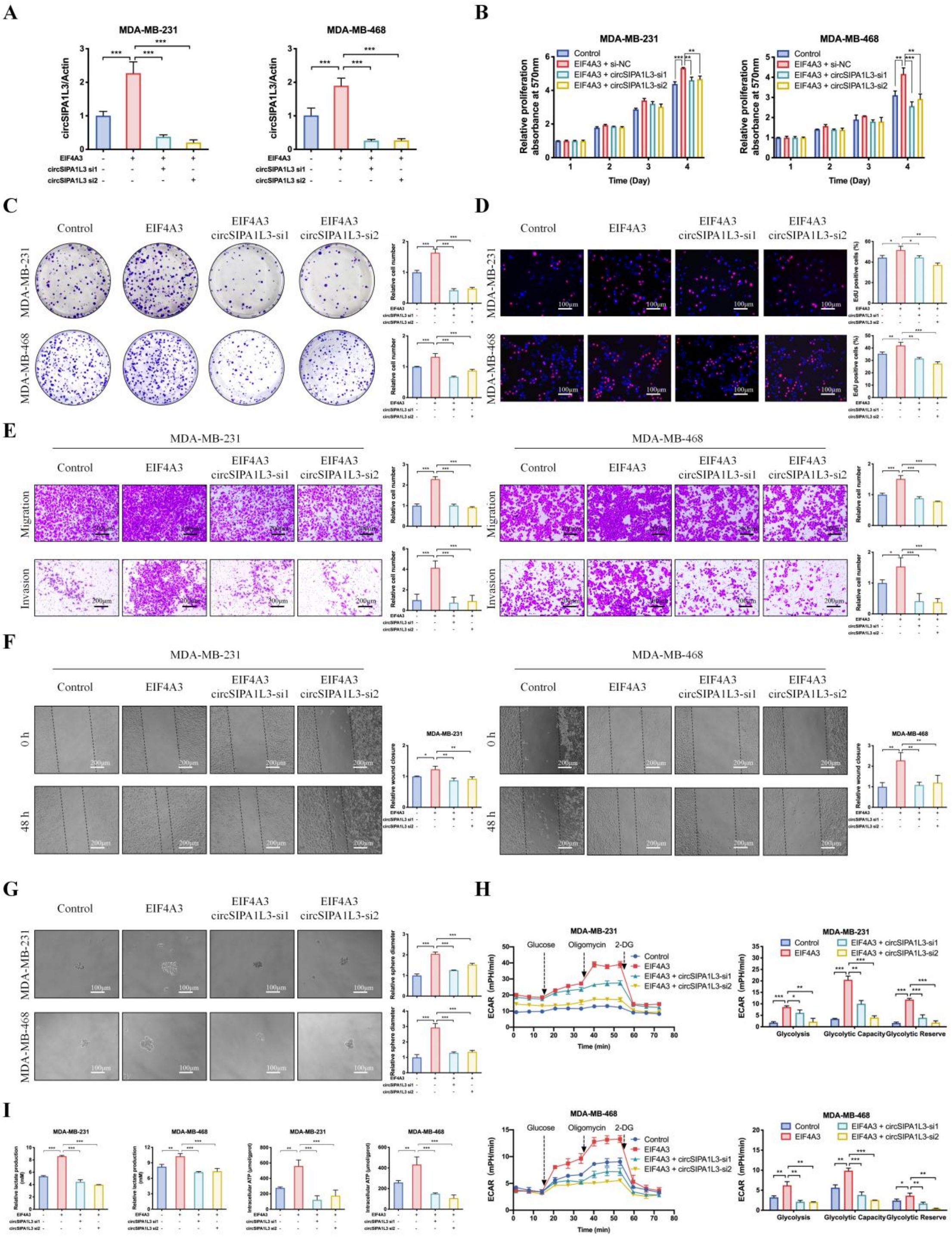
A
B
C
D
E
F
G
H
I
MDA-MB-231
MDA-MB-468
Control
EIF4A3
EIF4A3 circSIPA1L3-si1
EIF4A3 circSIPA1L3-si2
Migration
Invasion
0 h
48 h
Glucose
Oligomycin
2-DG
Time (min)
Time (Day)
Glycolysis
Glycolytic Capacity
Glycolytic Reserve

Supplementary Figure S12. EIF4A3 promotes glycolysis and progression through modulating circSIPA1L3 in breast cancer. A, qRT-PCR assays showed the expression of circSIPA1L3 in breast cancer cells co-transfected with EIF4A3 or circSIPA1L3 siRNAs. **B-D,** MTT (B), colony formation (C), and EdU (D) assays shows the proliferation ability of breast cancer cells co-transfected with EIF4A3 and circSIPA1L3 siRNAs as indicated. **E,** Transwell assay detects the migration and invasion abilities of breast cancer cells co-transfected with EIF4A3 and circSIPA1L3 siRNAs as indicated. **F,** Wound healing assay was used to evaluate the migration ability of breast cancer cells co-transfected with EIF4A3 and circSIPA1L3 siRNAs as indicated. **G,** Tumor sphere formation assay shows the stemness property of breast cancer cells co-transfected with EIF4A3 and circSIPA1L3 siRNAs as indicated. **H,** The extracellular acidification rate (ECAR) was detected in breast cancer cells co-transfected with EIF4A3 and circSIPA1L3 siRNAs as indicated, and glycolysis, glycolytic capacity, and glycolytic reserve of indicated cells were calculated. **I,** The lactate production and ATP levels were measured in breast cancer cells co-transfected with EIF4A3 and circSIPA1L3 siRNAs as indicated. (*P<0.05, **P<0.01, ***P<0.001)

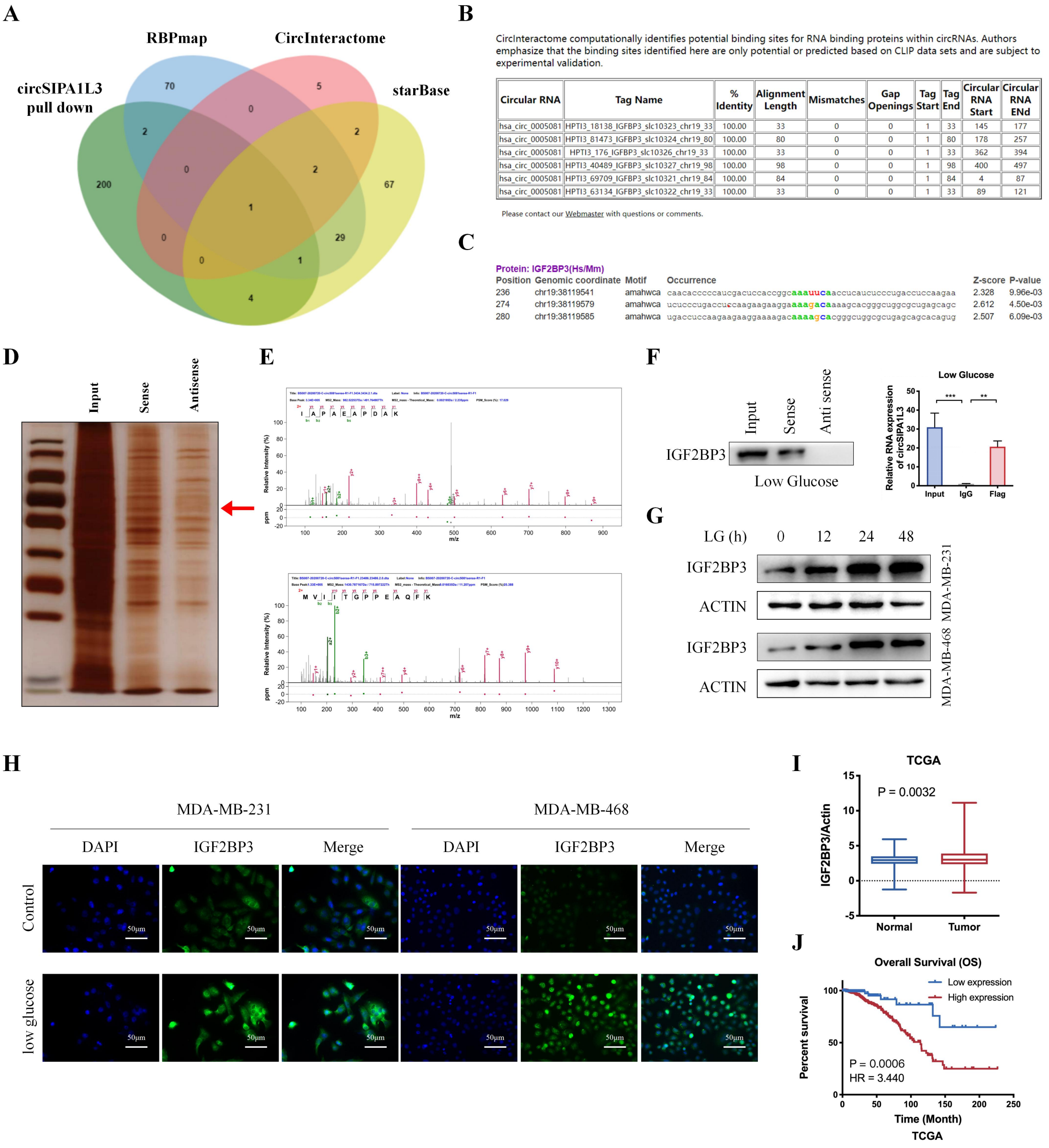
A
circSIPA1L3 pull down
RBPmap
CircInteractome
starBase
B
CircInteractome computationally identifies potential binding sites for RNA binding proteins within circRNAs. Authors emphasize that the binding sites identified here are only potential or predicted based on CLIP data sets and are subject to experimental validation.
Circular RNA | Tag Name | % Identity | Alignment Length | Mismatches | Gap Openings | Tag Start | Tag End | Circular RNA Start | Circular RNA ENd
hsa_circ_0005081 | HPTI3_18138_IGFBP3_slc10323_chr19_33 | 100.00 | 33 | 0 | 0 | 1 | 33 | 145 | 177
hsa_circ_0005081 | HPTI3_81473_IGFBP3_slc10324_chr19_80 | 100.00 | 80 | 0 | 0 | 1 | 80 | 178 | 257
hsa_circ_0005081 | HPTI3_176_IGFBP3_slc10326_chr19_33 | 100.00 | 33 | 0 | 0 | 1 | 33 | 362 | 394
hsa_circ_0005081 | HPTI3_40489_IGFBP3_slc10327_chr19_98 | 100.00 | 98 | 0 | 0 | 1 | 98 | 400 | 497
hsa_circ_0005081 | HPTI3_69709_IGFBP3_slc10321_chr19_84 | 100.00 | 84 | 0 | 0 | 1 | 84 | 4 | 87
hsa_circ_0005081 | HPTI3_63134_IGFBP3_slc10322_chr19_33 | 100.00 | 33 | 0 | 0 | 1 | 33 | 89 | 121
Please contact our Webmaster with questions or comments.
C
Protein: IGF2BP3(Hs/Mm)
Position | Genomic coordinate | Motif | Occurrence | Z-score | P-value
236 | chr19:38119541 | amahwca | caacacccccaucgacuccaccggcaaauucaaccucaucucccugaccuccaagaa | 2.328 | 9.96e-03
274 | chr19:38119579 | amahwca | ucucccugaccuccaagaagaaggaaaagacaaaagcacgggcuggcgcugagcagc | 2.612 | 4.50e-03
280 | chr19:38119585 | amahwca | ugaccuccaagaagaaggaaaagacaaaagcacgggcuggcgcugagcagcacagug | 2.507 | 6.09e-03
D
Input
Sense
Antisense
E
F
Input
Sense
Anti sense
IGF2BP3
Low Glucose
Low Glucose
Relative RNA expression of circSIPA1L3
Input
IgG
Flag
G
LG (h)
0
12
24
48
IGF2BP3
ACTIN
MDA-MB-231
MDA-MB-468
H
MDA-MB-231
MDA-MB-468
DAPI
IGF2BP3
Merge
Control
low glucose
50μm
I
TCGA
P = 0.0032
IGF2BP3/Actin
Normal
Tumor
J
Overall Survival (OS)
Low expression
High expression
Percent survival
P = 0.0006
HR = 3.440
Time (Month)
TCGA

Supplementary Figure S13. circSIPA1L3 physically interacts with IGF2BP3. A, Venn diagram shows the overlapping of interacted proteins with circSIPA1L3 among RNA pulldown results and predicted proteins using various databases. **B,** The potential binding sites for IGF2BP3 within circSIPA1L3 were predicted using CircInteractome database. **C,** The binding motif of IGF2BP3 were predicted in circSIPA1L3 sequence using RBPmap database. **D,** The protein bands detected by silver stain for mass spectrometry pulled down by circSIPA1L3 sense or anti-sense in breast cancer cells. The arrow points to IGF2BP3 band. **E,** The specific peptides of IGF2BP3 binding with circSIPA1L3 were identified by mass spectrometry. **F,** The binding between circSIPA1L3 and IGF2BP3 under low glucose condition was verified using RIP and RNA pulldown assay. **G**, The expression of IGF2BP3 under low glucose treatment for 0, 12, 24, and 48 h was evaluated by western blot. **H,** IF assay shows the expression of IGF2BP3 in breast cancer cells with or without low glucose treatment. **I,** The mRNA expression of IGF2BP3 in normal tissues and breast cancer tissues based on TCGA database. **J,** The association between IGF2BP3 expression and overall survival of breast cancer patients was analyzed using TCGA database. (**P<0.01, ***P<0.001)

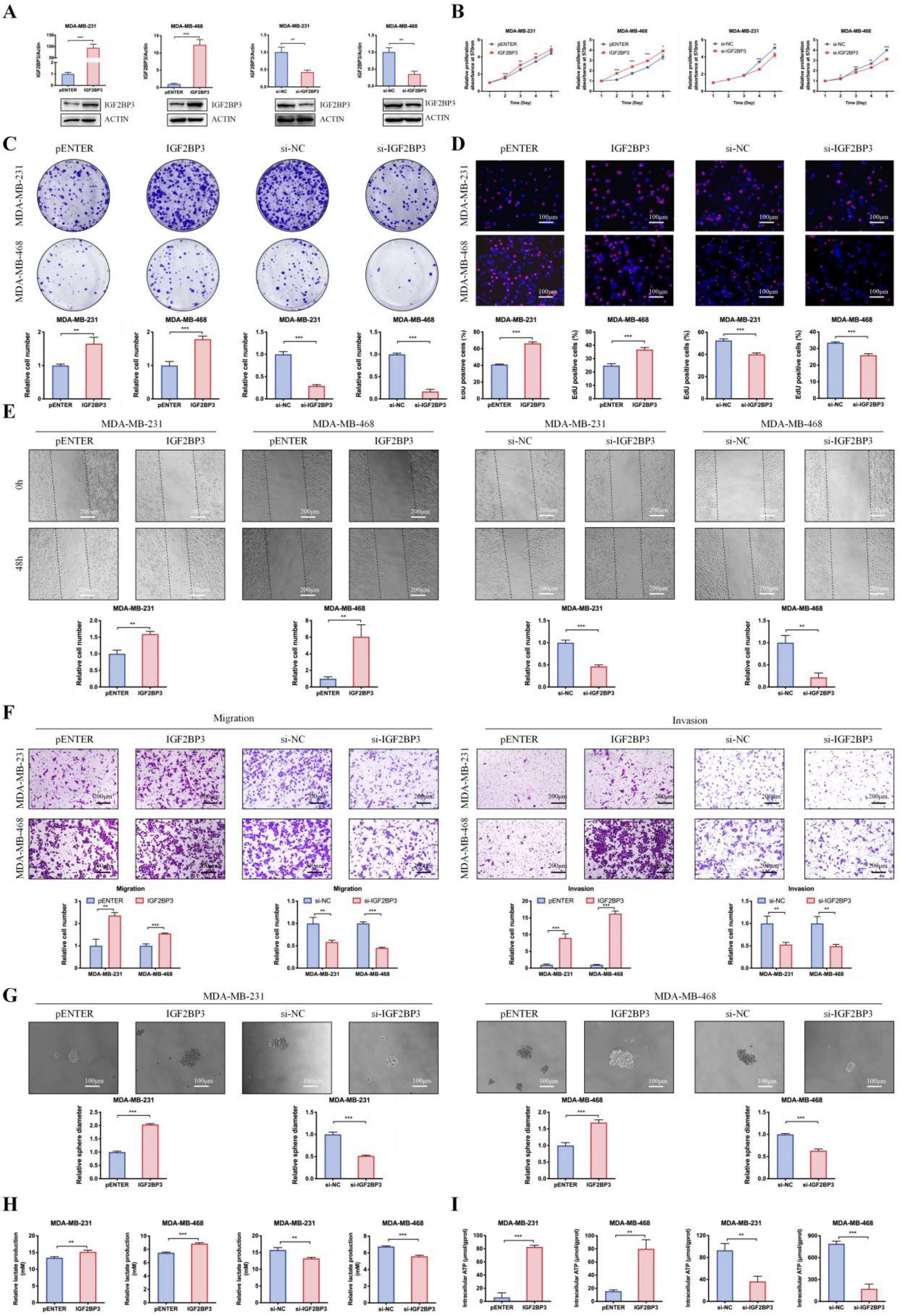
A
MDA-MB-231
MDA-MB-468
IGF2BP3
ACTIN
pENTER
si-NC
si-IGF2BP3
B
Time (Day)
C
pENTER
IGF2BP3
si-NC
si-IGF2BP3
MDA-MB-231
MDA-MB-468
Relative cell number
D
100μm
EdU positive cells (%)
E
0h
48h
200μm
Relative cell number
F
Migration
Invasion
200μm
G
100μm
Relative sphere diameter
H
Relative lactate production (mM)
I
Intracellular ATP (μmol/gprot)

Supplementary Figure S14. IGF2BP3 promotes proliferation, migration, invasion, stemness, and glycolysis of breast cancer cells. A, The transfection efficiency of IGF2BP3 overexpression or knockdown in breast cancer cells was detected using qRT-PCR (top) and western blot (bottom). **B-D,** MTT (B), colony formation (C), and EdU (D) assays were conducted to evaluate the effect of IGF2BP3 on cell proliferation. **E and F,** Wound healing (E) and transwell (F) assays were used to assess the migration and invasion abilities of breast cancer cells after IGF2BP3 overexpression or knockdown. **G,** Tumor sphere formation assay was performed to analyze the stemness of breast cancer cells after IGF2BP3 overexpression or knockdown**. H and I,** The lactate production (H) and ATP levels (I) were evaluated in breast cancer cells after IGF2BP3 overexpression or knockdown. (*P<0.05, **P<0.01, ***P<0.001)

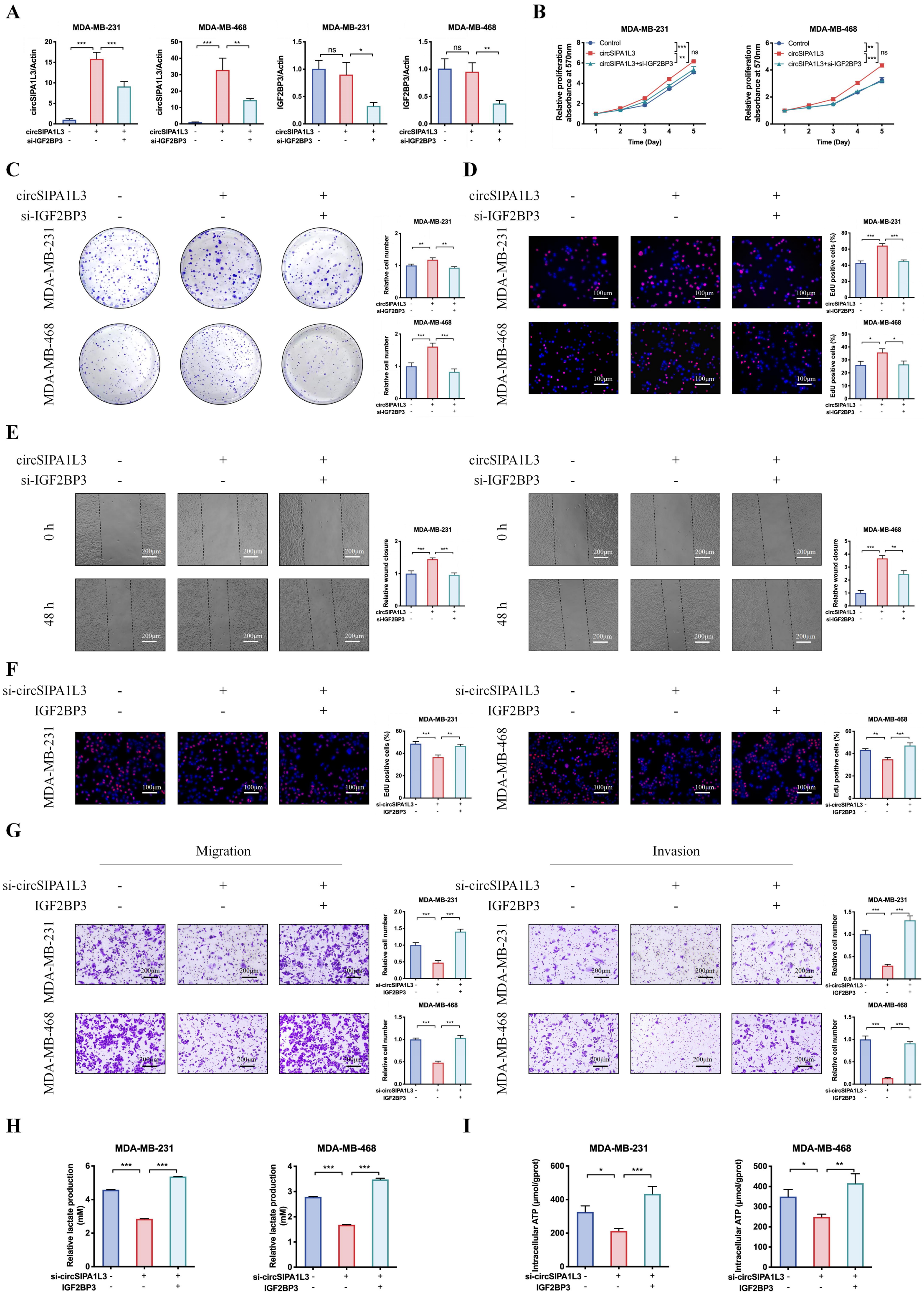

A
MDA-MB-231
MDA-MB-468
circSIPA1L3/Actin
IGF2BP3/Actin
circSIPA1L3
si-IGF2BP3
ns
B
Relative proliferation absorbance at 570nm
Control
circSIPA1L3
circSIPA1L3+si-IGF2BP3
Time (Day)
C
Relative cell number
D
EdU positive cells (%)
100μm
E
0 h
48 h
200μm
Relative wound closure
F
si-circSIPA1L3
IGF2BP3
G
Migration
Invasion
H
Relative lactate production (mM)
I
Intracellular ATP (μmol/gprot)

Supplementary Figure S15. circSIPA1L3 facilitated the malignant progression of breast cancer via regulating IGF2BP3. A, The RNA expression of circSIPA1L3 and IGF2BP3 in breast cancer cells co-transfected with circSIPA1L3 overexpressing plasmid and IGF2BP3 siRNA were detected by qRT-PCR. **B-D,** MTT (B), colony formation (C), and EdU (D) assays were performed to evaluate the proliferation of transfected breast cancer cells as indicated. **E,** Wound healing assay was used to evaluate the effect of IGF2BP3 knockdown on circSIPA1L-induced cell migration. **F-G**, EdU (**F**) and transwell (**G**) assays were performed to evaluate the proliferation, invasion and migration abilities of indicated transfected cells. **H-I**, The lactate secretion (**H**) and ATP production (**I**) of indicated transfected cells were detected. (ns, P > 0.05, *P < 0.05, **P < 0.01, ***P < 0.001)

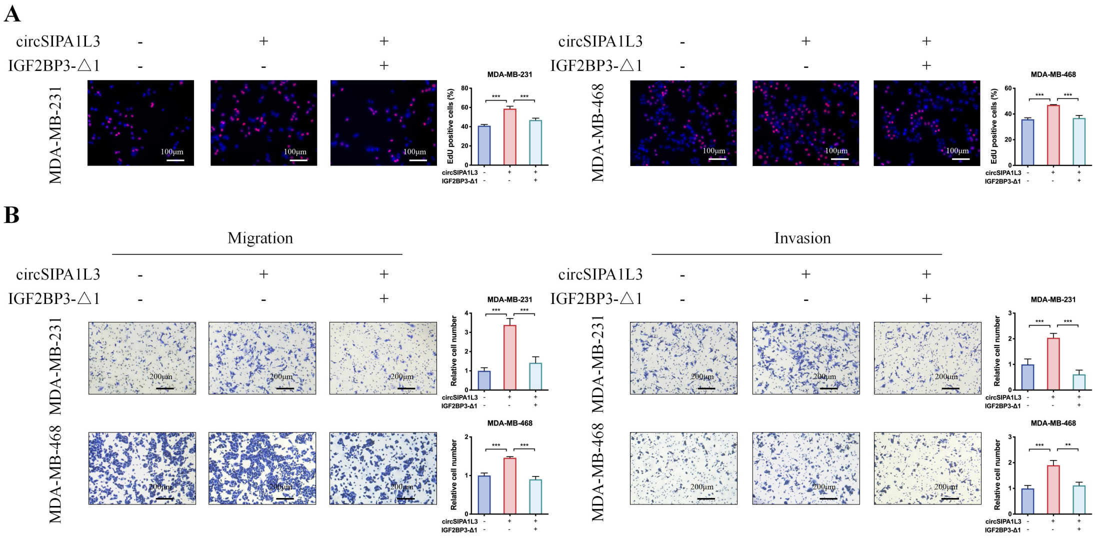

Supplementary Figure S16. Overexpression of the minimal binding unit between circSIPA1L3 and IGF2BP3 diminished the malignant effects of circSIPA1L3. A-B, EdU (**A**) and transwell (**B**) assays were performed to evaluate the proliferation, invasion and migration abilities of indicated transfected cells. (ns, P > 0.05, *P < 0.05, **P < 0.01, ***P < 0.001)

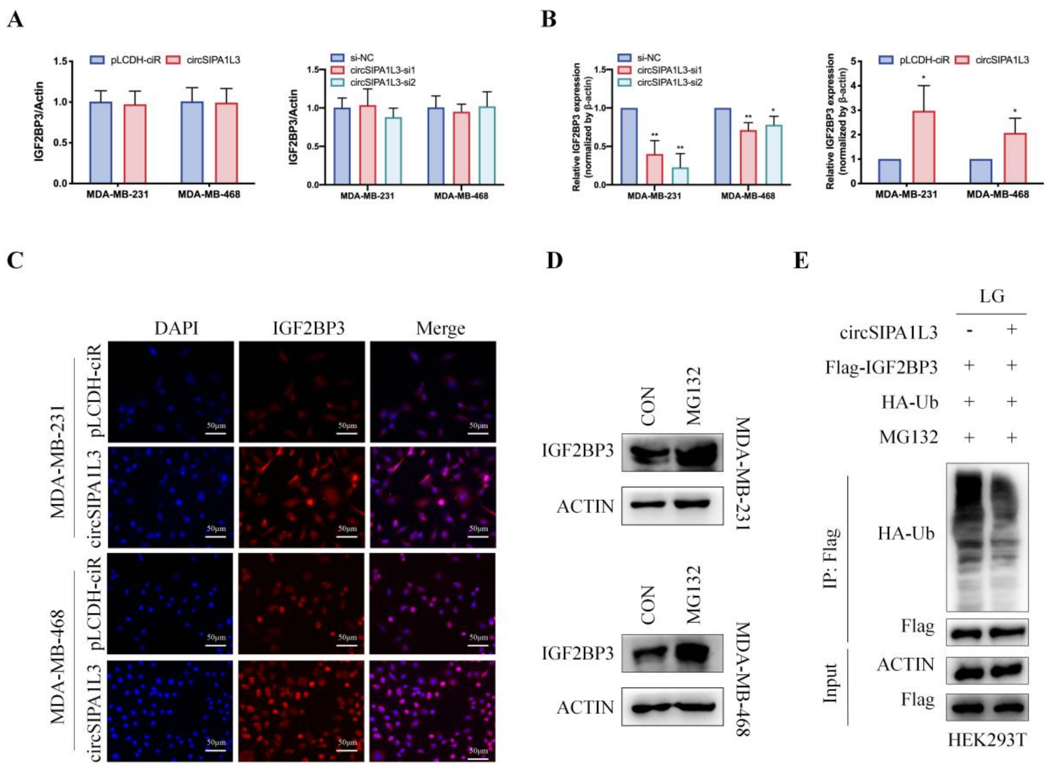

Supplementary Figure S17. circSIPA1L3 upregulated IGF2BP3 expression through inhibiting proteasome-mediated degradation. A, The mRNA level of IGF2BP3 was detected by qRT-PCR in breast cancer cells after circSIPA1L3 overexpression or knockdown. **B,** The effects of overexpression or knockdown of circSIPA1L3 on the IGF2BP3 protein level in breast cancer cells were analized by gray value using Image J. **C,** IF assay showed the expression of IGF2BP3 in breast cancer cells after circSIPA1L3 overexpression. **D,** Western blot was used to evaluate the protein level of IGF2BP3 in breast cancer cells with or without MG132 treatment. **E**, Co-IP and western blot assays were used to evaluate the effect of circSIPA1L3 overexpression on the ubiquitination of IGF2BP3 proteins under low glucose condition. (ns, $P > 0.05$, *$P < 0.05$, **$P < 0.01$, ***$P < 0.001$)

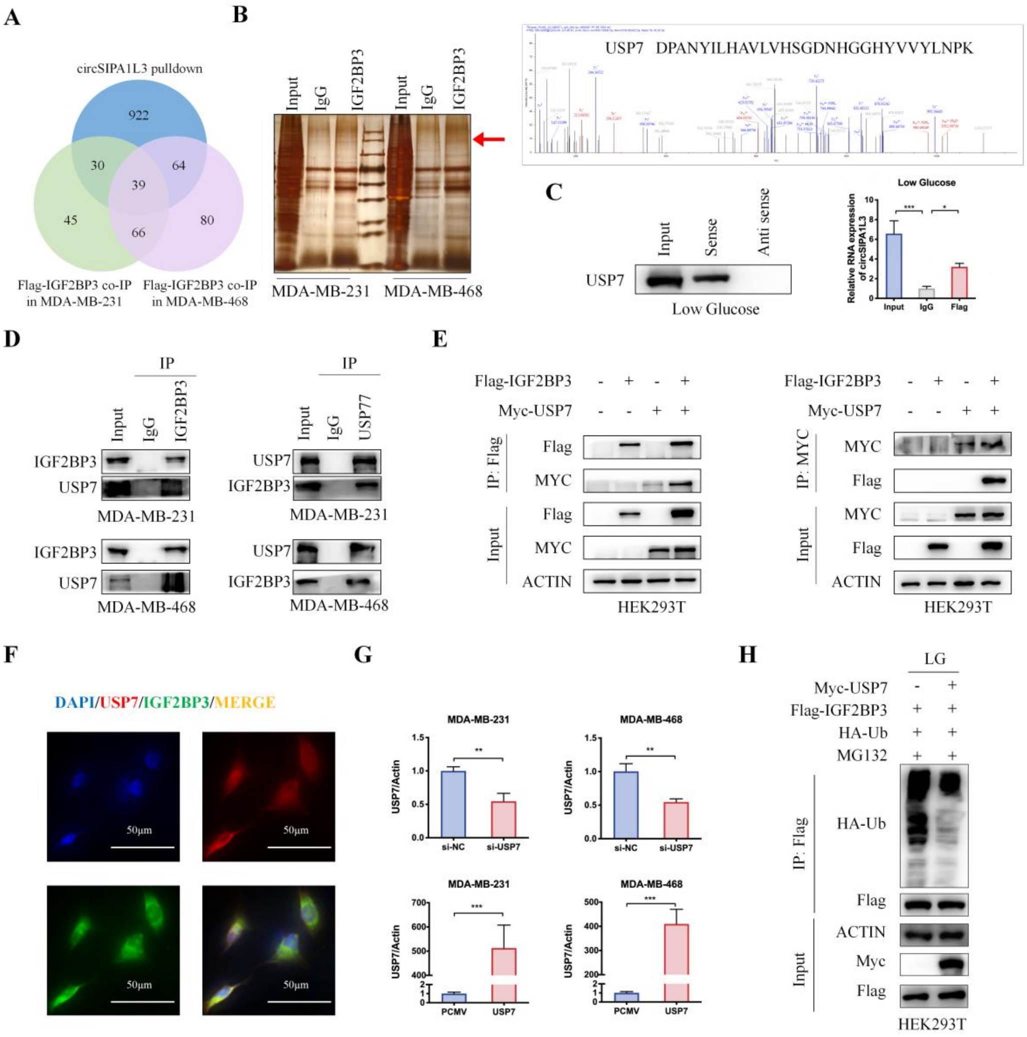
A
circSIPA1L3 pulldown
922
30
64
39
45
66
80
Flag-IGF2BP3 co-IP in MDA-MB-231
Flag-IGF2BP3 co-IP in MDA-MB-468
B
Input
IgG
IGF2BP3
MDA-MB-231
MDA-MB-468
USP7 DPANYILHAVLVHSGDNHGGHYVVYLNPK
C
Input
Sense
Anti sense
USP7
Low Glucose
Relative RNA expression of circSIPA1L3
IgG
Flag
D
IP
IGF2BP3
USP7
MDA-MB-231
MDA-MB-468
USP77
E
Flag-IGF2BP3
Myc-USP7
IP: Flag
IP: MYC
Flag
MYC
ACTIN
HEK293T
F
DAPI/USP7/IGF2BP3/MERGE
50μm
G
USP7/Actin
si-NC
si-USP7
PCMV
H
LG
HA-Ub
MG132
Myc

Supplementary Figure S18. circSIPA1L3 regulates IGF2BP3 through USP7. A, Venn diagram illustrates the candidate proteins based on MS analysis for RNA pulldown and co-IP assay. **B,** Co-IP assays followed by mass spectrometry were performed to identify the binding proteins using IGF2BP3 antibodies or IgG in breast cancer cells. The arrow points to USP7 band. The specific peptides of USP7 binding with IGF2BP3 were identified by mass spectrometry. **C,** The binding between circSIPA1L3 and USP7 under low glucose condition was verified using RIP and pulldown assay. **D,** The ability of endogenous IGF2BP3 to co-immunoprecipitate with endogenous USP7 in breast cancer cell lysates was verified by co-IP assays using anti-IGF2BP3 (left) or anti-USP7 (right) antibodies. **E,** HEK293T cells co-transfected with Flag-IGF2BP3 and Myc-USP7 were lysed, immunoprecipitated with anti-Flag (left) or anti-MYC (right) antibodies, and subjected to western blot analysis. **F,** The colocalization of IGF2BP3 and USP7 was confirmed by IF assay. **G,** The transfection efficiency of USP7 knockdown or overexpression in breast cancer cells was detected using qRT-PCR. **H,** Co-IP and western blot assays were used to evaluate the effect of USP7 overexpression on the ubiquitination of IGF2BP3 proteins under low glucose condition. (**P<0.01, ***P<0.001)

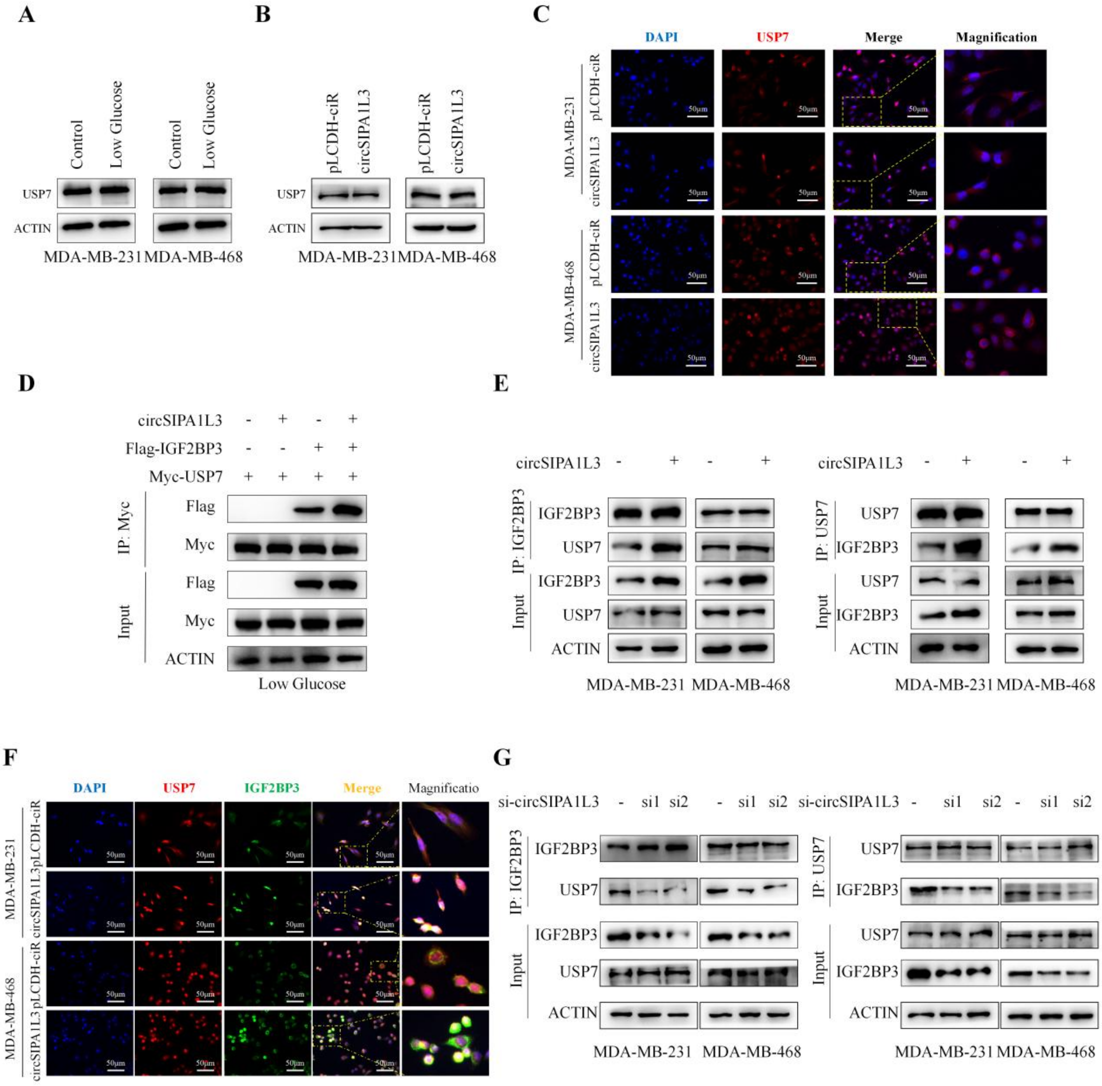
A
Control
Low Glucose
USP7
ACTIN
MDA-MB-231
MDA-MB-468
B
pLCDH-ciR
circSIPA1L3
C
DAPI
USP7
Merge
Magnification
50μm
D
circSIPA1L3
Flag-IGF2BP3
Myc-USP7
IP: Myc
Flag
Myc
Input
Low Glucose
E
IP: IGF2BP3
IGF2BP3
IP: USP7
F
IGF2BP3
Magnificatio
G
si-circSIPA1L3
si1
si2

Supplementary Figure S19. circSIPA1L3 promotes the interaction between IGF2BP3 and USP7. A, The effect of low glucose treatment on the protein level of USP7 was evaluated by western blot. **B**, The effect of circSIPA1L3 overexpression on the protein expression of USP7 was evaluated by western blot. **C,** IF assay was performed to analyze the expression of USP7 in breast cancer cells after circSIPA1L3 overexpression. **D,** The effect of circSIPA1L3 overexpression on the interaction between IGF2BP3 and USP7 under low glucose condition was detected using co-IP and western blot. **E**, Breast cancer cells were transfected with circSIPA1L3 plasmids or corresponding empty vectors, and co-IP assays using anti-IGF2BP3 (left) or anti-USP7 (right) antibodies were conducted. **F,** IF assay showed the co-localization of IGF2BP3 and USP7 in breast cancer cells after circSIPA1L3 overexpression. **G,** Co-IP assays evaluated the effect of circSIPA1L3 knockdown on the interaction between IGF2BP3 and USP7. (**P<0.01, ***P<0.001)

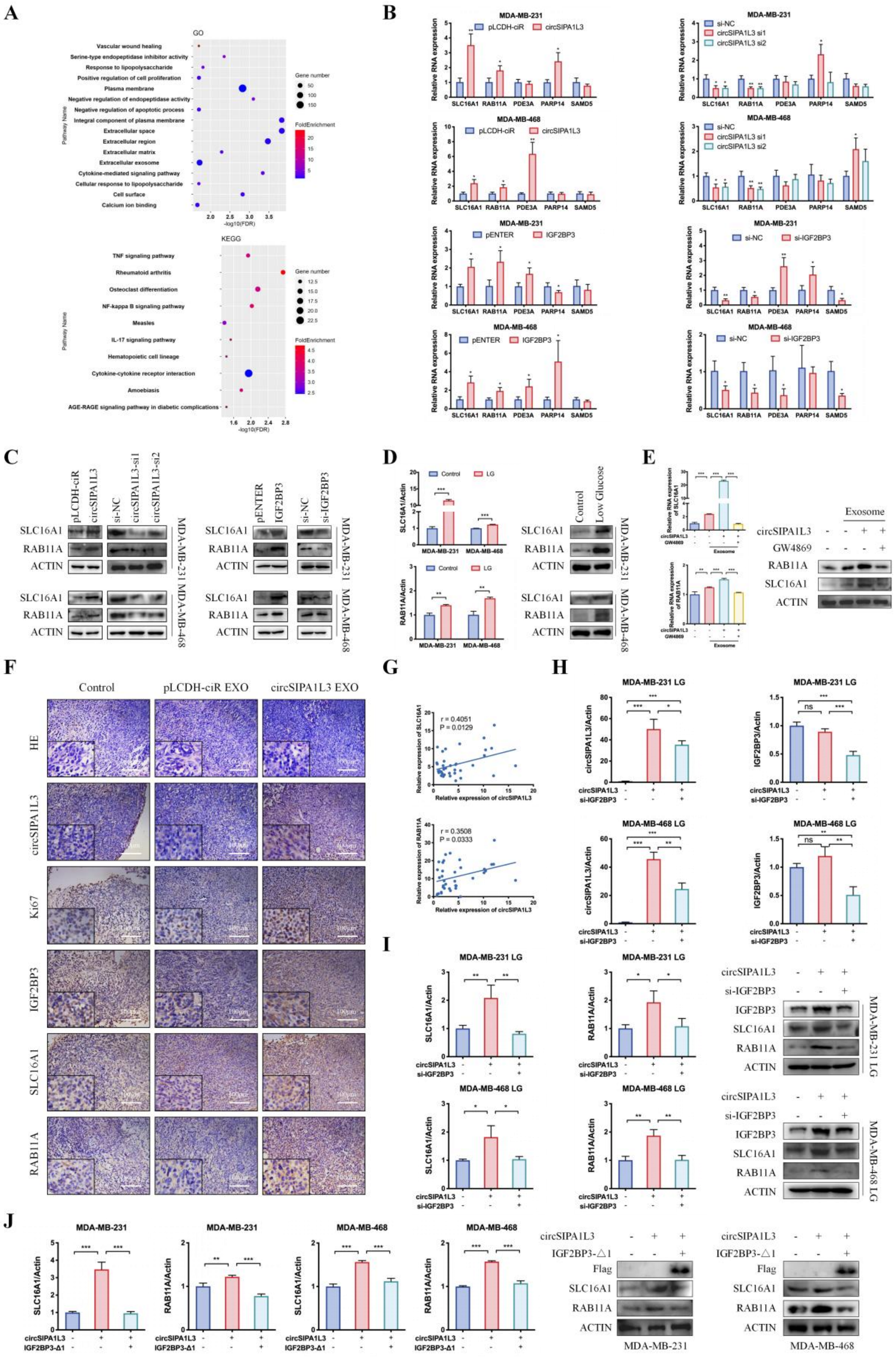

A
B
C
D
E
F
G
H
I
J
GO
KEGG
MDA-MB-231
MDA-MB-468
pLCDH-ciR
circSIPA1L3
si-NC
pENTER
IGF2BP3
si-IGF2BP3
SLC16A1
RAB11A
ACTIN
Control
Low Glucose
Exosome
GW4869
pLCDH-ciR EXO
circSIPA1L3 EXO
HE
Ki67
Flag
IGF2BP3-△1
MDA-MB-231 LG
MDA-MB-468 LG

Supplementary Figure S20. circSIPA1L3 promotes the expression of SLC16A1/RAB11A. A, GO (left) and KEGG (right) analysis showed the biological processes or pathways enriched in differentially expressed genes. **B,** qRT-PCR was used to detect the RNA expression levels of potential target genes of circSIPA1L3/IGF2BP3. **C,** The protein levels of SLC16A1 and RAB11A were detected by western blot in breast cancer cells after circSIPA1L3/IGF2BP3 overexpression or knockdown. **D**, The effect of low glucose treatment on the RNA and protein levels of SLC16A1 and RAB11A. **E**, The RNA and protein levels of SLC16A1 and RAB11A in breast cancer cells treated with exosomes derived from cells with or without GW4869 treatment. **F,** Representative images showing the results of HE, ISH (circSIPA1L3), and IHC (Ki67, IGF2BP3, SLC16A1, and RAB11A) of xenograft tumor tissues from different groups. **G**, The correlation analysis between the expression of SLC16A1/RAB11A and circSIPA1L3 in breast cancer tissues. **H**, The co-transfection efficiency of circSIPA1L3-overexpressing plasmid and si-IGF2BP3 in breast cancer cells under low glucose condition was evaluated by qRT-PCR. **I**, The effect of circSIPA1L3 overexpression and IGF2BP3 knockdown on the RNA and protein levels of SLC16A1 and RAB11A under low glucose condition. **J**, The RNA and protein levels of SLC16A1 and RAB11A in indicated transfected breast cancer cells. (*P<0.05, **P<0.01, ***P<0.001)

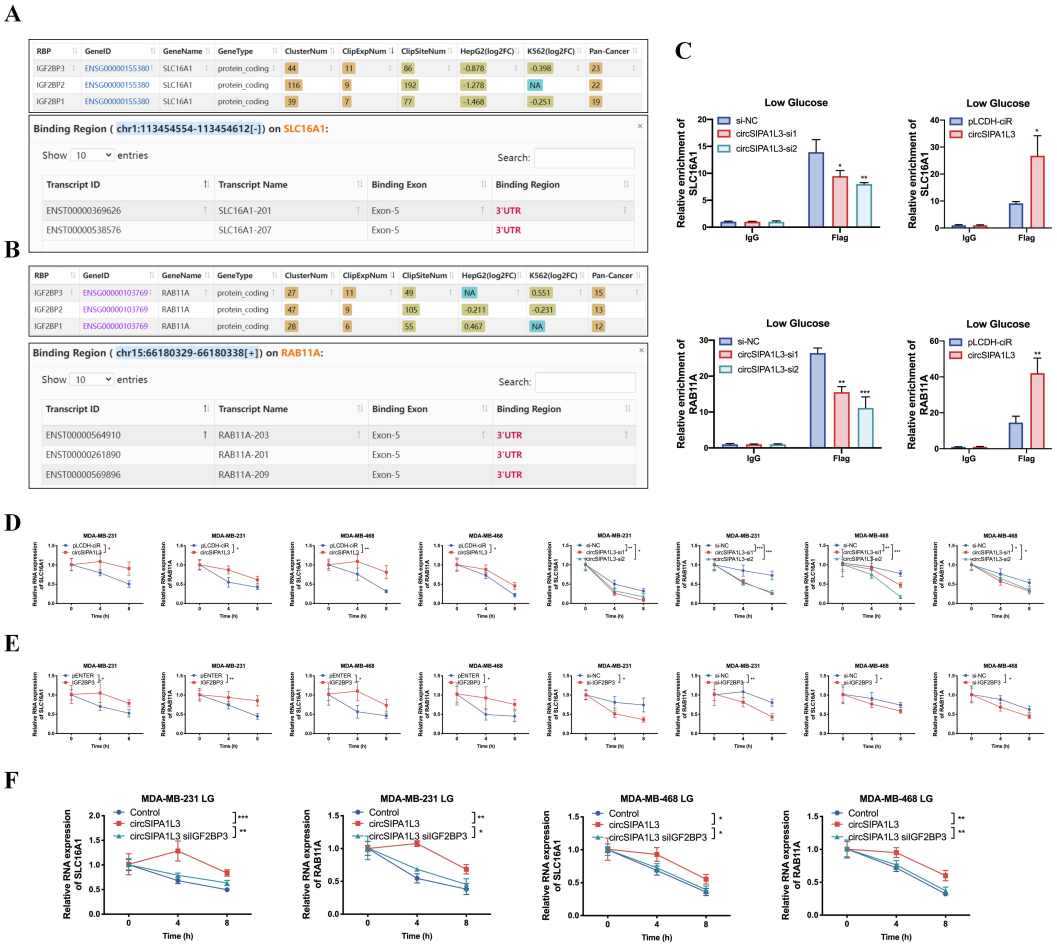
A
RBP GeneID GeneName GeneType ClusterNum ClipExpNum ClipSiteNum HepG2(log2FC) K562(log2FC) Pan-Cancer
IGF2BP3 ENSG00000155380 SLC16A1 protein_coding 44 11 86 -0.878 -0.398 23
IGF2BP2 ENSG00000155380 SLC16A1 protein_coding 116 9 192 -1.278 NA 22
IGF2BP1 ENSG00000155380 SLC16A1 protein_coding 39 7 77 -1.468 -0.251 19
Binding Region (chr1:113454554-113454612[-]) on SLC16A1:
Show 10 entries
Search:
Transcript ID Transcript Name Binding Exon Binding Region
ENST00000369626 SLC16A1-201 Exon-5 3'UTR
ENST00000538576 SLC16A1-207 Exon-5 3'UTR
B
RBP GeneID GeneName GeneType ClusterNum ClipExpNum ClipSiteNum HepG2(log2FC) K562(log2FC) Pan-Cancer
IGF2BP3 ENSG00000103769 RAB11A protein_coding 27 11 49 NA 0.551 15
IGF2BP2 ENSG00000103769 RAB11A protein_coding 47 9 105 -0.211 -0.231 13
IGF2BP1 ENSG00000103769 RAB11A protein_coding 28 6 55 0.467 NA 12
Binding Region (chr15:66180329-66180338[+]) on RAB11A:
Show 10 entries
Search:
Transcript ID Transcript Name Binding Exon Binding Region
ENST00000564910 RAB11A-203 Exon-5 3'UTR
ENST00000261890 RAB11A-201 Exon-5 3'UTR
ENST00000569896 RAB11A-209 Exon-5 3'UTR
C
Low Glucose
Relative enrichment of SLC16A1
Relative enrichment of RAB11A
si-NC
circSIPA1L3-si1
circSIPA1L3-si2
pLCDH-ciR
circSIPA1L3
IgG
Flag
D
E
MDA-MB-231
MDA-MB-468
Relative RNA expression of SLC16A1
Relative RNA expression of RAB11A
pENTER
IGF2BP3
si-IGF2BP3
Time (h)
F
MDA-MB-231 LG
MDA-MB-468 LG
Control
circSIPA1L3
circSIPA1L3 siIGF2BP3
Relative RNA expression of SLC16A1
Relative RNA expression of RAB11A
Time (h)

Supplementary Figure S21. circSIPA1L3 stabilizes SLC16A1/RAB11A mRNA through regulating IGF2BP3. A, The IGF2BPs-SLC16A1 mRNA interactions were supported by CLIP-seq data (top). The 3'UTR of SLC16A1 mRNA contains multiple IGF2BP3 binding motifs (bottom). **B,** The IGF2BPs-RAB11A mRNA interactions were supported by CLIP-seq data (top). The 3'UTR of RAB11A mRNA contains multiple IGF2BP3 binding motifs (bottom). **C**, The relative enrichment of IGF2BP3 in the 3'UTR of SLC16A1 and RAB11A mRNA under low glucose condition was detected by RIP assay after circSIPA1L3 knockdown or overexpression. **D,** The mRNA stability of SLC16A1/RAB11A was analyzed after circSIPA1L3 overexpression or knockdown. **E,** The mRNA stability of SLC16A1/RAB11A was evaluated after IGF2BP3 overexpression or knockdown. **F**, The mRNA stability of SLC16A1 and RAB11A under low glucose condition was assessed by qRT-PCR in breast cancer cells co-transfected with circSIPA1L3 overexpressing plasmids and IGF2BP3 siRNA as indicated upon ActD treatment. (*P<0.05, **P<0.01, ***P<0.001)

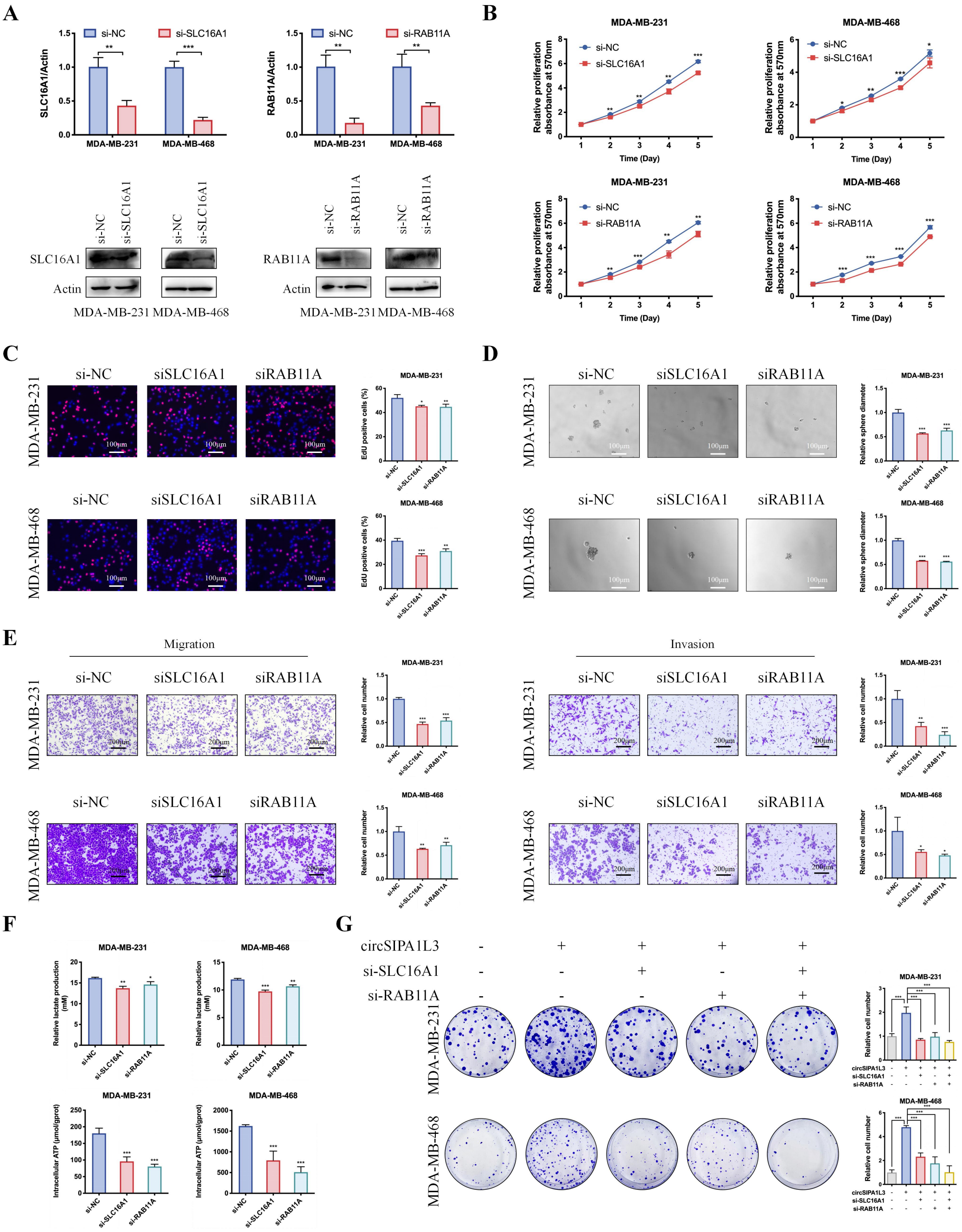

A
B
C
D
E
F
G
si-NC
si-SLC16A1
si-RAB11A
SLC16A1/Actin
RAB11A/Actin
MDA-MB-231
MDA-MB-468
SLC16A1
RAB11A
Actin
Relative proliferation absorbance at 570nm
Time (Day)
siSLC16A1
siRAB11A
EdU positive cells (%)
Relative sphere diameter
Migration
Invasion
Relative cell number
Relative lactate production (mM)
Intracellular ATP (μmol/gprot)
circSIPA1L3

Supplementary Figure S22. SLC16A1/RAB11A promotes proliferation, migration, invasion, stemness, and glycolysis of breast cancer cells. A, The transfection efficiency of SLC16A1 or RAB11A knockdown in breast cancer cells was detected using qRT-PCR (top) and western blot (bottom). **B and C,** MTT (B) and EdU (C) assays were conducted to evaluate the effect of SLC16A1/RAB11A knockdown on cell proliferation. **D,** Tumor sphere formation assay was performed to analyze the stemness of breast cancer cells after SLC16A1 or RAB11A knockdown**. E,** Transwell assay was used to assess the migration and invasion abilities of breast cancer cells after SLC16A1 or RAB11A knockdown. **F,** The lactate production and ATP level were evaluated in breast cancer cells after SLC16A1 or RAB11A knockdown. **G,** Breast cancer cells were transfected with pLCDH-ciR, circSIPA1L3, si-NC, or si-SLC16A1/RAB11A alone or simultaneously, and the abilities of cell proliferation was assessed by colony formation assay. (*P<0.05, **P<0.01, ***P<0.001)

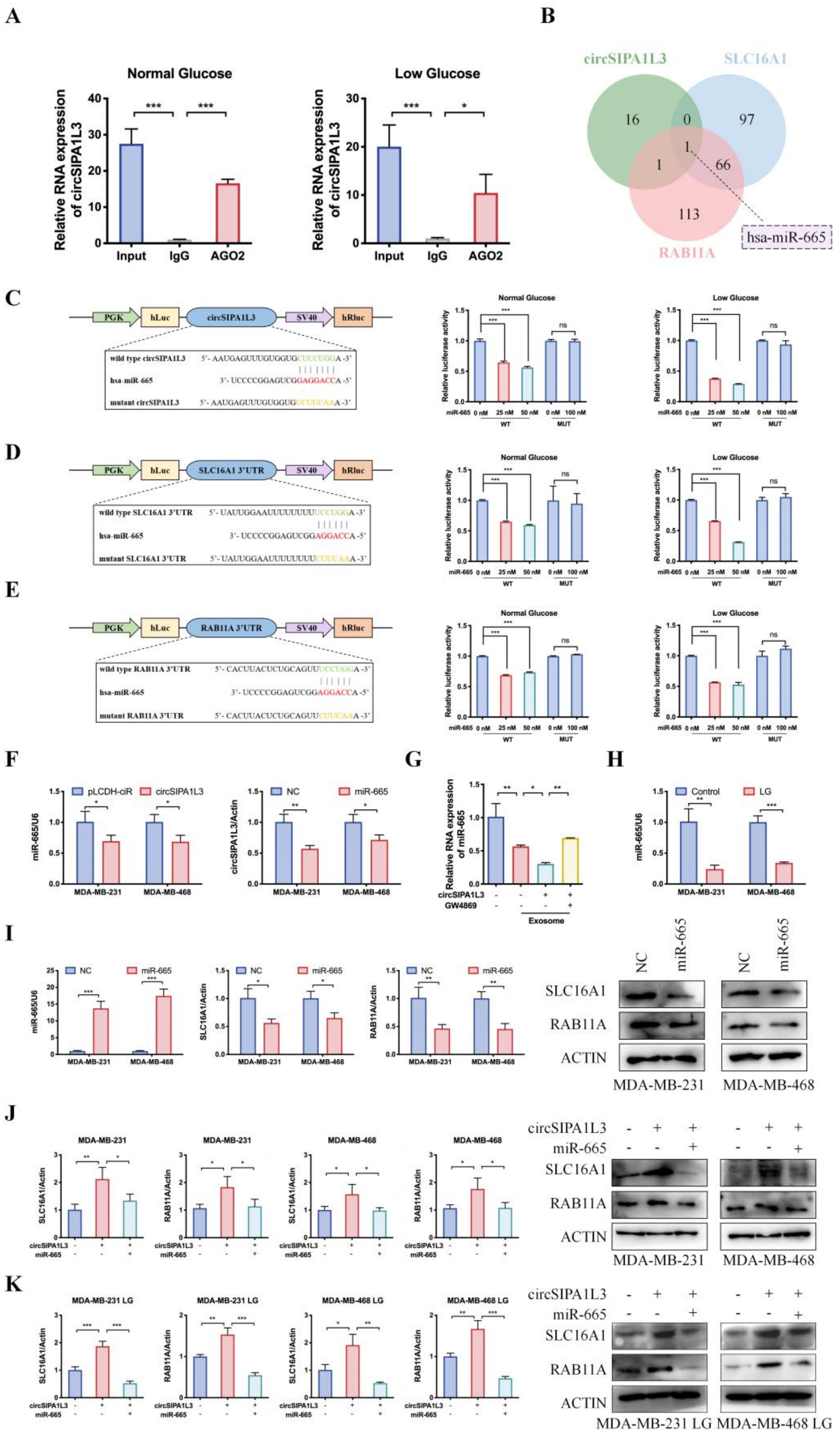

Supplementary Figure S23. circSIPA1L3 promotes SLC16A1/RAB11A expression through sponging miR-665. A, RIP assay was performed to assess the association between circSIPA1L3 and AGO2 in normal or low glucose condition. **B,**

Venn diagram exhibits the overlapping of potential target miRNAs of circSIPA1L3, SLC16A1, and RAB11A based on starBase database. **C,** Schematic illustration of the WT and mutant (MUT) binding site sequences in circSIPA1L3 for miR-665 (left). Dual luciferase reporter gene assay was used to detect the interaction between circSIPA1L3 and miR-665 in normal or low glucose condition (right). **D,** Schematic illustration of the WT and mutant (MUT) binding site sequences in SLC16A1 3'UTR for miR-665 (left). Dual luciferase reporter gene assay was used to detect the interaction between SLC16A1 3'UTR and miR-665 in normal or low glucose condition (right). **E,** Schematic illustration of the WT and mutant (MUT) binding site sequences in RAB11A 3'UTR for miR-665 (left). Dual luciferase reporter gene assay was used to detect the interaction between RAB11A 3'UTR and miR-665 in normal or low glucose condition (right). **F,** The mutual influence between circSIPA1L3 and miR-665 was evaluated by qRT-PCR. **G,** The level of miR-665 in breast cancer cells treated with exosomes derived from cells with or without GW4869 treatment. **H**, The effect of low glucose treatment on the miR-665 level in breast cancer cells. **I**, The transfection efficiency of miR-665 mimics (left), and the RNA (middle) and protein (right) expression of SLC16A1 and RAB11A after miR-665 overexpression were evaluated. **J,** Expression of SLC16A1 or RAB11A RNA (left) and protein (right) after treatment with circSIPA1L3 and miR-665 mimics were measured. **K,** Expression of SLC16A1 or RAB11A RNA (left) and protein (right) after treatment with circSIPA1L3 and miR-665 mimics under low glucose condition were measured. (ns, $P > 0.05$, *$P < 0.05$, **$P < 0.01$, ***$P < 0.001$)

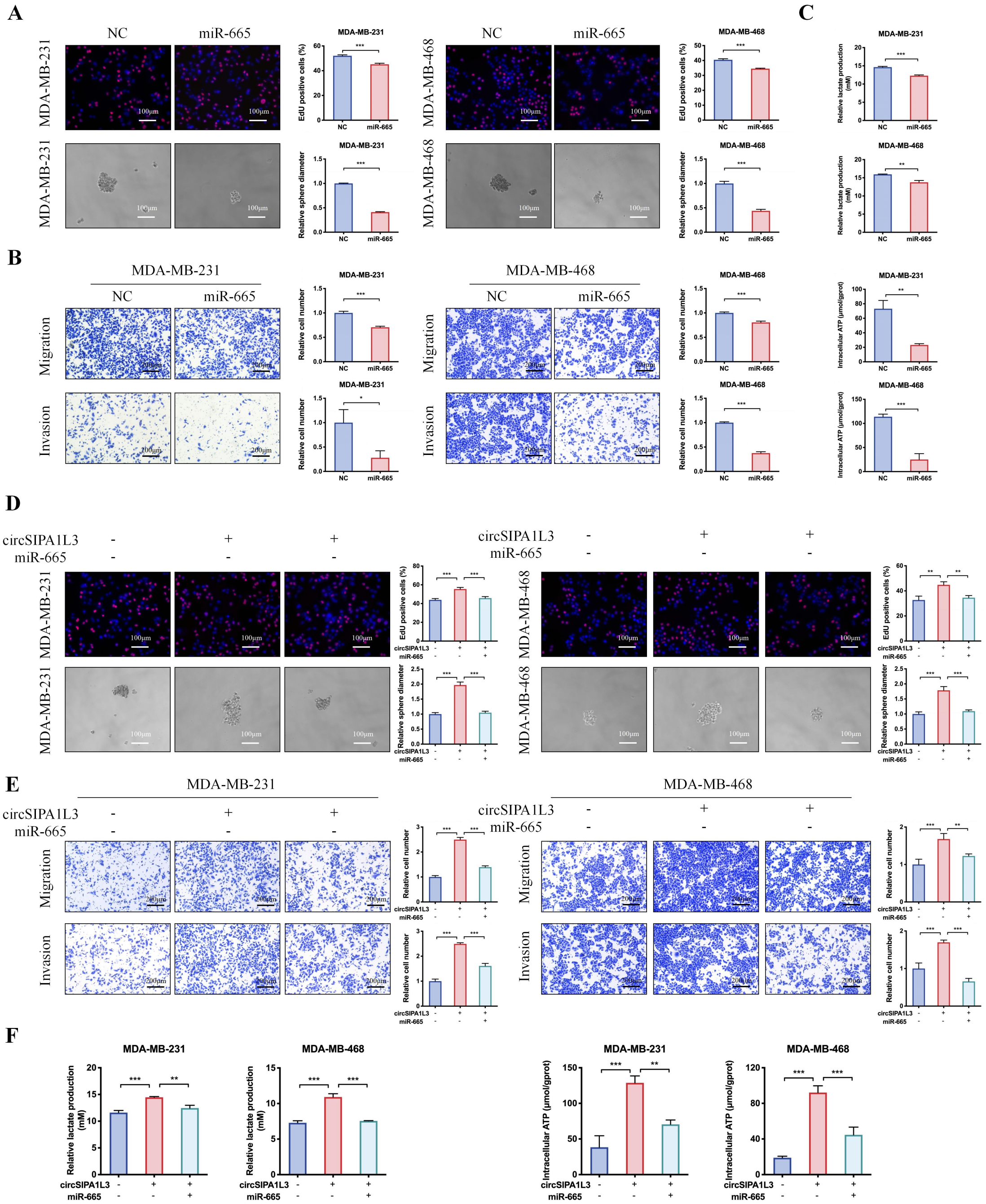

Supplementary Figure S24. circSIPA1L3 promotes malignant progression of breast cancer partially through sequestering miR-665. A, The cell proliferation (top) and tumor stemness (bottom) of breast cancer cells were analyzed after miR-665 overexpression. **B,** Transwell assay was used to evaluate the effect of miR-665 overexpression on cell migration and invasion. **C,** The lactate production and ATP levels were measured in breast cancer cells after miR-665 overexpression. **D-F,** Breast cancer cells were co-transfected with circSIPA1L3 overexpression plasmids and miR-665 mimics. **D,** EdU assay (top) and tumor sphere formation assay (bottom) were performed to investigate the proliferation and stemness of breast cancer cells. **E,** The migration and invasion abilities of breast cancer cells were evaluated by transwell assay. **F,** The lactate production and ATP levels were detected in breast cancer cells. (**P<0.01, ***P<0.001)

Supplementary Tables

Table S1. The clinicopathological features of breast cancer patients.

**Characteristics**	**Total cases (n = 238)**	**circSIPA1L3 expression level**		
		**Low**	**High**	***p* value**
**Age**				
≤ 50	123	65	58	
> 50	115	72	43	0.164033
**Menopausal state**				
Pre	115	60	55	
Post	123	77	46	0.134839
**Pathological grade**				
≤ 2	163	96	67	
> 2	75	41	34	0.636862
**ER**				
Positive	153	105	48	
Negative	85	32	53	$\boldsymbol{p < 0.001}$
**PR**				
Positive	140	92	48	
Negative	98	45	53	**0.00364**
**HER2**				
Positive	41	29	12	
Negative	197	108	89	0.088842
**Ki67**				
Low	60	40	20	
High	178	97	81	0.133936
**Tumor size**				
≤ 2	110	63	47	
> 2	128	74	54	0.962094
**Lymph node metastasis**				
Positive	116	69	47	
Negative	122	68	54	0.650472
**Metastasis status**				
Positive	34	13	21	
Negative	204	124	80	**0.022875**

Abbreviation: LN=lymph nodes; ER=estrogen receptor; PR=progesterone receptor; HER-2: human epidermal growth factor receptor-2; p value < 0.05 marked in bond font to show statistical significant.

Table S2. Univariate and Multivariate analyses for the overall survival of 238 breast cancer patients with complete clinicopathological information.

	Univariate analysis [a]		Multivariate analysis [b]	
	HR (95% CI)	*p* value	HR (95% CI)	*p* value
**Age [c]**	1.027(0.984-1.071)	0.226		
**Menopausal status (pre vs. post)**	0.751(0.297-1.904)	0.547		
**Pathological Grade (> G2 vs. G1&G2)**	2.334(0.926-5.882)	0.072		
**ER status (ER+ vs. ER-)**	0.641(0.253-1.626)	0.349		
**PR status (PR+ vs. PR-)**	0.819(0.323-2.075)	0.673		
**HER2 status (HER2+ vs. HER2-)**	0.61(0.14-2.653)	0.509		
**Ki67 positive rate (High vs. low)**	2.804(0.645-12.199)	0.169		
**Tumor size [c]**	1.486(1.052-2.101)	**0.025**	1.41(1.013-1.963)	**0.042**
**Lymph node status (with metastasis vs. without metastasis)**	5.494(1.59-18.98)	**0.007**		
**Metastasis status (with metastasis vs. without metastasis)**	59.674(13.704-259.856)	**＜0.001**	41.705(8.848-196.571)	**＜0.001**
**5081 Expression (High expression vs. low expression)**	1.331(0.525-3.372)	**0.004**	3.143(1.013-9.75)	**0.047**

a. Cox's proportional hazards regression model was utilized.

b. Only variables with $p < 0.05$ in univariate analysis were incorporated into the multivariate Cox proportional hazard regression analysis. HR, hazard ratio; CI, confidence interval.

c. Age and tumor size were considered as continuous variables.

p value < 0.05 marked in bond font to show statistical significant.

Table S3. siRNA and mimics used for transfection.

Name	sense （5’-3’）	antisense （5’-3’）
circSIPA1L3 si1	AGACUGAAGUAUGGCUGUG	CACAGCCAUACUUCAGUCU
circSIPA1L3 si2	CUGAAGUAUGGCUGUGACC	GGUCACAGCCAUACUUCAG
si-EIF4A3	GUGGCCAUUAACUUUGUAA	UUACAAAGUUAAUGGCCAC
si-IGF2BP3	GCAGGAAUUGACGCUGUAU	AUACAGCGUCAAUUCCUGC
si-USP7	GGGCACCAUACCCAAAUUA	UAAUUUGGGUAUGGUGCCC
si-RAB11A	GGAGUAGAGUUUGCAACAA	UUGUUGCAAACUCUACUCC
si-SLC16A1	GGUAAUUGGAGCUUUCAUU	AAUGAAAGCUCCAAUUACC
miR-665 mimics	ACCAGGAGGCUGAGGCCCCU	GGGCCUCAGCCUCCUGGUUU
NC	UUCUCCGAACGUGUCACGU	ACGUGACACGUUCGGAGAA

Table S4. Primers used for qRT-PCR.

Gene	Forward （5’-3’）	Reverse （5’-3’）
circSIPA1L3	CTACAGGCGACAGAGGGTTC	GTCGGATTTGCGGAATGTGG
circ1185	ACTCCACCAAGCATCCCATTA	TTTGTTCCAAAGCAGTTGTGC
circ4682	TGGCAAGACCAGATCCTTGA	TGGCCCAGTTTCCTCCATAG
circ91203	TGGCAAGACCAGATCCTTGA	TGGCCCAGTTTCCTCCATAG
circ9035	TCAGTTCATGATGGTGGAGCA	TGGCAGCTATGCTGTTGTCT
circ8557	GGCTACCTCAAAATGGACAACC	GCATTTCCCCTTAGTGATGAGC
SIPA1L3-F	GCCGAGGTTGAGGACTATGGGTT	GGATGATGACCACCTTCACAG
EIF4A3	TGACCGACCCAATCCG	GTCAGCCAGTCCACCTTTC
RIP-circ-EIF-F1	GAAGTGTCTTAGTGCCTTTCTTC	GAATGTGGTTCCACTGGGGATG
RIP- circ -EIF-F2	CGCAAATCCGACGTCTTCAGAG	ATACTCCTGGCGGGTCCTGGTG
RIP- circ -EIF-F3	ATTCAACCTCATCTCCCTGACCTCC	TAGTCCTGGGCCACCACCCTCC
RIP- circ -EIF-F4	AAATCTTCTATGGACGAGGAGACC	GGCTAGGGAAGTATTTCAGTGGTT
RIP- circ -EIF-F5	CTTCCTCTGGAAGGTTTGTGCC	AGCCTCCTAAAGTGCTGAGATAGATTAC
IGF2BP3	CGGAACATCACCAAACA	GACTTACAAGCCGCAGA
USP7	GTCACGATGACGACCTGTC	ATCGCTCCACCAACTGCTG
SAMD5	TCCTTCGTGGATAACGGCTACG	GTTGGCGTCCTGCTCCCGCA
PARP14	CTGTCGCTATGTGCTTCACG	GACAAGCTCTCAGTGATCTC
SLC16A1	TGTTGGTGGCTGCTTGTCAG	CATGGTCAGAGCTGGATTCAAG
PDE3A	AGAGCCTCTGAGGAAAGCAT	TGCTCCATAATTGAGTCCAGG
RAB11A	CACCATTGGAGTAGAGTTTGC	GGCACCTACAGCTCCACGAT
Actin	CACTGTGCCCATCTACGAG	AATGTCACGCACGATTTCC
GAPDH	GGAGCGAGATCCCTCCAAAAT	GGCTGTTGTCATACTTCTCATGG
miR-665	ACCAGGAGGCTGAGGCCCCT	CAGTGCGTGTCGTGGAGT
U6	CTCGCTTCGGCAGCACA	AACGCTTCACGAATTTGCGT

Table S5. Antibodies used in the experiments.

Antigen	Supplier	Catalog #	Application
GM130	Cell Signaling Technology	12480	IB (1:1000)
HSP70	Cell Signaling Technology	4873	IB (1:1000)
Calnexin	Cell Signaling Technology	2679	IB (1:1000)
CD9	Cell Signaling Technology	98327	IB (1:1000)
HK1	Proteintech	19662-1-AP	IB (1:1000) IHC(1:200)
HK2	Proteintech	22029-1-AP	IB (1:1000) IHC(1:200)
ALDOA	Proteintech	11217-1-AP	IB (1:1000)
LDHA	Proteintech	21799-1-AP	IB (1:1000) IHC(1:200)
GLUT4	Proteintech	66846-1-Ig	IB (1:1000) IHC(1:200)
PFKP	Proteintech	13389-1-AP	IB (1:1000) IHC(1:200)
Fibronectin	Proteintech	15613-1-AP	IB (1:2000) IHC(1:300)
Vimentin	Cell Signaling Technology	5741	IB (1:1000) IF(1:200) IHC(1:200)
N-cadherin	Cell Signaling Technology	13116	IB (1:1000) IHC(1:100)
E-cadherin	Cell Signaling Technology	14472	IB (1:1000) IHC(1:200)
SLUG	Proteintech	12129-1-AP	IB (1:1000)
OCT4	Proteintech	11263-1-AP	IB (1:1000) IF(1:200) IHC(1:200)
SOX2	Proteintech	11064-1-AP	IB (1:1000) IF(1:200) IHC(1:200)
NANOG	Proteintech	67255-1-Ig	IB (1:1000) IHC(1:200)
β-catenin	Proteintech	51067-2-AP	IB (1:1000)
NF-κB	Proteintech	10745-1-AP	IB (1:1000)
p-IKβ-α	Cell Signaling Technology	5209S	IB (1:1000)

Iκβ-α	Proteintech	10268-1-AP	IB (1:1000)
AKT	Proteintech	60203-2-Ig	IB (1:1000)
p-AKT	Proteintech	66444-1-Ig	IB (1:1000)
STAT3	Proteintech	10253-2-AP	IB (1:1000)
p-STAT3	Cell Signaling Technology	9145	IB (1:1000)
EIF4A3	Proteintech	17504-1-AP	IB (1:1000) IF(1:200) IHC(1:200)
IGF2BP3	Proteintech	14642-1-AP	IB (1:1000) IF(1:200) IHC(1:200)
USP7	Proteintech	66514-1-Ig	IB (1:1000) IF(1:200)
SLC16A1	Proteintech	20139-1-AP	IB (1:500) IHC(1:300)
RAB11A	Proteintech	15903-1-AP	IB (1:500) IHC(1:300)
MYC Tag Antibody	Thermo Fisher	PA1-981	IB (1:1000)
DYKDDDDK Tag Antibody	Thermo Fisher	MA1-142-A488	IB (1:1000)
HA Tag Antibody	Thermo Fisher	PA1-985	IB (1:1000)
Ki67	Cell Signaling Technology	9449	IHC(1:1000)
CD31	Proteintech	11265-1-AP	IHC(1:1000)
Actin	Proteintech	60008-1-Ig	IB (1:2000)
HRP-anti-mouse	Cell Signaling Technology	7076	IB (1:5000)
HRP-anti-rabbit	Cell Signaling Technology	7074	IB (1:5000)
